# The hearing hippocampus

**DOI:** 10.1016/j.pneurobio.2022.102326

**Published:** 2022-11

**Authors:** Alexander J. Billig, Meher Lad, William Sedley, Timothy D. Griffiths

**Affiliations:** a UCL Ear Institute, University College London, London, UK; b Translational and Clinical Research Institute, Newcastle University Medical School, Newcastle upon Tyne, UK; c Biosciences Institute, Newcastle University Medical School, Newcastle upon Tyne, UK; d Wellcome Centre for Human Neuroimaging, UCL Queen Square Institute of Neurology, University College London, London, UK; e Human Brain Research Laboratory, Department of Neurosurgery, University of Iowa Hospitals and Clinics, Iowa City, USA

**Keywords:** Hippocampus, Hearing, Auditory cognition, Sound, Medial temporal lobe, Auditory, Perception

## Abstract

The hippocampus has a well-established role in spatial and episodic memory but a broader function has been proposed including aspects of perception and relational processing. Neural bases of sound analysis have been described in the pathway to auditory cortex, but wider networks supporting auditory cognition are still being established. We review what is known about the role of the hippocampus in processing auditory information, and how the hippocampus itself is shaped by sound. In examining imaging, recording, and lesion studies in species from rodents to humans, we uncover a hierarchy of hippocampal responses to sound including during passive exposure, active listening, and the learning of associations between sounds and other stimuli. We describe how the hippocampus' connectivity and computational architecture allow it to track and manipulate auditory information – whether in the form of speech, music, or environmental, emotional, or phantom sounds. Functional and structural correlates of auditory experience are also identified. The extent of auditory-hippocampal interactions is consistent with the view that the hippocampus makes broad contributions to perception and cognition, beyond spatial and episodic memory. More deeply understanding these interactions may unlock applications including entraining hippocampal rhythms to support cognition, and intervening in links between hearing loss and dementia.

## Highlights

• Hippocampus responds to sound during passive exposure, active listening and learning.
• Auditory experience shapes the structure and function of hippocampus.
• Sound can entrain hippocampal oscillations, with potential clinical implications.
• Recording, imaging and lesion studies are reviewed in species from mouse to human.

## 1 Introduction and motivation

Given the two most well-known functions of the hippocampus – supporting episodic memory in humans (Scoville and Milner, 1957, Squire and Zola-Morgan, 1991) and spatial navigation in animals (O’Keefe and Dostrovsky, 1971) – a review of this structure in relation to sound may seem an unlikely exercise. In humans, cortical circuits underlying auditory perception and cognition are largely found in lateral, rather than medial temporal lobe structures (Bizley and Cohen, 2013, Griffiths and Warren, 2004, Rauschecker and Tian, 2000, Schnupp et al., 2013). Although the hippocampus has access to highly processed information from all sensory modalities, it is often conceptualized as sitting atop a visual cortical hierarchy (Felleman and Van Essen, 1991, Turk-Browne, 2019) and its role in auditory memory in primates has been challenged on anatomical and functional grounds (Fritz et al., 2005, Munoz-Lopez et al., 2010).

However, theories of hippocampal function have long extended beyond episodic memory and spatial navigation (Kimble, 1968, Papez, 1937). One idea is that the hippocampus is important for the binding of arbitrary relations and mediating their flexible expression (Cohen and Eichenbaum, 1993, Lisman et al., 2017). This extended job description encompasses linking multimodal objects with a spatiotemporal, environmental, or cognitive context to form episodic memories (Yonelinas et al., 2019), supporting short-term memory (Hannula and Ranganath, 2008, Pertzov et al., 2013), associating disparate elements of a scene (Graham et al., 2010, Maguire and Mullally, 2013, Olsen et al., 2012), structuring conceptual knowledge (Behrens et al., 2018), and forming predictions (Stachenfeld et al., 2017). Strong versions of such accounts might allow for involvement of the hippocampus in a range of situations involving auditory information, such as binding acoustic features into a perceptual whole, anticipating the continuation of sentences or melodies, and "mental navigation" along sequences of auditory stimuli. We shall see that the computational circuitry of the hippocampus is well suited for operating on information organized in time - such as that carried by acoustic signals. In light of this extended proposed functional scope, a full account of the auditory system should at least consider the hippocampus.

There are also practical and clinical motivations for understanding interactions between sound and the hippocampus. Auditory stimulation can entrain hippocampal rhythms, with implications for enhancing memory and mitigating cognitive decline (Derner et al., 2018, Harrington and Cairney, 2021, Martorell et al., 2019). Auditory signals interact with hippocampal memories, an effect that could be harnessed to boost learning (Cousins et al., 2016, Crowley et al., 2019) or target pathological memories in a clinical setting (Ressler et al., 2021). Hippocampal structure and function are also shaped by experience, raising the question of how auditory expertise and deprivation affect the hippocampus, for example playing a role in tinnitus (Kraus and Canlon, 2012; L. Zhang et al., 2019) or mediating a link between hearing loss and dementia (Griffiths et al., 2020, Livingston et al., 2017). Addressing these issues and realizing therapeutic potential requires a consolidation of knowledge about pathways that carry information between auditory sites and hippocampus.

Recognizing the relative preservation of hippocampal anatomy and physiology across species, and the advances in understanding function this affords (Buffalo, 2015, Clark and Squire, 2013, Cohen and Eichenbaum, 1993, Witter and Amaral, 2021), we include studies from rodents to primates. Electrophysiological and neuroimaging data are considered alongside neuropsychological and animal lesion work. We begin by outlining the anatomy of the hippocampus and the pathways connecting it with canonical auditory structures. We then characterize hippocampal responses to meaningless sounds, going on to consider how these change as sounds signal value or acquire task-relevance. Circumstances under which the hippocampus supports different types of association are set out, with a focus on interactions between processing of sound, time, and space. This leads to a consideration of how the hippocampus might support the formation and retrieval of objects, scenes, and memories that are purely auditory. We cover the special cases of speech, music, emotional sounds and phantom percepts, then set out how auditory experience affects hippocampal structure and function.

Dominant accounts of hippocampal function, as well as key physiological properties, are briefly introduced as required, but readers are referred to detailed reviews (and for a short primer might consult Knierim, 2015). We describe known computational principles of the hippocampus to the extent that they account for auditory data. A key question throughout is to what degree hippocampal involvement in sound processing is secondary to or dependent on its established roles in episodic memory and spatial navigation. Another is the extent to which the hippocampus automatically processes auditory information as opposed to any requirement for the information to be relevant to behavior. We shall find the concept of the hippocampus as a predictive map useful for drawing together some of the findings. However, rather than attempting an integrated theory of the hippocampus through the prism of sound, our aim is to highlight the range of circumstances under which it processes and is shaped by auditory signals. In essence, we are not "claiming" the hippocampus as an auditory structure so much as examining how its computational architecture might be engaged in and altered by auditory tasks.

## 2 Anatomy and auditory-hippocampal pathways

**Fig. 1:**
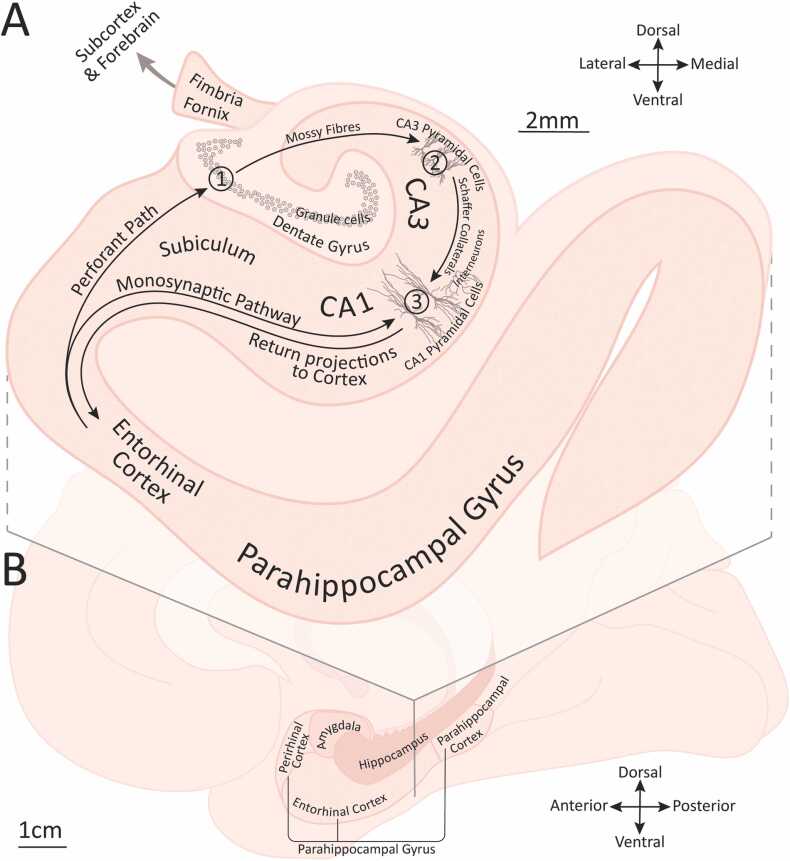
(A) Coronal cross-section of human medial temporal lobe showing parahippocampal gyrus, entorhinal cortex, hippocampus with primary subfields, and key pathways. Numbers 1–3 indicate synapses of the trisynaptic pathway. Pyramidal cells, interneurons and granule cells not shown to scale. (B) Medial sagittal view of human brain showing medial temporal lobe structures (including amygdala) and indicating the position of the cross-section shown in A.

Fig. 1A shows the two interlocking gyri of the hippocampus - the cornu ammonis (including subfields CA1 and CA3) and dentate gyrus (DG) - extending postero-anteriorally in the medial temporal lobe of primates, and dorso-ventrally just below neocortex in rodents. The major cortical input to this bilateral structure is from adjacent entorhinal cortex (ERC), which in primates forms part of the parahippocampal gyrus. A well-described pathway, the trisynaptic loop, projects from ERC through DG, CA3, and CA1 back to ERC, from where output is routed back to neocortex. There are also direct projections from ERC to CA1 (the monosynaptic pathway) and extensive recurrent connections within CA3. The hippocampus is reciprocally connected via the fornix to thalamus, mammillary bodies, and the basal forebrain, as well as to amygdala, basal ganglia, cingulate, and frontal and parietal lobes. We will see later how computations associated with these pathways may be relevant to auditory processing.

Along with ERC, the parahippocampal gyrus in primates consists of perirhinal and parahippocampal (postrhinal in rodents) cortices, which connect to ERC from anterior and posterior directions respectively as shown in Fig. 1B (for detailed connections see Burwell and Amaral, 1998; Garcia and Buffalo, 2020; Munoz-Lopez et al., 2010; Nilssen et al., 2019; van Strien et al., 2009 and other anatomical studies in Supplementary Table A). Fellemann & Van Essen (1991) show the hippocampus at the apex of a visual cortical hierarchy, with parahippocampal and perirhinal cortices exchanging information with high-order areas in the ventral visual pathway. The functional anatomy of the primate auditory system is less well mapped than that of vision, at least downstream of primary cortex beyond the lemniscal path from cochlea through the cochlear nucleus, inferior colliculus, and medial geniculate body of the thalamus. A dorsal pathway (sometimes termed a "where" stream due to its role in audiospatial processing) runs from posterior auditory cortex via parietal sites to dorsolateral prefrontal cortex (Rauschecker and Scott, 2016). More ventrally, a "what" stream courses anteriorly along superior temporal gyrus, superior temporal sulcus, and middle temporal gyrus, with features extracted and represented that are increasingly abstract and removed from the acoustic signal. This ventral pathway is often described as terminating in ventrolateral frontal cortex, however, at least in monkeys, additional projections from those anterior temporal sites via the temporal pole reach perirhinal, parahippocampal and entorhinal cortices, which in turn connect to hippocampus (Munoz-Lopez et al., 2015, Munoz-Lopez et al., 2010). Auditory information has multiple opportunities along this series of synapses to be integrated with that from other modalities. There are also somewhat more direct projections from association (belt or parabelt) cortex to entorhinal/perirhinal/parahippocampal cortex in macaques, and from primary cortex to perirhinal and entorhinal cortices in rodents, although these may be sparser for audition than in other sensory modalities (Amaral et al., 1983, Burwell and Amaral, 1998, Munoz-Lopez et al., 2010, Suzuki and Amaral, 1994, Yi et al., 2022). Efferent pathways from the medial temporal lobe trace similar routes back as the afferent connections described (Muñoz and Insausti, 2005, Tranel et al., 1988, Vaudano et al., 1991) but - in rodents at least - are supplemented by others, such as from hippocampus direct to primary auditory cortex (Cenquizca and Swanson, 2007) and even inferior colliculus (Olthof et al., personal communication).

**Fig. 2:**
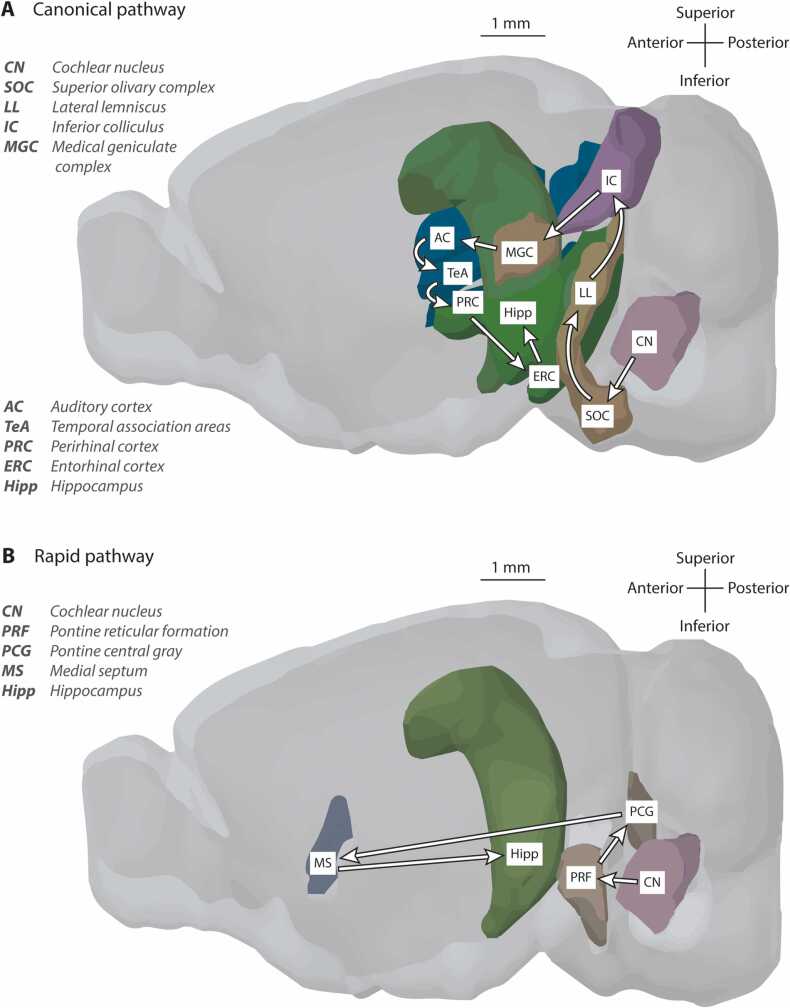
Lateral view of mouse brain showing pathways from auditory brainstem structures to hippocampus. (A) Canonical pathway consisting of at least ten synapses and (B) A rapid five-synapse pathway. Adapted from Allen Reference Atlas - Mouse Brain (available from atlas.brain-map.org).

In rodents, subcortical pathways also carry auditory information to the hippocampal formation, including one from cochlear nucleus via pontine nuclei and medial septum (Xiao et al., 2018; G.-W. Zhang et al., 2018), and another from thalamus via basolateral amygdala and entorhinal cortex (Bordi and LeDoux, 1994, LeDoux et al., 1985, Wahlstrom et al., 2018). That these bear auditory information is evidenced by early latency auditory responses at hippocampus and entorhinal cortex, prior to those occurring in auditory cortex, described in Section 3. Anatomical connections from medial septum to entorhinal cortex have also been traced in non-human primates (Insausti et al., 1987). These subcortical pathways may provide fast, indiscriminate communication of the presence of sound, in contrast to slower cortical routes conveying more elaborated representations of a sound and its meaning, including after integrating information from other sensory modalities (Rolls, 1996). See Fig. 2 for two ascending auditory-hippocampus pathways in the mouse, and Kraus and Canlon (2012) for more detail on the interaction between the auditory system and other medial temporal lobe structures.

Establishing the extent to which both these standard and non-canonical pathways in rodents and non-human primates are mirrored in humans is difficult. In the absence of axonal tract tracing or post-mortem studies, indirect measures of structural connectivity such as diffusion tensor imaging (DTI) can be informative. A neurosurgical atlas based on data from the human connectome project highlighted the absence of direct connections between medial and lateral temporal structures, although indirect pathways were functionally established that correspond to some of those outlined in non-human primates, such as from auditory association areas on the superior temporal gyrus to posterior parahippocampal fields then entorhinal cortex (Baker et al., 2018). One ultra-high-resolution DTI study found white matter tracts between hippocampus and both the temporal pole and planum polare, but not low-level auditory cortex (Maller et al., 2019). Another identified connections between hippocampus and a region of interest that included both auditory core and belt areas (Jang and Choi, 2022) in a majority of subjects. Differences across the results of these studies may relate to the thresholds used in the probabilistic tractography procedure.

Complementing structural approaches like DTI that identify anatomical tracts, functional-connectivity analysis defines correlated time series between areas, from which direct or indirect connections can be inferred. Such analysis of resting-state blood-oxygen-level-dependent (BOLD) activity measured with functional magnetic resonance imaging (fMRI) has revealed distinct connectivity between different parts of the hippocampus and neocortical regions. Whereas activity in posterior hippocampus and parahippocampal cortex correlates with activity in lateral parietal cortex and midline sites, activity in anterior hippocampus and perirhinal/entorhinal cortex correlates with that in lateral temporal regions including superior temporal gyrus extending to temporal pole (Kahn et al., 2008; S.-F. Wang et al., 2016). Clustering of more temporally-resolved functional connectivity patterns derived from intracranially recorded high-frequency resting state activity found anterior and medial temporal sites, including hippocampus, to have the strongest coupling with auditory cortex (Banks et al., 2022). While that finding included primary auditory cortex, other resting state intracranial and fMRI studies of connections between medial temporal and sensory cortex emphasize those between hippocampus and *association* areas in humans, with the possible exception of olfaction, compared to the primary sites that dominate in rodents (Bergmann et al., 2016, Zhou et al., 2021).

Electrophysiological and fMRI studies coupled with electrical or optogenetic stimulation also reveal pathways relevant to auditory processing. The hippocampus orchestrates activity across cortex, propagating theta oscillations (4–7 Hz in rodents) for temporal control of information processing, and sharp-wave ripples for memory consolidation (Buzsáki, 2015, Buzsáki, 2002; see Section 8). For example, neurons in guinea pig inferior colliculus and auditory cortex phase lock to hippocampal theta both during spontaneous firing and in response to sound (Liberman et al., 2009, Pedemonte et al., 2001, Pedemonte et al., 1996). In cats, brief electrical stimulation of dorsal hippocampus at a theta rate enhances auditory cortical responses to subsequent clicks (Parmeggiani and Rapisarda, 1969). Beyond theta, optogenetic stimulation at lower (1 Hz; Chan et al., 2017) and higher (40 Hz; Weitz et al., 2015) rates in rat hippocampus influences BOLD activity in auditory cortex. Electrical stimulation at even faster rates in rabbits led to an increase in the amplitude of click responses in motor cortex (Cazard and Buser, 1963), while other studies in cats found electrical stimulation of hippocampus leading to reduced auditory cortical responses to brief medial geniculate body electrical pulses (Redding, 1967), and reduced click responses in cerebellum (Fox et al., 1967) and hypothalamus (Feldman and Dafny, 1968). Single-pulse electrical stimulation of hippocampus even in the absence of an auditory stimulus elicits rapid responses in the auditory cortex not only of cats (Parmeggiani and Rapisarda, 1969) but also of humans. In the latter case these occur not only in auditory association areas on the lateral temporal lobe (Catenoix et al., 2011, Enatsu et al., 2015) but also primary auditory cortex in Heschl's gyrus, with initial responses as early as 10 ms (Rocchi et al., 2021). These electrical and optogenetic stimulation studies provide further evidence for anatomical and functional links from hippocampus to auditory cortex.

The influence of the hippocampus on auditory processing elsewhere is also revealed by lesion studies in animals, and in patients. For example, the neonatal ventral hippocampal lesion rat, a model for neurodevelopmental aspects of schizophrenia, shows altered responses to sound in inferior colliculus and auditory cortex compared to controls, such as reduced power of the 40-Hz auditory steady state response (ASSR) (Li et al., 2018, Macedo et al., 2010, Vohs et al., 2012, Vohs et al., 2010, Vohs et al., 2009). ASSRs are abnormally lateralized in in medial temporal lobe epilepsy patients (Matsubara et al., 2018, Shigeto, 2021), who also have reduced magnetic evoked responses to pure tones in auditory cortex ipsilateral to the hippocampal sclerosis (Chatani et al., 2016, Matsubara et al., 2018). Finally, pharmacological and chemogenetic shutdown of projections from dorsal CA1 via medial entorhinal cortex affects the amplitude and latency of the mismatch negativity response in mouse auditory cortex (Yi et al., 2022). See Supplementary Tables A and B for other relevant physiological studies.

In sum, multiple pathways are available for auditory information to reach the hippocampus, and for the hippocampus in turn to influence activity at canonical auditory structures. Although the most direct have so far only been anatomically verified in rodents, some electrophysiological studies hint at their presence in humans.

## 3 Sound responses in the absence of a task

**Fig. 3:**
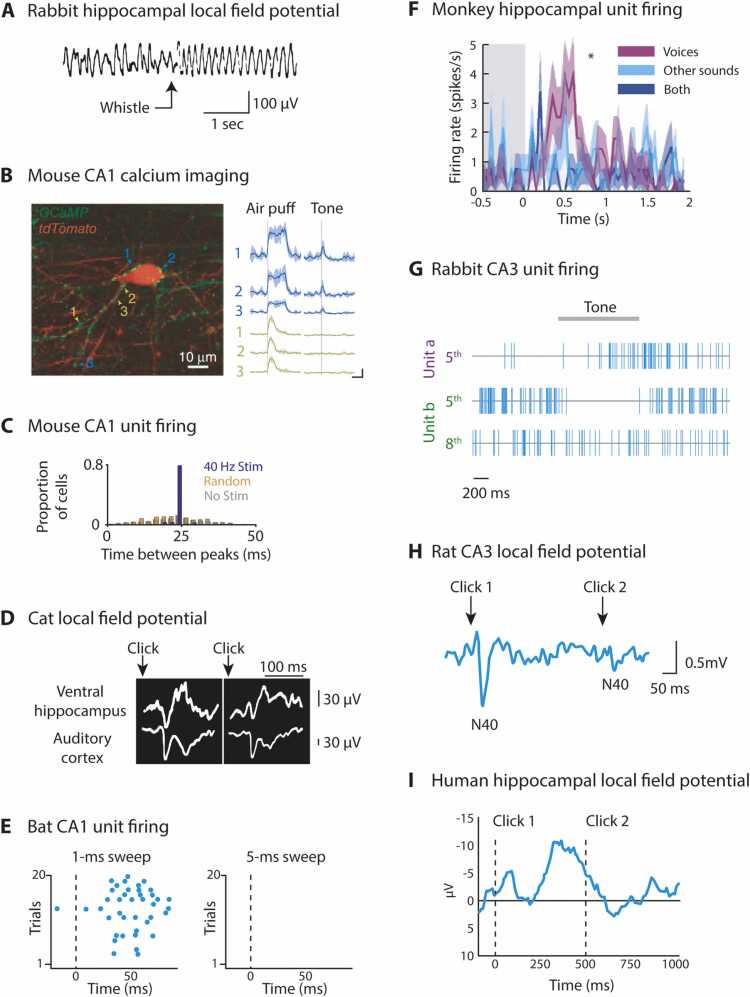
Selection of hippocampal responses to sound in the absence of a task. (A) Bilateral LFP responses in rabbit hippocampus to a whistle. Adapted from Green, J.D., Arduini, A.A., 1954. Hippocampal electrical activity in arousal. J. Neurophysiol. 17, 533–557. Green and Arduini (1954). (B) *Left*: Postsynaptic CA1 interneurons expressing *tdTomato* (red) and septo-hippocampal GABA axons expressing *GCaMP5* (green) in mouse hippocampus with six labelled boutons. *Right*: Stimulus-triggered Ca^2+^ averages (+/- SEM) at the same six boutons in response to air-puffs or a 20-s 10-kHz tone. Scale bars show 50% Δ*F*/*F* and 3 s. Adapted by permission from Springer Nature Customer Service Centre GmbH: Springer. Nature Neuroscience. Septo-hippocampal GABAergic signaling across multiple modalities in awake mice. Kaifosh, P., Lovett-Barron, M., Turi, G.F., Reardon, T.R., Losonczy, A., 2013. Nat. Neurosci. 16, 1182-1184. Copyright © 2013 Nature America, Inc. Kaifosh et al. (2013). (C) Intervals between firing rate peaks in 338 CA1 cells in 5 mice during 40 Hz click stimulation (blue), random-interval click stimulation (orange) and no stimulation (gray). Reprinted from Cell, 177, Martorell, A.J., Paulson, A.L., Suk, H.-J., Abdurrob, F., Drummond, G.T., Guan, W., Young, J.Z., Kim, D.N.-W., Kritskiy, O., Barker, S.J., Mangena, V., Prince, S.M., Brown, E.N., Chung, K., Boyden, E.S., Singer, A.C., Tsai, L.-H, Multi-sensory gamma stimulation ameliorates Alzheimer’s-associated pathology and improves cognition, 256-271, Copyright © 2019 Elsevier Inc., with permission from Elsevier. Martorell et al. (2019). (D) Similar-latency LFP responses in cat ventral hippocampus and auditory cortex. Responses to two successive clicks separated by 15 s are shown side by side. Reprinted from Electroencephalography and Clinical Neurophysiology, 8, Green, J.D., Adey, W.R., Electrophysiological studies of hippocampal connections and excitability, 245-262, Copyright © 1956 Published by Elsevier Ireland Ltd., with permission from Elsevier. Green and Adey (1956). (E) Spike rasters for a single cell in bat CA1 show selective responses to frequency sweeps of 1-ms duration (left) but not 5-ms duration (right) duration presented at 0 ms. Adapted with permission from Yu, C., Moss, C.F., 2022. Natural acoustic stimuli evoke selective responses in the hippocampus of passive listening bats. Hippocampus 32, 298-309. Copyright © 2022 The Authors. Hippocampus published by Wiley Periodicals LLC. Yu and Moss (2022). (F) Mean firing rate (+/- SEM) of single cell in monkey hippocampus in response to voices and other sounds. Adapted from Sliwa, J., Planté, A., Duhamel, J.-R., Wirth, S. Independent neuronal representation of facial and vocal identity in the monkey hippocampus and inferotemporal cortex. Cerebral Cortex, 2014, 26, 950-966, by permission of Oxford University Press. Sliwa et al. (2014). (G) Spike trains of rabbit CA3 neurons. *Top*: Activatory response at Unit A to 5th presentation of a 900 Hz tone. *Middle/Bottom*: Responses at Unit B to 5th and 8th presentations of an 800 Hz tone, showing suppression that habituates over trials. After Vinogradova (1975a). (H) Grand average evoked LFP response from CA3 in 12 rat hippocampi to pairs of clicks presented 500 ms apart. Reprinted from Biological Psychiatry, 27, Bickford-Wimer, P.C., Nagomoto, H., Johnson, R., Adler, L.E., Egan, M., Rose, G.M., Freedman, R., Auditory sensory gating in hippocampal neurons: A model system in the rat, 183-192, Copyright © 1990 Published by Elsevier Inc., with permission from Elsevier. Bickford-Wimer et al. (1990). (I) Grand average evoked response recorded intracranially in 21 human posterior hippocampi to clicks presented at 0 and 500 ms (dashed lines). Adapted with permission from Boutros, N.N., Mears, R., Pflieger, M.E., Moxon, K.A., Ludowig, E., Rosburg, T. Sensory gating in the human hippocampal and rhinal regions: Regional differences. Hippocampus 18, 310-316. Copyright © 2007 Wiley-Liss, Inc. Boutros et al. (2008).

Sounds that hold no meaning for a passively listening animal elicit a number of forms of hippocampal response (see Fig. 3 for a selection and Supplementary Table C for a more complete list). The prominent theta component of hippocampal electroencephalographic (EEG) activity has been variously associated with exploratory movement, memory encoding and retrieval, and arousal, but since the earliest hippocampal recordings in anaesthetized rabbits and cats (Green and Arduini, 1954, Jung and Kornmüller, 1938) as well as in awake animals (Eidelberg et al., 1959, Irmiš et al., 1970), increases in theta power have also been observed in response to meaningless stimuli such as clicks (Fig. 3A). Pure tone presentation can also reset the phase of ongoing hippocampal oscillations (Başar et al., 1979a, Başar et al., 1979b, Başar and Demiralp, 1995, Demiralp et al., 1996, Abe et al., 2014a) in the absence of any task. Such resets may contribute to deflections in evoked hippocampal local field potentials that have been described in anesthetized or passively listening animals (Başar and Özesmi, 1972, Başar and Ungan, 1973, Brankačk and Buzsáki, 1986, Green and Adey, 1956, Hall and Borbely, 1970, Liberson and Cadilhac, 1953, O’Connor et al., 1992, Ungiadze, 1967) and humans (Rosburg et al., 2007).

Different classes of hippocampal cells can be distinguished based on anatomy and physiology, including the relationship of their firing to ongoing hippocampal theta oscillations (Ranck, 1973). Inhibitory interneurons tend to fire with a consistent theta phase at a rate that can increase or decrease in response to meaningless sound (Miller and Freedman, 1995, Vinogradova, 2001). Such responses have been imaged at the level of individual synaptic boutons in mouse CA1 that receive GABAergic projections from the septum (Kaifosh et al., 2013; Fig. 3B). In contrast, principal pyramidal cells show occasional burst firing with no fixed relationship to theta phase. Although their most famous behavioral correlate is physical location (“place cells”, O’Keefe and Dostrovsky, 1971; see Section 8), hippocampal pyramidal cells also respond to pure tones, artificial vowels, and noise, even in the absence of a task (Miller and Freedman, 1995, Vinnik et al., 2012).

Hippocampal responses to sound can be brief, persist throughout or beyond the stimulus, or be phasically modulated by its temporal structure (Lidsky et al., 1974, Martorell et al., 2019; Fig. 3C). Although responses in hippocampus typically follow in auditory cortex, this is not always the case (Green and Adey, 1956; L. Zhang et al., 2019; Fig. 3D). This indicates that subcortical hippocampal afferents described earlier may convey the presence of sound, a proposition further supported by the fact that responses of some medial septal neurons to behaviorally irrelevant sounds precede periods of elevated hippocampal and theta and gamma power (Zhang et al., 2011) and that medial septal inactivation reduces auditory responses in certain hippocampal subfields (Xiao et al., 2018). The medial septal pathway may be particularly responsive to high-intensity sounds in mice (Abe et al., 2014b, Kaifosh et al., 2013). Given that temporal windows of integration increase along the lemniscal pathway to primary auditory cortex and thence through the cortical hierarchy (Baumann et al., 2015, Dheerendra et al., 2021, Joris et al., 2004, Nourski et al., 2009), the alternate subcortical routes may also be those that carry rapid temporal modulations to hippocampus (Arnal et al., 2019, Chan et al., 2021, Martorell et al., 2019).

Many of the hippocampal responses described above, whether at the single cell or evoked potential level, are not specific to particular sounds but rather scale with intensity. Because the hippocampus is involved in a range of cognitive and spatial processing, such unselective responses are hard to attribute to auditory processing per se rather than to general arousal or orienting. However, there are exceptions to this lack of selectivity during passive exposure: Brown and Buchwald (1973) describe stable hippocampal tuning to tone frequency in cats, Yu and Moss (2022; Fig. 3E) report duration tuning in bat CA1, and Sliwa et al. (2014; Fig. 3F) find voice-selective responses in the hippocampus of monkeys - in all cases the animals were listening passively. Most single cell and evoked potential recordings during passive listening will have been insensitive to any fine-grained population coding. Calcium imaging allowing simultaneous mapping of activity of large numbers of neurons has identified robust and stable but sparse responses to passively presented odors in mouse dentate gyrus (Woods et al., 2020), but has rarely been used to detect auditory hippocampal responses.

A key factor influencing the magnitude of hippocampal responses to sound in the absence of behavior is stimulus history. Although habituation to a repeated auditory stimulus is a widespread neural phenomenon (Bickford et al., 1993, Miller and Freedman, 1993, Moxon et al., 1999, Picton et al., 1976), it is particularly pronounced in hippocampus, at least for behaviorally irrelevant input in rodents and rabbits (Bickford-Wimer et al., 1990, Vinogradova, 1975b). Both firing rate changes (Vinogradova et al., 1970; Fig. 3G; Vinogradova, 1975b, Vinogradova, 2001) and evoked responses (Bickford-Wimer et al., 1990, Ehlers et al., 1994, Kaneko et al., 1993, Ruusuvirta et al., 1996; Fig. 3H) reduce in magnitude with each repetition. In these studies, a striking reduction in sound responses occurs after only one sound. Many rodent studies have focused on the neurochemical basis of the reduced hippocampal response to the second in a pair of stimuli separated by a 500-ms interval, because impairment of such “sensory gating” at the scalp is associated with various human psychiatric disorders (for a review see Cromwell et al., 2008). An intact hippocampus is also important for pre-pulse inhibition of the acoustic startle response - an important behavioral correlate of sensory gating (Inta et al., 2014, Kemble and Ison, 1971; see also Supplementary Table H). But habituation effects can also extend for much longer than the intervals in these gating studies – for several seconds in rats (Mays and Best, 1975) or even minutes in rabbits (Vinogradova et al., 1970).

Selective habituation to identical stimuli gives rise to a neural indicator of the presence of novelty. In many species, any perceptible change in stimulus after repetition can be sufficient to restore a large hippocampal response, with a greater effect for larger differences and more rarely occurring sounds (Başar-Eroglu et al., 1991, Csépe et al., 1989, Ruusuvirta et al., 2013, Vinogradova, 2001). Although differences between passively heard repetitive auditory standards and deviants have been detected in human hippocampus (Fuhrer et al., 2021, Herdener et al., 2010, Rosburg et al., 2007, Zevin and McCandliss, 2005; Fig. 3I) these diminish over the course of an experimental session, possibly reflecting that at the level of hippocampal processing deviant sounds become less unexpected (Rosburg et al., 2007). Gross hippocampal novelty responses during passive listening are unlikely to directly contribute to the human mismatch negativity response recorded at the scalp, which has predominantly superior temporal and inferior frontal generators (Näätänen, 1990). However, the amplitude and latency of the mismatch negativity in mouse auditory cortex have recently been found to depend on a circuit via entorhinal cortex and the hippocampal trisynaptic loop (Yi et al., 2022). In the context of vision, Kumaran and Maguire (2007) identified that the novelty not of an individual stimulus but rather of associations between stimuli is particularly important in driving hippocampal activity. We will come to the role of hippocampus in representing or forming associations between sounds and other sounds, images, and locations in subsequent sections. But first we consider how this structure is involved when animals learn to associate a sound with value in appetitive or aversive conditioning.

## 4 Activity during conditioning to sound

Training animals to associate a particular sound (conditioned stimulus) with a subsequent reward or punishment (unconditioned stimulus) elicits changes in hippocampal activity over the course of learning (see Supplementary Table D). Different hippocampal synapses are modified in strength through long term potentiation (for a review see Gruart et al., 2015), leading to changes in firing rates that differentiate sounds that have been associated with value from those that have not (Berger et al., 1976, Disterhoft and Segal, 1978, Klee et al., 2021, Olds and Hirano, 1969)*.* Such differential responses can arise between tones of different frequencies, or between more complex sounds such as artificial vowels with different formant structure (Itskov et al., 2012). Conditioning correlates are observed not only in firing rates of hippocampal cells, but also in local field potentials. For example, in rat dentate gyrus the amplitude of a late sustained component increases as the animal learns the association between a tone and water reward (Deadwyler et al., 1985). This contrasts with an earlier component, the magnitude of which depends on the identity of multiple preceding stimuli (whether reinforced or not), consistent with habituation effects described in Section 3. Theta activity also accompanies different phases of learning (Adey et al., 1960, Berry and Seager, 2001, Grastyán et al., 1959, Hoffmann et al., 2015) and can itself affect the success of conditioning (Berry and Seager, 2001, Hoffmann et al., 2015).

The temporal pattern of firing that develops during conditioning may reveal something of what is learned. During eyeblink conditioning, animals learn to associate a tone with a subsequent air-puff (Berger et al., 1976). As the animal begins to acquire the conditioned response – blinking just prior to the air-puff – the timecourse of hippocampal pyramidal cell firing comes to resemble the motor response, occurring progressively earlier than it as conditioning progresses (Berger et al., 1980). However, hippocampal responses and motor behavior do not always correspond; during extinction or learning of new associations, firing patterns in some units may reflect stimulus contingencies that are not shown in behavior (Berger and Thompson, 1982, Hoehler and Thompson, 1979, Laroche et al., 1987). Nor does the appearance of hippocampal firing require there to be an overt conditioned movement (Hirano and Yamaguchi, 1985).

Although most hippocampal recordings during conditioning with auditory stimuli have been made in rats and rabbits, some of the principal findings have been replicated in cats (Patterson et al., 1977). Humans show increased metabolic and hemodynamic activity in regions including hippocampus during eyeblink conditioning compared to when sounds and air-puffs are presented unpaired – this activity correlates weakly with learning (Blaxton et al., 1996, Cheng et al., 2008, Logan and Grafton, 1995).

Despite these extensive learning-related changes in neural activity, animals with hippocampal damage are not impaired in many conditioning settings (Berger and Orr, 1983, Berger and Orr, 1982, Brady et al., 1954, Brady and Hunt, 1955, Ross et al., 1984, Schwartzbaum et al., 1964; see Supplementary Table H). Multiple brain areas are involved during learning, and when a conditioned stimulus is sufficiently loud (Wu et al., 2013) and the unconditioned stimulus sufficiently close in time (Beylin et al., 2001), the essential neural circuitry lies elsewhere. For example the cerebellum is critical for eye-blink conditioning (Daum et al., 1993, McCormick et al., 1982, Thompson, 2005), the amygdala for fear conditioning (Phillips and LeDoux, 1994), and the striatum for appetitive conditioning (Cole et al., 2017). An intact hippocampus appears most important when there is a silent interval to be bridged between conditioned and unconditioned stimulus (Clark and Squire, 1998, Solomon et al., 1986; see Supplementary Table H), as well as when conditioned responses are to be constrained, such as by spatial context or configurations of cues - especially if such configurations are to be learned incidentally and rapidly (Rudy, 2009). Subsequent sections build on some of these points to detail the role of hippocampus in temporal, sequential and spatial aspects of auditory processing. First, we consider some further aspects of hippocampal responses to sounds that have been associated with value or become pertinent to a task.

## 5 Activity during task-based listening

Once sounds have acquired behavioral relevance, hippocampal activity can be examined as animals perform tasks relating to them (see Supplementary Table E). For example, in rodents trained to press a lever when they detect rare frequency deviants in a train of standard sounds, late (250–500 ms post onset) differences in local field potentials to targets versus standards are particularly pronounced (Brankačk et al., 1996, Ehlers et al., 1994, Hattori et al., 2010, Shin, 2011, Shinba, 1999, Shinba et al., 1996). Other signatures of target detection include induced theta and gamma power increases (Shin, 2011) as well as more complex firing patterns in pyramidal cells than arise during standard tones (Gao et al., 2010). In one study, late firing increases occurred for targets but not standards, regardless of target intensity. This was in contrast to an earlier (~40 ms) peak that occurred for all tones and scaled with intensity (Shinba, 1999) and the dominance of stimulus intensity as a determinant of firing rate in the passive listening studies described earlier. For some units in the study by Shinba (1999), the degree of late firing activity correlated with both the amplitude of the late LFP response and how quickly behavioral responses were made.

**Fig. 4:**
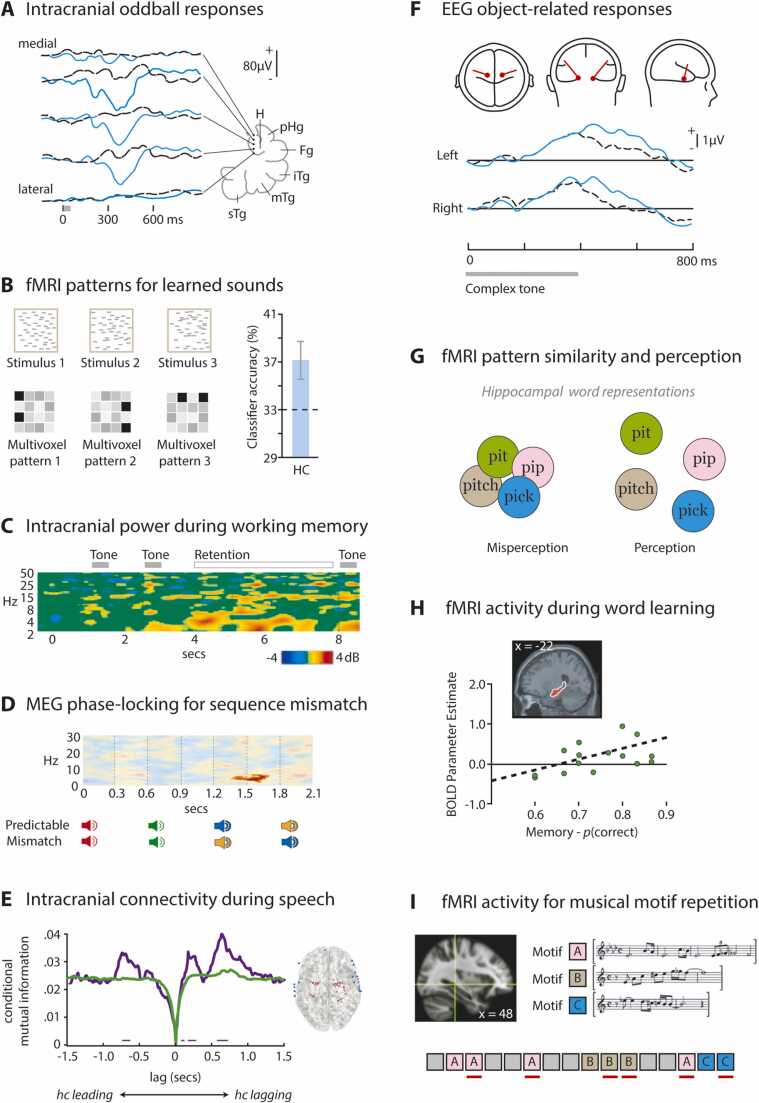
Selection of human studies with attentive or task-based listening. (A) *Left*: Intracranial potentials elicited by frequent (dashed black traces) and rare (solid blue traces) pure tones of different frequencies in medial temporal lobe of a single patient. *Right*: Electrode locations indicated on line drawing of brain, lateral surface at bottom. H=hippocampus, pHg=parahippocampal gyrus, Fg=fusiform gyrus, iTg=inferior temporal gyrus, mTg=medial temporal gyrus, sTg=superior temporal gyrus. Reprinted from Electroencephalography and Clinical Neurophysiology, 76, Smith, M.E., Halgren, E., Sokolik, M., Baudena, P., Musolino, A., Liegeois-Chauvel, C., Chauvel, P., The intracranial topography of the P3 event-related potential elicited during auditory oddball, 235-248, Copyright © 1990 Elsevier Scientific Publishers Ireland, Ltd, with permission from Elsevier. Smith et al. (1990). (B) *Top*: Cartoon time-frequency spectrograms of three 1.5-s complex noise stimuli consisting of overlapping pure-tone pips. *Bottom:* Cartoon hippocampal multivoxel BOLD activity patterns elicited by each of these stimuli after implicit learning through repeated exposure in a stream of other noise stimuli. *Right:* Above-chance decoding of the same stimuli from the multivoxel patterns in 7 subjects (mean and standard error). Adapted from Fig. 4 in Kumar S., Bonnici H.M., Teki, S., Agus, T.R., Pressnitzer, D., Maguire, E.A., Griffiths, T.D., 2014. Representations of specific acoustic patterns in the auditory cortex and hippocampus. Proc. R. Soc. B 281: 20141000. Licensed under CC-BY. Kumar et al. (2014). (C) Event-related spectral perturbation at a hippocampal electrode during a working memory task. Subjects heard tones of two frequencies (first two gray lines) then received a retro-cue advising which to hold in mind over a delay period until comparing to a target tone (third gray line). A pronounced increase in delta-theta power is apparent during the delay period compared to a pre-trial baseline. Adapted from Fig. 3 in Kumar S., Gander, P.E., Berger, J.I., Billig, A.J., Nourski, K.V., Oya, H., Kawasaki, H., Howard, M.A., Griffiths, T.D., 2021. Oscillatory correlates of auditory working memory examined with human electrocorticography. Neuropsychologia, 150. Licensed under CC-BY. Kumar et al. (2021). (D) Source-localized hippocampal inter-trial phase coherence of MEG recordings during implicit comparison of pure tone sequences. Saturated red region reflects significantly greater theta coherence when frequencies of third and fourth tones in the sequence mismatch implicit predictions compared to when they match. Adapted from Figs. 1, 3 in Recasens, M., Gross, J., Uhlhaas, P.J., 2018. Low-frequency oscillatory correlates of auditory predictive processing in cortical-subcortical networks: A MEG-study. Sci. Rep., 8, 14007. Licensed under CC-BY. Recasens et al. (2018). (E) Nine participants heard two repetitions of a story. Intracranial electrode sites were identified where 70–200 Hz activity showed signs of predictive recall during the second repetition; these included auditory cortex. *Left:* Connectivity was assessed between these sites and either hippocampus (purple trace) or all other sites (green trace) at moments of peak predictive recall. Mutual information in the neural time series (y-axis) is shown at different lags (x-axis) (excluding influences at zero lag). Purple horizontal bars indicate lags for which mutual information between hippocampus and predictive recall sites was significantly greater than chance. Left-most bar and peak indicates information flow from hippocampus to sites including auditory cortex 720 ms prior to predictive recall. *Right:* Ventral view of brain showing hippocampal electrode sites (red) and neocortical predictive recall sites (blue) included in the analysis. Adapted from Figs. 3, 5 in Michelmann, S., Price, A.R., Aubrey, B., Strauss, C.K., Doyle, W.K., Friedman, D., Dugan, P.C., Devinsky, O., Devore, S., Flinker, A., Hasson, U., Norman, K.A., 2021. Moment-by-moment tracking of naturalistic learning and its underlying hippocampo-cortical interactions. Nat Commun. 12, 5394. Licensed under CC-BY. Michelmann et al. (2021). (F) Subjects listened to a complex tone, which sometimes contained a mistuned harmonic, and reported whether they heard one or two sounds. *Top*: Location (red dots) and orientation (red lines) of pair of equivalent current dipoles in medial temporal lobes contributing to EEG scalp activity during the task. *Bottom*: Activity projected to left and right hemisphere dipoles when the complex tone (gray bar) did (solid blue traces) or did not (dashed black traces) contain a mistuned harmonic. From Alain, C., Arnott, S. R., & Picton, T. W. (2001). Bottom-up and top-down influences on auditory scene analysis: Evidence from event-related brain potentials. *Journal of Experimental Psychology: Human Perception and Performance, 27*(5), 1072-1089. Copyright © 2001 American Psychological Association. Reproduced and adapted with permission. Alain et al. (2001). (G) Cartoon of hippocampal representations of candidate words during a degraded speech task. Closer circles reflect more overlapping representations, as assessed by similarity of multivoxel BOLD activity patterns. When 24 participants heard a degraded word preceded by a partially mismatching visual cue, those whose hippocampal representations of mismatching candidate words were more distinct were more likely to perceive the correct spoken word. Based on results from Blank et al. (2018). (H) Univariate BOLD activity in anterior and middle hippocampus (*top*) during exposure to novel pseudo-words correlates positively across 16 participants with their ability to subsequently recognize the stimuli (*bottom*). Copyright © 2008 Massachusetts Institute of Technology. Adapted with permission. Davis, M.H., Di Betta., A.M., Macdonald, M.J.E., Gaskell, M.G., 2009. Learning and consolidation of novel spoken words. J. Cogn. Neurosci. 21, 803-820. Davis et al. (2009). (I) 11 participants listened to a live recording of a multi-instrumental piece of tango music, containing a number of repeating motifs (*right*). After key acoustic features were modelled out from the univariate BOLD signal, repetitions (but not first occurrences) of the motifs activated regions including hippocampus (*left*). *Bottom*: Indicative illustration of motifs with repeats underlined in red, and intervening non-motivic material in gray. Reprinted from Cortex, 57, Burunat, I., Alluri, V., Toiviainen, P., Numminen, J., Brattico, E. Dynamics of brain activity underlying working memory for music in a naturalistic condition, 254-269, Copyright © 2014 Elsevier Ltd., with permission from Elsevier. Burunat et al. (2014).

In human subjects, behavioral relevance can be instilled in the absence of explicit reward through task instructions, such as to count particular target sounds or to make certain judgments. By having subjects report target counts at the end of a trial or block, explicit motor confounds are removed. In contrast to the passive listening case in humans described earlier, active detection of rare frequency (Altafullah et al., 1986, Halgren et al., 1995, Halgren et al., 1980, Kropotov et al., 2000, Kropotov et al., 1995, McCarthy et al., 1989, Smith et al., 1990, Smith et al., 1986, Stapleton et al., 1987) or intensity (Velasco et al., 1986) targets generates large hippocampal LFP deflections with peaks between 260 and 500 ms (Fig. 4A). These are also accompanied by changes in local unit activity (Halgren et al., 1980, Heit et al., 1990) and may contribute to the P3/P300 scalp component (Fonken et al., 2019, Sutton et al., 1965). Some fMRI studies also show increased hippocampal BOLD for rare target sounds (Crottaz-Herbette et al., 2005, Yoshiura et al., 1999) or when listening to changes in pitch rather than identifying particular pitches (Schwenzer and Mathiak, 2011).

Even if a subject is not aware of what distinguishes a particular sound from others, previous exposure can result in the formation of distinct hippocampal representations that are revealed during subsequent active listening. For example, Kumar et al. (2014) found exemplar-specific patterns of multivoxel hippocampal BOLD activity for complex noise stimuli during a repetition detection task (Fig. 4B). Although subjects had been exposed to these specific exemplars multiple times during an earlier training session, they did not typically recognize their recurrence across the experiment. It may be that some degree of pre-exposure is important for hippocampal representations of sounds to form: in another study with no pre-exposure, Liang et al. (2013) were unable to decode particular environmental sounds or spoken words from multivoxel patterns in hippocampus during target detection, even though these stimulus classes could be distinguished from others (such as faces and visual words) in such patterns. Further cases of hippocampal responses during actively attended sounds will be discussed in later sections on space, sequences, and the special cases of speech and music. But first we consider hippocampal involvement in temporal aspects of sound, including during the silence between a behaviorally relevant sound and the reward, punishment, or task that follows.

## 6 Time and working memory for sound

As mentioned earlier, whereas animals with hippocampal lesions are able to learn the association between a conditioned stimulus and unconditioned stimulus if these are presented in an overlapping or abutting fashion (“delay conditioning”), the insertion of a silent interval between the two (“trace conditioning”) renders these animals impaired. In rodents, persistent firing of individual hippocampal neurons over the trace interval is rare (Gilmartin and McEchron, 2005, McEchron et al., 2003, McEchron and Disterhoft, 1997, Weiss et al., 1996). However, calcium imaging reveals subsets of mouse CA1 neurons that together selectively encode the conditioned stimulus identity as they span this interval (Ahmed et al., 2020, Modi et al., 2014).

This bridging of a silent interval by assemblies of hippocampal cells, each with its own temporal firing field, is not limited to conditioning paradigms in which a sound is to be associated with a subsequent appetitive or aversive stimulus. Hippocampal “time cells” (Manns et al., 2007, Pastalkova et al., 2008) are also active when animals have to retain stimulus-specific information in memory over a short silent interval, for example to compare an odor to a probe in a delayed-match-to-sample task (MacDonald et al., 2013). The population of cells encodes the retained stimulus with a fidelity that predicts subsequent task performance. Time cells have also been identified in human hippocampus during a free recall task (Umbach et al., 2020) as have cells selective for particular visual stimuli that fire at fixed phases of low-frequency (1–7 Hz) oscillations when they are held in mind over a delay period (Kamiński et al., 2017, Kornblith et al., 2017). Comparable findings linking unit activity to oscillatory phase or particular timepoints are yet to be reported for auditory memoranda, however the degree of synchrony among groups of neurons in rat CA1 can distinguish a tone frequency held in memory from another, even when such information is not carried in the firing rate of individual neurons (Takahashi and Sakurai, 2009). In humans, increases in hippocampal BOLD activity (Kumar et al., 2016) and low frequency oscillatory power (Kumar et al., 2021) emerge when human subjects keep a tone frequency in mind for comparison to a probe (Fig. 4C). It has been argued that the hippocampus is involved in retention over a few seconds only when the stimuli require complex high-resolution binding (Yonelinas, 2013) or additional demands are placed on working memory (Jeneson and Squire, 2011), but neither were the case in the Kumar et al. (2021) study.

Does the presence of activity in these cases reflect a *critical* role for hippocampus in maintaining sound features over a temporal interval, outside of a conditioning setting? Lesioning the fimbria-fornix input to rat hippocampus impairs their ability to remember the presentation rate of click trains, as well as their duration (which is consistently underestimated; Meck et al., 1984). Medial temporal lesions in monkeys impair short-term retention of sound identity although it has been argued that this is an artifact of damage during surgery to auditory-prefrontal cortical pathways (Fritz et al., 2005). Dogs with medial temporal lobe lesions can retain sound identities for over a minute (Kowalska et al., 2001), although their memory for tone locations over a 10-s delay is impaired (Kowalska, 1999). Human patients with hippocampal damage can struggle to hold sounds in mind for several seconds, at least if the material cannot be rehearsed sub-vocally (Cave and Squire, 1992, Chao and Knight, 1995, Keane et al., 1995, Milner, 1972, Milner and Teuber, 1968, Penfield and Milner, 1958, Squire et al., 2001, Stefanacci et al., 2000, Wickelgren, 1968). It seems that the criticality of the hippocampus depends on species and the auditory feature to be maintained (see Supplementary Tables H, I).

The relative contributions of prefrontal cortex and hippocampus to working memory remain a matter of debate (Jin and Maren, 2015, Sreenivasan and D’Esposito, 2019, Tang et al., 2021). Prefrontal cortex receives input from auditory cortex (Plakke and Romanski, 2014, Rocchi et al., 2021, Romanski et al., 1999) and activity there can encode the frequency of a tone held in mind (Kumar et al., 2016). Lesioning or disrupting medial prefrontal cortex in rats (Rodgers and DeWeese, 2014) and lateral prefrontal cortex in non-human primates (Gross and Weiskrantz, 1962, Plakke et al., 2015) impairs auditory working memory, suggesting a critical role. Hippocampus and prefrontal cortex, which are connected via direct and indirect pathways (for a review, see Eichenbaum, 2017a), may work together to support short-term maintenance of auditory material, but such interactions have not yet been directly tested.

The involvement of hippocampus in temporal processing - beyond bridging silent gaps described above - has been extensively reviewed by Banquet et al. (2021). With respect to sound, neurons in rodent hippocampus can be tuned to specific durations (e.g. 2 vs. 8 s) when these are relevant to auditory behavior (McEchron et al., 2003, Onoda et al., 2003). However, duration may be subordinate to pitch in hippocampal coding according to results from Sakurai (2002), who trained rats to perform both pitch and duration discrimination on the same stimulus set. Some hippocampal neurons were tuned to pitch alone and others to both pitch and duration, but none to duration alone. In contrast to the task-based findings in rodents, some neurons in bat CA1 are tuned to the duration (e.g. 1 vs 3 ms) of frequency sweeps, even during passive listening (Yu and Moss, 2022). As these resemble calls used for echolocation, it might be argued that such tuning relates to hippocampal spatial function (see also Section 8). However, no tuning was found in the same study to the delay between call and echo, a more relevant feature for navigation.

Longer time intervals related to auditory content can also be read out from hippocampus. The similarity of hippocampal multivoxel BOLD patterns associated with clips from different times in a spoken narrative is correlated with the perceived elapsed time between the clips (Lositsky et al., 2016). This is consistent with the idea that the medial temporal lobe provides a slowly shifting mental context that acts as a temporal tag for memories of items and episodes (Howard et al., 2005, Yonelinas et al., 2019; see also Section 13). At the other end of the temporal scale, while processing of sub-second intervals between sounds draws largely on extra-hippocampal structures, such as cerebellum and striatum (Nani et al., 2019, Teki and Griffiths, 2016), there is some indirect evidence for hippocampal involvement at these shorter timeframes (see Supplementary Table I). Patients with medial temporal lobe epilepsy including hippocampal sclerosis have problems identifying patterns of durations of hundreds of milliseconds (Han et al., 2011), making anisochrony judgments on the order of tens of milliseconds (Lavasani et al., 2013), and detecting gaps in noise at below 10 ms (Ehrlé, 2001, Rabelo et al., 2015). Replicating these findings in other groups with circumscribed hippocampal damage will be valuable. As we shall see in Section 9, the hippocampus may be critically important when it comes to storing the *order* of stimuli in time.

## 7 Sound context

The hippocampus plays an important role in learning the constraints and contexts under which reward contingencies and behaviors should apply (see Supplementary Table H). Animals with hippocampal lesions fail to inhibit responses to uninformative or salient but unconditioned sounds (Freeman et al., 1973, Loechner and Weisz, 1987, Micco and Schwartz, 1971, Niki, 1967, Rickert et al., 1979, Rickert et al., 1978, Solomon, 1977, Solomon and Moore, 1975, Swanson and Isaacson, 1967) and take much longer to learn to extinguish a conditioned response to sound than control animals (Berger and Orr, 1983, Berger and Orr, 1982, Schmaltz and Theios, 1972). Neocortical sites including perirhinal cortex can also support the learning of conditional dependencies, but only with sufficient exposure and when those associations are important for current behavior. The hippocampus learns more rapidly – even after a single exposure - and importantly can associate stable background elements that occur together into a context, even if they are not associated with reward or punishment at the time (Rudy, 2009, Rudy and O’Reilly, 2001). Once a context representation has been formed, pattern completion mechanisms supported by auto-associative networks in CA3 may allow a full context to be retrieved from a partial cue (Marr, 1971, McNaughton and Morris, 1987, O’Reilly and McClelland, 1994). The neocortical and hippocampal learning systems likely operate in parallel (Hebscher et al., 2019, McClelland and O’Reilly, 1995), but the dominance of the hippocampal system is illustrated by impaired context retrieval when hippocampal lesions are made after but not before learning (Lehmann et al., 2009). A broader definition of context is adopted in the contextual binding theory of human episodic memory (Yonelinas et al., 2019; see also Section 13). It holds that not only background elements of the physical environment, but also slowly changing cognitive state or mood as well as temporal context, are bound together in the hippocampus with items and events as they are encountered or experienced. The theory accounts for a range of experimental data, including interference in memory between items encountered in similar places or times, or under similar behavioral states.

The dependence of context learning on hippocampus matters for sound processing in a number of ways. First, hippocampally lesioned rats fail to show context-specific expression or extinction of auditory fear conditioning - instead the conditioned stimulus can trigger the fear behavior even in inappropriate contexts (Corcoran and Maren, 2001, Holt and Maren, 1999, Hunsaker and Kesner, 2008). Second, a context may itself include auditory elements. For example, Grau-Perales et al. (2019) investigated the role of hippocampus in contextual control of habituation of taste neophobia, whereby mice are initially reluctant to drink novel-tasting solutions but become less averse over days. Whereas a change in the auditory context (the presence of a pure tone versus white noise) resets this attenuation in control animals, this is not the case for lesioned animals. Sounds forming part of a context can also be presented as a partial cue to reactivate hippocampus-dependent memories. In rats, such reactivation has allowed the ensemble of hippocampal cells encoding a specific memory to be reactivated and subsequently targeted through inhibition of protein synthesis (Ressler et al., 2021). Interfering with memory reconsolidation in this way may be relevant for future clinical interventions that rely on indirect retrieval of traumatic memories.

Whether the hippocampus is involved in combining purely auditory cues into a context has not been investigated. It would be informative to establish whether hippocampally lesioned animals can automatically form a context representation on the basis of - for example - the level of reverberation and type of background noise in an environment, to constrain conditioned behavior.

## 8 Interactions between sound and space

The automatic learning of associations in the absence of reward, highlighted with respect to context learning in the previous section, is at the heart of the “cognitive map” concept originally proposed by Tolman (1948). He observed that rats learn the structure of a maze - subsequently enabling them to retrieve alternative routes - even when such (“latent”) learning is not driven by immediate reward. Hippocampal cells that are selectively active during the exploration of space (“place cells”) discovered by O’Keefe and Dostrovsky (1971) offered a biological basis for such a map. We outline a few key features of place cells before discussing their interaction with sound.

Place cells with adjoining place fields fire in sequence as an animal traverses an environment. The formation of place fields is driven by path integration, a computation that transforms motion into a sense of location, supplemented with landmark perception (Savelli and Knierim, 2019). These processes depend on cells that track head direction, speed, boundaries and other environmental properties (Lever et al., 2009, O’Keefe and Burgess, 1996, Taube et al., 1990). Place cells can remap – that is change their firing rate or even firing field - in the presence of changes of context – whether physical, such as a new cage, or task-related (Anderson and Jeffery, 2003, Jackson and Redish, 2007, Leutgeb et al., 2004, Wills et al., 2005). “Grid cells” in entorhinal cortex fire at multiple locations arranged in a hexagonal grid, tiling the environment between them (Hafting et al., 2005). Grid spacing increases from dorsal/posterior to ventral/anterior entorhinal cortex (Brun et al., 2008) but generally remains fixed across environments (Fyhn et al., 2007); these properties enable grid cells to provide a stable coordinate and metric system. There is a systematic change in the phase of theta oscillations at which a place cell fires as the animal moves through its firing field (“phase precession”; O’Keefe and Recce, 1993 in rodents; Qasim et al., 2021 in humans). This results in each theta cycle containing a representation of the recent past (places visited), present (current location) and future (planned trajectory). Such sequences are further compressed during “sharp-wave ripple” events (Buzsáki et al., 1983). In rodents these happen during pauses in navigation, and can be decoded as reflecting both recently experienced ("replay") and future ("preplay") trajectories (Diba and Buzsáki, 2007). They can occur forward or in reverse, may reflect credit assignment and planning (Foster and Wilson, 2006, Pfeiffer and Foster, 2013), and are associated with memory consolidation during sleep (see Section 13; for a general review of sharp-wave ripples see Buzsáki, 2015).

Sound and place cells interact in a variety of ways. Although path integration and use of visual landmarks are key for forming spatial maps in most species, sound provides an important signal for place-cell coding during echolocation in the bat (Ulanovsky and Moss, 2011, Ulanovsky and Moss, 2008, Ulanovsky and Moss, 2007, Yu and Moss, 2022). In rats, changes that induce place cell remapping include auditory fear conditioning in a particular location (Donzis et al., 2013, Moita et al., 2004) and high-intensity sound exposure (Goble et al., 2009). Even the introduction of a behaviorally-irrelevant sound can influence aspects of spatial coding, such as the relationship between locomotion speed and hippocampal theta (Long et al., 2014).

Synaptic change in CA1 facilitates learning the association between locations with behaviorally-salient ultrasonic signals (Dietz and Manahan-Vaughan, 2017), just as with visual cues. Firing of dentate gyrus cell assemblies reflects learning of the mapping between particular tone frequencies and reward location, and silencing these assemblies impairs such learning (J. Shen et al., 2021). In rodents, hippocampus is critical for incidental learning of the association between a sound and its location, and for auditory fear conditioning that is specific to the spatial or spatiotemporal context (Iordanova et al., 2009, Talk et al., 2002). Another case in which the absence of hippocampus prevents incidental learning of associations is provided by Talk et al. (2016). Hippocampally-lesioned rats failed to incidentally learn the association between a noise stimulus and a particular location, demonstrated by their failure to avoid the location in the absence of the sound, after that sound had been paired with a shock. This was despite their learning to associate the sound with the shock, and control animals avoiding the location in the absence of the sound. Memory of auditory space is also impaired in dogs (Kowalska, 1999) and humans (Lancelot et al., 2005, Lancelot et al., 2003) with hippocampal damage (see also Supplementary Tables H, I). In humans, hippocampal lesions - particularly when coupled with superior temporal lobe damage - are associated with under-estimation of the length of sound trajectories in space (Kotelenko et al., 2013). This is somewhat consistent with the attenuation of boundary extension in visual space in patients with hippocampal amnesia (Mullally et al., 2012), and with the underestimation of temporal extent in rats with diminished hippocampal input (Meck et al., 1984).

Space, or spatial context, also has a strong influence over coding of sound in the hippocampus. For example, the firing rate of hippocampal units in response to a conditioned noise stimulus is gated by location-specific tuning (Moita et al., 2003). In another experiment some units in rat CA1 fired selectively in response to one of several rewarded artificial vowel sounds regardless of location, but this mapping only persisted as long as the spatial environment was fixed (Itskov et al., 2012). Some monkey hippocampal neurons are selective for particular types of sound (e.g. human voice over pure tone), but respond only when sounds come from a particular direction, typically behind the animal and out of its visual field (Tamura et al., 1992, Tamura et al., 1990). Eichenbaum and colleagues have demonstrated the mixed selectivity of hippocampal neurons more generally (Eichenbaum, 2017b). Representational similarity analysis over populations of neurons reveals multiplexed coding of context, location, reward, and object – often in that order of precedence (McKenzie et al., 2014). Although position-related firing is normally present from the outset of exposure to a new environment, the extent to which object and reward information are encoded increases based on their relevance to behavior (Lee and Kim, 2010, Muzzio et al., 2009).

## 9 Auditory sequences and predictions

Might the phase precession described in the last section allow for the maintenance in hippocampus of not only spatial trajectories but also auditory sequences? In this way recent, past, current, and predicted or planned sounds (e.g. in a sentence or melody) could be linked based on the phase in a theta cycle at which corresponding neural assemblies fire. Direct experimental support for this kind of auditory phase precession is currently lacking. We know that in their firing *rates* CA1 cells code sequences of non-spatial events, such as the presentation of different odors to rats (Terada et al., 2017) or images to humans (Reddy et al., 2021). Additionally, the hippocampus is certainly involved in human auditory sequence learning, including when sounds are encountered incidentally. Patients with hippocampal lesions show severe impairment in learning probabilistic relationships between successive pure tones and syllables (Covington et al., 2018, Schapiro et al., 2014), mirroring results in vision (Schapiro et al., 2012). Neuroimaging work in healthy subjects provides further support. Jablonowski et al. (2018). exposed healthy subjects to tone sequence regularities during a learning phase in which they performed an orthogonal sensorimotor task. During a subsequent test phase, they had to decide whether the next tone in a sequence would be higher or lower. Despite subjects having no explicit knowledge of the underlying regularities, accuracy was high and correlated positively with bilateral hippocampal BOLD activity during the learning phase. Relatedly, in a magnetoencephalography (MEG) study, subjects were presented with tones in rapid succession while engaged in an irrelevant visual task. A slow shift in magnetic field strength with a generator in hippocampus (as well as auditory and inferior frontal cortex) occurred from the point at which repetitive structure (recurring frequency patterns) occurred (Barascud et al., 2016).

In addition to these univariate markers of increased activity during sequence learning, representations of specific sound sequences emerge in hippocampus over the course of exposure. In one fMRI experiment, multivariate patterns of activity in left hippocampus came to encode the identity of ordered sequences of spoken letters that repeated over the experiment, even though the individual elements were shared across all sequences (Kalm et al., 2013). Another study exposed subjects to continuous syllable streams, in which particular syllable triplets always occurred in the same order (Henin et al., 2021). While the subjects’ task was to detect the repetition of individual syllables, they implicitly learned the hidden regularities. This was reflected not only in faster reaction times during these structured sequences compared to unstructured ones, but also in patterns of intracranially recorded hippocampal activity that became more similar over time for syllables belonging to the same triplet. In a similar intracranial study, hippocampal activity contained greater power at the (three-syllable) word repetition rate than did auditory cortical activity, with the reverse being true at the syllable repetition rate (Ramos-Escobar et al., 2022). The words that had been implicitly learned subsequently elicited reduced hippocampal evoked responses than did syllable combinations that had not been presented.

Some have argued that sequences of sensory content (or spatial paths) become associated with pre-existing hippocampal cell assemblies that fire in a particular order, while the sensory elements themselves are represented in neocortex (Dragoi and Tonegawa, 2013, Friston and Buzsáki, 2016). Related is the idea of the hippocampus as a predictive (not merely spatial) map (Gershman, 2018, Stachenfeld et al., 2017) that encodes successor representations, namely predictions of future states (discounted future occupancy) given an animal’s current state (Dayan, 1993, Momennejad, 2020). These representations are thought to allow for learning of relational structure – in physical space or otherwise – separate from sensory content or reward contingency, facilitating generalization across environments that share the same relational structure (Geerts et al., 2020, Whittington et al., 2020). A large range of hippocampal findings can be accounted for in this predictive framework, including the modulation of place-cell firing fields by reward locations (Hollup et al., 2001) and barriers (Alvernhe et al., 2011, Muller and Kubie, 1987), and the asymmetric form of place fields during motion along a linear track (Mehta et al., 2000).

Indirect evidence that the hippocampus predicts future auditory content comes from responses to violation of learned rules about sound sequences. Such violations can be considered a form of associative novelty, described in the visual modality by Kumaran and Maguire (2007) and contrasting with the simple stimulus novelty covered at the end of Section 3. Violations of auditory-sequence order elicit scalp components in EEG (Takakura et al., 2003) and MEG (Recasens et al., 2018) that have been localized to hippocampus. In the latter these were accompanied by greater hippocampal theta power and phase locking (Fig. 4D). In probabilistic sound sequences, violation is not all-or-nothing. Instead, time-varying continuous measures of uncertainty (entropy) and surprise can be derived based on learned statistics. Cheung et al. (2019) trained a Markov model on harmonic progressions in pop songs and compared its estimates of uncertainty and surprise during novel progressions to BOLD activity. Anterior hippocampus (along with amygdala and auditory cortex) reflected the interaction between these factors, being elevated when chords deviated substantially from strong expectations, or when they met relatively imprecise ones. Interestingly, these were the same conditions that elicited the greatest pleasure ratings in listeners. Other fMRI work has been more equivocal as to whether hippocampus tracks uncertainty in auditory sequences (Tobia et al., 2012), and in one study the hippocampal BOLD signal was *reduced* in tone sequences in which simple or hierarchical rules concerning pitch and duration were violated compared to when they were met (Martins et al., 2020). Disparate findings may relate to functional heterogeneity of hippocampal fields, position of activity along the long axis, or subtle task differences.

Violation and surprise responses in hippocampus are consistent with it acting as a comparator, with predictions passed from CA3 to CA1 where they are combined with sensory input from entorhinal cortex (Hasselmo and Wyble, 1997, Lisman and Grace, 2005, Vinogradova, 1975b, Vinogradova, 2001). However, there is also evidence for hippocampus sending predictions to sensory cortex. In the study by Recasens et al. (2018), predictable tone sequences elicited elevated effective connectivity (based on alpha-band Granger causality) from right hippocampus to Heschl's gyrus, compared to unpredictable sequences. In other work with perfectly predictable intervals of sound and silence, a spatial independent component of the BOLD signal that included hippocampus led the auditory cortex signal (Langers and Melcher, 2011). In a more specific and naturalistic demonstration of information exchange between hippocampus and sensory cortex, Michelmann et al. (2021) presented a spoken story to intracranially implanted epilepsy patients, repeating the material a second time. Auditory cortical sites were identified where the high-gamma (70–200 Hz) time series showed signs of predictive recall during the second run. At peaks of this predictive measure, mutual information between the auditory cortical high-gamma activity and lower frequency activity in hippocampus was maximal, with hippocampus leading cortical activity by an average of 740 ms (Fig. 4E). In Section 2 (see also Supplementary Table A) we noted pathways from hippocampus via entorhinal, parahippocampal and lateral temporal sites, along which such predictions could be conveyed to auditory cortex. Any error signals resulting from a mismatch between predicted and actually heard sounds could be passed in the reverse direction to update corresponding hippocampal models (Barron et al., 2020).

A missing piece in the puzzle is evidence of specific predicted auditory content being decodable in hippocampus. A number of studies have decoded *visual* content that is predicted on the basis of simple auditory cues from hippocampal BOLD patterns (Aitken and Kok, 2022, Ekman et al., 2022, Kok et al., 2020, Kok and Turk-Browne, 2018). Predicted visual content can also be decoded from hippocampus when that prediction is triggered by auditory cues on the basis of semantic knowledge. In human intracranial recordings, when spoken words primed a particular semantically-related image, high frequency (50–250 Hz) hippocampal activity that was more similar across periods prior to and during the image predicted faster response times (Jafarpour et al., 2017). Furthermore, such activity showed similarity structure across stimuli that reflected the similarity of the predicted objects in semantic space. Another study with a similar task found the most pronounced hippocampal theta activity during sentences and words that set a strong semantic context for the subsequently presented picture (Piai et al., 2016). It remains to be determined whether sounds predicted on the basis of associations learned in the short-term (such as the next note in a melody learned in an experiment) or through semantic context (such as the sound of a bark following a picture of a dog) can be decoded from hippocampal activity before they are heard.

## 10 Navigating frequency space

We have seen that rodent grid cells can provide a basis for spatial navigation (Hafting et al., 2005). In humans, there is evidence that entorhinal grid cells support navigation in virtual (Doeller et al., 2010, Jacobs et al., 2013), imagined (Bellmund et al., 2016) and visual (Julian et al., 2018, Killian et al., 2012, Meister and Buffalo, 2018, Nau et al., 2018) space, as well as time (Ezzyat and Davachi, 2014). Other dimensions of experience can be represented in a similar way; these include social hierarchies spanned by affiliation and power (Tavares et al., 2015), a two dimensional space of body part lengths (Constantinescu et al., 2016), an imagined two dimensional odor space (Bao et al., 2019), and semantic spaces of written words (Solomon et al., 2019). Most of these studies indirectly measure the presence of grid cells by virtue of hexadirectional symmetry of the BOLD response with respect to navigation direction. In some cases, a neural correlate of distance has also been identified in abstract spaces. The evidence is growing that computational circuitry in the hippocampus and entorhinal cortex can facilitate “navigation” through and memory of any arbitrary space.

An important auditory example has been described by Aronov et al. (2017), who trained rats to depress a lever while a tone increased in frequency and to release it in a target frequency range for a reward. They identified CA1 cells that had particular frequency firing fields during this task and others that fired preferentially at the start or end of a trial. The number of tuned cells decreased when the animal was no longer responsible for releasing the lever but was still rewarded when the tone reached the target frequency. When the tone changed in a block without involvement of the animal and in the absence of reward, no such tuning existed. During the active task, cells in the entorhinal cortex could have multiple firing fields. Notably, some of these same hippocampal and entorhinal cells also had place and grid fields when the animal was instead foraging in an open arena. Of all CA1 cells recorded, approximately a quarter had auditory and place fields, a half one or the other, and a quarter neither. This example is different from those described earlier in which discrete individual sounds have acquired behavioral significance (e.g. conditioning studies, Sakurai, 2002) - here the continuous range of presented frequencies is represented. Another important point is that the trial duration and the rate at which the tone frequency changed was varied throughout the experiment. Units retained their frequency tuning across this variability, meaning that they were not simply tuned to the absolute time elapsed in a trial. However subsequent research has established that ensembles of CA1 and entorhinal cells in rats and humans can carry temporal information on a range of scales and individual time cells can stretch their tuning in accordance with the demands of a task (Mau et al., 2018, Reddy et al., 2021, Shimbo et al., 2021, Tsao et al., 2018). It is therefore possible that the units in Aronov et al. (2017) reflected relative time in task (“retime cells” in MacDonald et al., 2011) or relative “distance” to the target sound. The work of Aronov et al. therefore raises intriguing possibilities about the representation of an acoustic dimension in hippocampus during an active task that require critical reappraisal in further experiments.

## 11 Auditory objects and scenes

9, 10 showed that the hippocampus tracks sequences of sound and may map one of its most salient dimensions, frequency. In Section 3 we gave examples of intensity and amplitude modulation rate affecting hippocampal responses and also described how *changes* in sound features can drive hippocampal responses through release from habituation. In cluttered acoustic scenes, the rate of change of sound features is among the key determinants of which sequential elements should be grouped into auditory streams or objects (Bregman, 1990, van Noorden, 1975). An auditory perceptual object, like its visual counterparts, is defined by the binding of multiple sensory features, represents a source distinct from others in the scene, and is invariant over different sensory instances (Bizley and Cohen, 2013, Griffiths and Warren, 2004, Kubovy and Van Valkenburg, 2001). The medial temporal lobe is important in visual object processing and although the hippocampus itself is not critical here, it does support the construction of scenes – configurations of objects in space (Barense et al., 2009, Bussey and Saksida, 2005, Chadwick et al., 2013, Hassabis et al., 2007, Lee et al., 2012, McCormick et al., 2021, Mullally et al., 2012, Smith et al., 2014, Zeidman et al., 2015).

Does the medial temporal lobe contribute to the formation of auditory objects or their collection into scenes? Much of the abstraction of different auditory features and their combination into object representations is accomplished in the ascending subcortical auditory pathway and auditory cortex. For example, adaptation occurs in the auditory nerve for grouping of harmonics by common onset (Holmes and Roberts, 2011) and tuning for combinations of auditory features is present in primary auditory cortex (Bizley et al., 2009). Representations in auditory association cortex demonstrate object-level intensity gain control (Simon, 2015) and correlate with object-level perception (Billig et al., 2018) and attention (O’Sullivan et al., 2019). The extraction of timbre, defined by sound features other than intensity and pitch, involves higher auditory-associated cortex including planum temporale and anterior superior temporal sulcus (Kumar et al., 2007, Warren et al., 2005). Selectivity for phonemes defined by particular combinations of spectrotemporal features is found on the superior temporal gyrus (Mesgarani et al., 2014) and illusory percepts of vowels based on apparent object continuity have correlates in superior temporal sulcus and middle temporal gyrus (Heinrich et al., 2008). Although the medial temporal lobe could provide top-down object information to these earlier sites, unlike in vision (Devlin and Price, 2007) evidence for its involvement in auditory object formation, perception, and scene analysis is scarce (Bizley and Cohen, 2013, Christison-Lagay et al., 2015, Griffiths and Warren, 2004, Snyder and Elhilali, 2017). Patients with Alzheimer’s disease show impairment in auditory segregation and scene analysis tasks, but structural and functional correlates of these deficits are reported in lateral temporal and parietal cortices, rather than in the medial temporal lobe (Golden et al., 2015, Goll et al., 2012). However there is some support for the number of perceptual objects in a simple acoustic scene being tracked there. A human intracranial study found that hippocampal activity distinguished between perceptual interpretations of bistable tone triplets that could be heard as one or two streams (Curtu et al., 2019). Another human study found a medial temporal source for a late P400-like scalp potential associated with successful detection of a mistuned harmonic in a tone complex, which gives rise to the percept of two concurrent auditory objects (Alain et al., 2001; Fig. 4F). In rodents, damage to perirhinal cortex (hippocampus was not tested) impaired rats in binding discontinuous temporal vocalization elements into an object to act as a conditioned stimulus in fear conditioning (Bang and Brown, 2009) and we have already described involvement of hippocampus in bridging temporal gaps, both during auditory working memory and trace conditioning, and in representing auditory sequences in memory. Less direct evidence for hippocampal involvement in auditory scene segregation is its elevated activity in subjects performing a verbal working memory task in noise compared to in quiet (Manan et al., 2012).

Two important ingredients to successful parsing of an auditory scene are the ability to distinguish an object of interest from the background, and to restore a partially masked sound on the basis of prior knowledge. These requirements to "separate" and "complete" auditory representations under different circumstances bring to mind two terms describing particular computations in support of memory, thought to involve hippocampus (Marr, 1971, McNaughton and Morris, 1987, Rolls, 2013). Pattern separation refers to the storing of distinct activity patterns for memories that share similar features. Modeling suggests that the large number of granule cells in dentate gyrus can support the sparse coding necessary to transform the overlapping representations from entorhinal cortex and project the result to CA3 and beyond for storage and consolidation. In the case of an unfolding and spectrotemporally overlapping acoustic scene, might this separation act rapidly enough for the results to be read out during online perception? The hippocampus can certainly guide perceptual sampling of a cluttered visual scene (Kragel et al., 2021) and the same may be true of audition. Impaired perceptual discrimination of complex visual objects in a patient with relatively selective dentate gyrus lesions also supports the idea that pattern separation in this hippocampal subfield is relevant not only for memory, but also online perception (Mitchnick et al., 2022).

The other concept, pattern completion, refers to the retrieval of a memory on the basis of a partial cue, thought to be supported by auto-associative or attractor networks in CA3 (Rolls, 2013). Such a completion process could potentially retrieve features of known sources (e.g. the vocal characteristics of a known conversation partner) or anticipate likely continuations of interrupted sentences based on semantic knowledge or prior exposure. In this vein, one study found theta synchrony between medial temporal lobe (only parahippocampal gyrus was available for analysis) and auditory cortex during the illusory continuation of familiar music when interrupted by noise (Müller et al., 2013). The passing of predictions from hippocampus to auditory cortex based on learned sequences and discussed in Section 9 would also constitute a form of pattern completion. These ideas remain mostly speculative, and a critical role for hippocampus in auditory object formation has not been demonstrated. Alternatively, it is possible that hippocampus is only required when incorporating auditory elements into scenes primarily determined by visual objects defined in a spatial framework. In the next section we consider auditory-visual and other crossmodal interactions in hippocampus.

## 12 Multi- and supra-modal representations and associations

The intrinsic circuitry and external connectivity of the hippocampus allow it to bind sensory experience across modalities. Patients with hippocampal lesions have impaired memory for associations between simultaneously presented faces and voices (Mayes et al., 2004, Vargha-Khadem et al., 1997), and between other sounds and scenes (Mayes et al., 2004) or abstract images (Borders et al., 2017). At the same time, transcranial magnetic stimulation of parietal sites identified in individual subjects to be functionally connected to hippocampus boosts memory for word-face pairs (Wang et al., 2014). Hippocampal BOLD activity during encoding of an object in memory scales with the number of features to be integrated, with location making a greater contribution than color or sound (Cooper and Ritchey, 2020). Activity is also elevated for successful encoding or retrieval of cross-modal associations compared to within-modality pairs (Butler and James, 2011, Gottlieb et al., 2010, Joassin et al., 2011, Persson et al., 2011) and demonstrates functional connectivity with cortical sites including superior temporal gyrus during such multimodal associations (Cooper and Ritchey, 2019, Griffiths and Fuentemilla, 2019, Joassin et al., 2011, Love et al., 2011). In an experiment involving memory for text-sound associations, pairings with the greatest hippocampal activity at encoding were recalled most accurately and showed the most similar neocortical patterns across encoding and retrieval (Danker et al., 2016). In these experiments, learning the association was explicitly required as part of the task. However, elevated hippocampal BOLD has also been demonstrated when images are combined with emotionally congruent music compared to when they are presented alone during an emotion-rating task without an explicit memory component (Baumgartner et al., 2006).

Tone frequency can combine with a non-auditory dimension to define a semantic space that is represented in the medial temporal lobe. Viganò and Piazza (2020) trained participants to associate particular quadrants in a two-dimensional space of visual shape and tone frequency with four different non-word labels. After training, right entorhinal cortex showed tuning to direction of navigation through this space during a one-back task that used both the audiovisual objects and the written semantic labels. Auditory stimuli can also trigger or be subsumed into super-modal conceptual representations in the hippocampus. For example, Quiroga and colleagues have identified human hippocampal units that respond selectively to famous people, whether in photograph form, or as a written or spoken name (Quiroga, 2020, Quiroga et al., 2009). Twice as many neurons respond to the image than to the sound in these studies, and while there are neurons that respond to the image but not the sound, the reverse is not true (however this bias may be a result of the distribution of stimuli used). To establish the extent to which auditory representations are subordinate in the hippocampus it would be valuable to establish whether such concept tuning can be identified based on multiple auditory instances only, such as the spoken word "dog" and the sound of a dog barking, or a person's spoken name and their voice.

## 13 Auditory elements of episodic memories and their consolidation

The hippocampus not only binds across sensory modalities, but situates these multimodal objects in a spatiotemporal context to form memories of particular episodes (Gelbard-Sagiv et al., 2008, Paz et al., 2010). We note the apparent contradiction that under its proposed role in statistical learning the hippocampus generalizes to learn the probabilities of transitions between sounds over multiple presentations, but it is also able to form discrete memories for individual episodes. Modeling work suggests that statistical learning could rely more on the monosynaptic connection between entorhinal cortex and CA1, where inhibition and sparsity are less pronounced, than along the trisynaptic pathway from entorhinal cortex through dentate gyrus and CA3, likely important for memory of individual episodes (Schapiro et al., 2017).

The extent and nature of the hippocampus' ongoing involvement in maintaining and retrieving episodic memories has been controversial. The standard consolidation model (Squire and Zola-Morgan, 1991, Squire and Alvarez, 1995) holds that episodic memories that initially depend on hippocampus to index distributed content in neocortex (Teyler and DiScenna, 1986) become consolidated over time, with direct links between those neocortical sites strengthening, and hippocampal dependence declining. Such a model accounts for the graded retrograde memory deficit observed in amnesic patients such as H.M., whereby older memories are relatively preserved - however, it has been argued that these are of more of a semantic nature than vividly episodic. Alternative accounts – such as multiple trace, trace transformation, and contextual binding theories - propose that hippocampus continues to be involved, either in indexing or reconstructing distributed cortical content, or maintaining such content itself (Nadel et al., 2000, Winocur and Moscovitch, 2011, Yonelinas et al., 2019).

Sound-related evidence for ongoing hippocampal involvement in supporting rich memories (rather than memories that are given a semantic label), comes from findings that BOLD activity there scales with the vividness of a retrieved episodic memory, including its auditory content (Sekeres et al., 2018). Furthermore, patients with medial temporal lobe damage report fewer perceptual (including auditory) details when retrieving episodic memories than controls (St-Laurent et al., 2014). Hippocampal BOLD activity is elevated when hearing a recording of one's own autobiographical memories (Svoboda and Levine, 2009) or a melody previously associated with a particular object and location (Prabhakar et al., 2018), in both cases after several days. When listening to familiar music, subjects show greater hippocampal BOLD activity as they retrieve specific autobiographical episodes associated with the music than more general ongoing events or personal knowledge from the relevant period in their lives (Ford et al., 2011). Hippocampus is also more active during such retrieval than when attending to structural features of the music (Kubit and Janata, 2018).

Which memories get consolidated in humans during sleep can be biased by presentation of relevant sounds. In one study, subjects learned object-location pairs while presented with an object-specific sound, then slept during a scanning session (van Dongen et al., 2012). Greater hippocampal activity when previously heard sounds were repeated during sleep was associated with better retention of object-location pairs as tested the following day. In another study, participants learned motor patterns that were associated with different tones. Presentation of one of those tones during sleep led to faster execution of the cued compared to the uncued pattern the following day, with a corresponding difference in bilateral hippocampal activity and hippocampal connectivity to motor areas (Cousins et al., 2016). In rodents, hippocampal involvement in consolidation of auditory memories during sleep was demonstrated by Bendor and Wilson (2012), who first paired spatial trajectories with auditory cues in behaving rats. Presenting one of these sounds during subsequent sleep increased the probability that the place cell sequence encoding the related trajectory would be reactivated. In another study, auditory cortical patterns that occurred when rats approached a location associated with a sound were recapitulated during sleep, both spontaneously and when cued by the auditory stimulus (Rothschild et al., 2017). This activity predicted subsequent hippocampal sharp-wave ripples, which in turn predicted subsequent auditory cortical activity, suggesting a bidirectional exchange of information. Content-specific sharp-wave ripple playback from hippocampus to sensory cortex has also been detected in humans, but so far only during awake visual recall (Norman et al., 2019). The fact that auditory signals, unlike visual information, can trigger hippocampal activity during sleep points to possible overnight learning applications (Harrington and Cairney, 2021), discussed further in Section 18.

## 14 Perception and memory of speech and music

Speech (Supplementary Tables F, J) and music (Supplementary Tables G, K) are two classes of sound particularly important in human communication. A small proportion of hippocampal neurons respond selectively to specific spoken words (Urgolites et al., 2022), and in challenging listening conditions the distinctness of candidate word representations in left hippocampus is positively correlated with speech understanding (Blank et al., 2018; Fig. 4G). Intelligible speech elicits greater hippocampal activity than unintelligible speech, regardless of whether that greater intelligibility is due to acoustic clarity or provision of prior information (Clos et al., 2014, Davis et al., 2011, Davis and Johnsrude, 2003). The hippocampus is also important in resolving syntactic or semantic ambiguity based on information from earlier in a sentence or exchange (Kurczek et al., 2013, Rubin et al., 2011). Another potential role of hippocampus during conversation is in monitoring one's own speech, perhaps through comparing predictions of motor commands with resulting auditory feedback (van de Ven et al., 2020; see also Rummell et al., 2016 for self-generated sounds in mice).

Beyond online speech perception there is substantial neuropsychological evidence for left hippocampus in particular playing a key role in the learning and recall of verbal material (Barbeau et al., 2005, Boon et al., 2011, Cavazzuti et al., 1980, Coras et al., 2014, Dulay et al., 2004, Frisk and Milner, 1990, Gadian et al., 2000, Goldstein et al., 1988, Helmstaedter et al., 1997, Helmstaedter and Elger, 1996, Huijgen et al., 2015, Jayakar et al., 2015, McMillan et al., 1987, Meyer and Yates, 1955, Mueller et al., 2012, O’Brien et al., 2003, Rausch and Crandall, 1982, Squire et al., 2001, Tachibana et al., 1999, Vargha-Khadem et al., 1997, Witt et al., 2014). Neuroimaging data support this lateralization, with left hippocampal BOLD signal during exposure to novel pseudo-words correlating across subjects with subsequent recall (Davis et al., 2009; Fig. 4H). Not only the level of activity in the hippocampus (Kato et al., 1998, Park and Rugg, 2009, Petersson et al., 1999, Schmithorst et al., 2006, Urgolites et al., 2020) but also its degree of functional connectivity with lateral temporal cortex is associated with successful encoding of speech in memory (Babiloni et al., 2009, Gagnepain et al., 2011). During continuous speech, such encoding may occur during moments of increased connectivity at perceived event boundaries (Michelmann et al., 2021). This is consistent with hippocampus segmenting ongoing experience, such as when it marks moments of narrative shift in movies (Baldassano et al., 2017, Ben-Yakov and Henson, 2018) or identifies recurring words in a continuous stream of syllables during statistical learning (Henin et al., 2021, Ramos-Escobar et al., 2022). The novelty of both a word (Davis et al., 2009) and its category (Dolan and Fletcher, 1997) can drive hippocampal activity during memory encoding. However single unit recordings from human hippocampus reveal neurons that respond preferentially to repeated words alongside those driven by word novelty (Urgolites et al., 2022).

While the above studies point to a role for hippocampus in the *encoding* of memory for words, deficits in *consolidation* have been identified in groups with presumed hippocampal damage. Patients with transient epileptic amnesia (Hoefeijzers et al., 2013) and with presymptomatic autosomal dominant Alzheimer's disease (Weston et al., 2018) show accelerated long-term forgetting, such that word memory is impaired after a week, but not after 30 min. This suggests that verbal acquisition itself may not be impaired when the hippocampus is compromised, but rather the durability of the memories that form. A role of hippocampus in speech memory consolidation is also supported by a study of healthy subjects, in whom hippocampal volume correlated positively with post-training overnight change in non-native speech sound discrimination (Fuhrmeister and Myers, 2022).

Whereas verbal memory deficits are associated particularly with left hippocampal damage, memory for melody may depend to a greater extent on right-hemisphere structures. However, although right hemisphere damage has been linked to selective impaired memory for melody versus lyrics (Samson and Zatorre, 1992, Samson and Zatorre, 1991), and to a reduced “mere exposure” effect where previously heard melodies are usually judged as more likeable than novel ones (Samson and Peretz, 2005), lesions in these studies extended to extra-hippocampal temporal regions important in music perception and discrimination (Milner, 1965, Samson and Zatorre, 1994, Zatorre, 1984 but see Koike and Ishijima, 1996). Indeed, a number of neuropsychological cases indicate that intact hippocampi are not necessary for a range of musical perceptual abilities relating to timing and pitch (Esfahani-Bayerl et al., 2019), nor for learning to play new music (Cavaco et al., 2012, Valtonen et al., 2014) or discriminating pieces from closely matched ones heard a short time earlier (Finke et al., 2012). These findings are consistent with intact musical memory in patients with Alzheimer’s disease, in whom other hippocampal-dependent memories are impaired (Baird and Samson, 2009, Cuddy et al., 2015).

However, as we have seen in earlier sections, the hippocampus not being critical for a task does not prevent it from tracking relevant stimulus or behavioral variables. For example, during listening to a rich naturalistic stimulus in the absence of a task, hippocampal BOLD activity was associated with repeated occurrences of musical motifs after regressing out acoustic predictors (Burunat et al., 2014; Fig. 4I), and showed functional connectivity with regions involved in holding melodies in mind, including dorsolateral prefrontal cortex, cerebellum, and the supplementary motor area. Schmithorst (2005) also identified melody-specific activity in hippocampus using an independent components analysis of fMRI data during passive listening, and in a study of memory for newly-learned melodies retrieval success was associated with greater right hippocampal BOLD signal (Watanabe et al., 2008). As with speech there is electrophysiological evidence for greater hippocampal processing at musical phrase boundaries (Knösche et al., 2005).

Hippocampal activity and connectivity has been detected during a range of tasks during music listening, including tone detection (Lehne et al., 2014), timbre and tonality deviant detection (Janata, 2002), temporal order judgments (Mueller et al., 2015), spontaneity judgments (Engel and Keller, 2011), and memory encoding (Bonetti et al., 2021). Hippocampus is also activated during passive music listening, compared to a silent or scrambled baseline (Brown et al., 2004, Mueller et al., 2015, Mueller et al., 2011, Mutschler et al., 2010). We will see in 15, 16 that familiarity and emotional aspects of music may partly drive these responses.

## 15 Long-term familiarity

4, 5 covered responses to sounds that had recently acquired behavioral relevance, for example through aversive conditioning or prior exposure in a target detection task. Longer-term familiarity with an auditory stimulus also affects the magnitude of the hippocampal activity it drives. The hippocampus of rabbits, cats, and monkeys can show greater responses to familiar sounds, such as hisses, than to louder but unfamiliar synthetic sounds, such as tones and clicks (Grastyán et al., 1959, Green and Arduini, 1954, Tamura et al., 1990). Note that greater responses to familiar than unfamiliar sounds contrast with the novelty responses described in 3, 14, which presumably arise through different mechanisms operating over a shorter timescale.

While in the above studies not only the familiarity but also the gross acoustical features of the contrasted sounds differed, other work has attempted to control the latter. For example, when children listened to their mother’s voice, fMRI connectivity across hippocampus, reward- and voice-sensitive regions was greater than when listening to other female voices (Abrams et al., 2016). Long-term familiarity with verbal expressions correlated with posterior hippocampal BOLD activity; this was not the case for musical melodies presented to the same subjects (Gagnepain et al., 2017, Groussard et al., 2010b). There are other indications that the hippocampus is relatively unimportant in familiarity processing of music. Using multivoxel pattern analysis, long-term familiar songs could be distinguished from songs heard on the same day or novel songs in anterior cingulate and pre-supplementary motor area, but not in the medial temporal lobe (Jacobsen et al., 2015). Subjects listening to their favorite song showed less auditory-hippocampus connectivity than when listening to other songs in the same genre (Wilkins et al., 2014). Additionally, a study of patients with medial temporal lobe resections were impaired in verbal learning and recall but had a spared feeling of familiarity when hearing short excerpts of well-known music (Huijgen et al., 2015).

These negative findings are consistent with visual work identifying a greater dependence on hippocampus (and connected diencephalic structures) for explicit recollection of an episode, compared to general familiarity likely to be supported more by parahippocampal/perirhinal circuits (Aggleton and Brown, 2006, Brown and Aggleton, 2001, Tsivilis et al., 2008). However, for audition the story is mixed, with some studies finding greater responses in hippocampus for familiar compared to unfamiliar music (Pereira et al., 2011, Plailly et al., 2007) and deficits in recognizing well-known songs in patients with hippocampal damage (Papp et al., 2014). In addition, it is not always straightforward to isolate processes relating to familiarity from those involved in explicit recollection, as familiar sounds can elicit the recollection of particular events.

## 16 Emotion and sound

Also hard to dissociate from sound familiarity are sound preference and other emotional drivers of hippocampal activity, both due to “mere exposure” effects of repetition (Hunter et al., 2010, Samson and Peretz, 2005) and the fact that people choose to listen to music they enjoy. In some experiments that show greater hippocampal BOLD activity for familiar than unfamiliar music, stimulus manipulations affect both long-term familiarity and pleasantness ratings (Koelsch et al., 2006, Mueller et al., 2015, Mueller et al., 2011, Pereira et al., 2011). Particularly intense emotional responses to music can be evoked by familiar music that listeners select for themselves. Blood and Zatorre (2001) reported reduced hippocampal blood flow when listening to self-selected music experienced as intensely pleasurable, eliciting “chills” and other physiological responses, in comparison to control music selected by other subjects. This reduced response is thought to arise through inhibitory projections from the nucleus accumbens, part of the dopaminergic reward system in which activity increases during chills. These two regions demonstrate increased BOLD functional connectivity during music to which listeners assign increasing value - even new music with which they are not familiar (Salimpoor et al., 2013).

Medial temporal sites - and the focus is more parahippocampal gyrus than hippocampus proper - are involved in processing emotion in music, beyond the familiarity effects described above. Lesion (Gosselin, 2006) and neuroimaging (Blood et al., 1999, Ferri et al., 2014) data link the rating of dissonant harmonies as unpleasant to parahippocampal gyrus, paralleling work with unpleasant images (Lane et al., 1997). Diffuse medial temporal lesions affect more general estimates of pleasantness and arousal in music (Gosselin et al., 2005, Khalfa et al., 2008) and impair recognition of musical emotion (Dellacherie et al., 2008, Gosselin et al., 2011, Gosselin et al., 2005, Omar et al., 2011, Papp et al., 2014) (but see Dellacherie et al., 2011), and posterior hippocampal gray matter volume is greater in frontotemporal dementia patients with musicophilia, (an abnormal craving for and delight in music) than in those without (Fletcher et al., 2013).

Not only simple musical emotional contrasts (such as happy versus sad, joyful versus fearful) but also subtler differences involving arousal or valence alone modulate (para)hippocampal BOLD activity and its relationship with that in auditory and reward networks (Flores-Gutiérrez et al., 2007, Koelsch et al., 2018, Koelsch et al., 2013, Koelsch and Skouras, 2014, Trost et al., 2012), and individuals with high alexithymia scores show less such modulation (Koelsch et al., 2007). Some of these effects may arise through the uncertainty and surprise associated with harmonic progressions, which jointly predict pleasantness ratings and the BOLD response in regions including hippocampus as described previously (Cheung et al., 2019).

Section 12 considered hippocampal correlates of multimodal stimulus processing; these are also apparent with respect to emotionally-charged material. For example, combining emotional music and pictures elicits greater (para)hippocampal BOLD than the pictures alone (Baumgartner et al., 2006) and the presence of narrative action in a neutral film increases modulation of hippocampal activity by emotional music (Eldar et al., 2007). However, shutting off uninformative input by closing the eyes leads to more extreme valence ratings and elevated hippocampal BOLD in response to emotional music (Lerner et al., 2009). Not only the structural elements of a musical piece but also expressive performance determine its emotional profile; this too affects hippocampal activity (Chapin et al., 2010a, Engel and Keller, 2011). The involvement of hippocampus in processing emotional music occurs regardless of expertise and although it can habituate over the course of listening it does not generally require active engagement (Brown et al., 2004, Chapin et al., 2010b, Mutschler et al., 2010, Pallesen et al., 2009).

Music is not the only means of expressing emotion through sound. (Para)hippocampal areas are also sensitive to different types of laughter (Szameitat et al., 2010), as well as to prosodic (Wiethoff et al., 2008) and semantic (Bellace et al., 2012, Mitchell et al., 2003) emotion cues in speech. These sites are more sensitive to natural than to computer-generated speech (Beaucousin et al., 2006), and respond to or support processing of vocal fear, anger, happiness, surprise, disgust (Bonora et al., 2011, Fowler et al., 2006, Kotz et al., 2013, Leitman, 2010, Phillips et al., 1998, Sander et al., 2005), as well as more complex auditory emotional expression such as pride, guilt, and boredom (Alba-Ferrara et al., 2011). Several studies describe functional connectivity signatures of auditory emotion processing. For example, alarm sounds rated as highly unpleasant are associated with reduced hippocampal BOLD activity and functional connectivity consistent with suppression by the amygdala (Hirano et al., 2006). This coincides with worse encoding of a concurrent visual stimulus, in line with findings in animal models showing that stress and fear impair learning. Subjects with misophonia, for whom everyday sounds evoke strong negative emotional responses, show elevated functional connectivity between anterior insula and a salience network that includes hippocampus (Kumar et al., 2017). Individual differences in responses to aversive sounds may also have structural correlates – in one large study, subjects’ sensitivity to a noise stimulus correlated with hippocampal gray matter volume (Kliuchko et al., 2018). In another study, synthetic sounds rated as more aversive generated elevated cerebral blood flow in (para)hippocampus and amygdala (Mirz et al., 2000). Negative reactions to certain sounds may relate to the energy in the “roughness” range of 30–150 Hz; click trains presented at this rate are rated as highly salient and aversive and bring about neuronal synchronization at the presentation rate, particularly in the hippocampus and insula (Arnal et al., 2019).

## 17 Phantom percepts

Aversive auditory experiences do not require an external stimulus. Tinnitus (see Supplementary Table L) usually takes the form of a low-intensity, high-frequency ringing or white noise that is typically readily masked by environmental sounds but can be chronic and emotionally distressing (Jastreboff, 1990). It tends to co-occur with hearing loss, and many theories of its generation are based on maladaptive change to deafferentation, or deficient noise-canceling (Jastreboff, 1990, Noreña, 2011, Rauschecker et al., 2010, Schaette and Kempter, 2006). In-depth reviews and integrative accounts are available elsewhere (De Ridder et al., 2014, Sedley et al., 2016); here we focus on correlates in (para)hippocampus but note that comorbidity of tinnitus with hearing loss, distress, depression, cognitive dysfunction and insomnia presents a challenge in establishing specific neural signatures (Adjamian et al., 2014, Bhatt et al., 2017, Crönlein et al., 2007, Hallam et al., 2004, Hwang et al., 2009, Melcher et al., 2013). At the molecular level, damage to rat inner hair cells leads to tinnitus-like behavior only for those animals whose hippocampus and auditory cortex fail to mobilize Arc (Singer et al., 2013), a protein involved in long-term potentiation and adjusting synaptic transmission following sensory deprivation (Korb and Finkbeiner, 2011). Tinnitus-like behavior is also present in rats with noise-induced disruption to neurogenesis and cholinergic and GABAergic pathways in hippocampus (Kraus et al., 2010; L. Zhang et al., 2019; Zhang et al., 2021). In terms of gross structure, the volume of left hippocampus is smaller in patients with tinnitus than in controls matched for hearing loss (Boyen et al., 2013, Landgrebe et al., 2009) and its surface area correlates negatively with tinnitus handicap inventory scores (Tae et al., 2018). Hippocampal gray matter abnormalities have been identified in tinnitus sufferers using diffusion tensor imaging (Gunbey et al., 2015), and tinnitus symptoms have been reported after hippocampus damage or resection (Corkin et al., 1997, Kreyberg et al., 1992, Paquette et al., 2017, Rey et al., 1984).

These negative links between hippocampal structure and tinnitus stand in distinction to functional studies that often (but not always, Shulman, 1995; Shulman et al., 1995; Simonetti et al., 2022) report a positive association between hippocampal activity and tinnitus loudness or incidence (De Ridder et al., 2006, Lockwood et al., 1998). Resting state fMRI indicates greater connectivity of the hippocampus with auditory cortex and beyond for louder tinnitus percepts and longer tinnitus durations (Chen et al., 2017b, Ueyama et al., 2013), and highlights left hippocampus as a key node in functional networks of chronic tinnitus patients compared to controls (Lan et al., 2022). In contrast, functional connectivity between hippocampus and subcortical auditory nuclei is reduced in rats with salicylate-induced tinnitus and hyperacusis symptoms (Chen et al., 2015); these animals also show elevated hippocampal local field potential responses to noise-bursts compared to controls (Chen et al., 2014). Distinct neural correlates of salicylate- and noise exposure have been described and these complicate the search for a unified signature of tinnitus (Eggermont, 2013). In humans, regional blood flow in other medial temporal structures, including amygdala and parahippocampal gyrus, also differs across suppressed versus active tinnitus states (Mirz, 2000) and levels of distress (Schecklmann et al., 2013), as well as between participants with tinnitus compared to hearing-impaired and normal-hearing controls (Carpenter-Thompson et al., 2014, Laureano et al., 2014). Indeed, resting state activity in parahippocampal gyrus more consistently differentiates these groups than does activity in hippocampus proper (Chen et al., 2017a, Song et al., 2012) and may form an important tinnitus hub (De Ridder et al., 2014, Sedley et al., 2015).

Auditory hallucinations (see Supplementary Table M) are another example of sound perception in the absence of a stimulus. These have been associated with hippocampal lesions in a number of reports, but rarely isolating the hallucinations from comorbid symptoms, for example in patients with schizophrenia (Maller et al., 2012, Suzuki et al., 2003, Takebayashi et al., 2002). A similar caveat holds for early imaging studies that reported increased medial temporal lobe activity in patients with schizophrenia featuring auditory hallucinations (DeLisi et al., 1989, Friston et al., 1992, Liddle et al., 1992, Medoff et al., 2001, Volkow et al., 1987) although some studies are more specific, with greater medial temporal cerebral blood flow for auditory compared to tactile hallucinations (Musalek et al., 1989) or with longer durations of auditory hallucination (Copolov et al., 2003). Within-subjects comparisons have revealed greater (para)hippocampal activity for periods with auditory hallucinations compared to those without (Dierks et al., 1999, Raij et al., 2009, Shergill, 2001, Shergill et al., 2000, Silbersweig et al., 1995). As with tinnitus, compared to hippocampus proper (Bentaleb et al., 2002, Jardri et al., 2009, Jardri et al., 2007, Lennox et al., 2000, McGuire et al., 1993, Sommer et al., 2008), the involvement of parahippocampal gyrus is most reliable across studies (Diederen et al., 2010, Hoffman et al., 2008, Jardri et al., 2011). In both cases parahippocampal gyrus may convey aberrant predictions originating in hippocampus to auditory cortex, but the relative lack of corresponding hippocampus activity remains to be explained. The presence of auditory auras in a subset of patients with mesial temporal lobe epilepsy is also relevant (Asadi-Pooya et al., 2016, Ferrari-Marinho et al., 2012). One striking case is that of a patient with a hippocampal seizure focus, who experienced ringing in both ears prior to seizures. Electrical stimulation of the hippocampus generated the same percept, which did not recur once the tissue was resected (Kumar et al., 2022).

## 18 Short-term effects of sound on non-auditory tasks and hippocampal activity

**Fig. 5:**
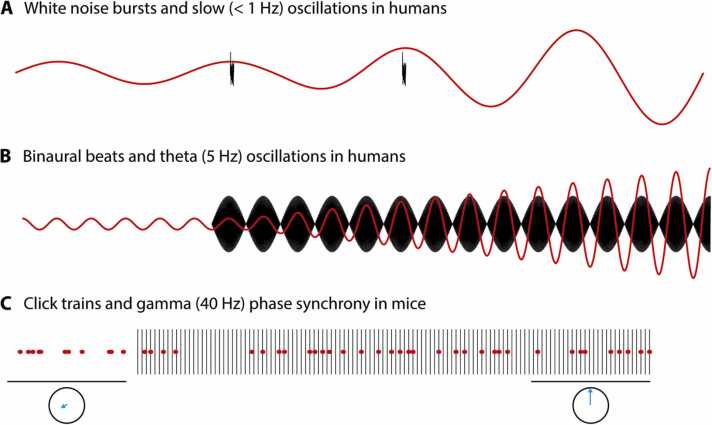
Auditory entrainment of hippocampal rhythms (see Section 18 of main text for related behavioral outcomes). (A) Suitably timed white noise bursts (black) boost widespread cortical slow (< 1 Hz) oscillations (red), which propagate to hippocampus and synchronize sharp-wave ripples (not shown) in humans (Ngo et al., 2013). (B) Dichotically presented pure tones separated in frequency by 5 Hz generate binaural beats (black) and boost hippocampal theta oscillations (red) in humans (Derner et al., 2018). (C) 40 Hz click trains (black) affect firing of hippocampal CA1 units (red dots), increasing phase synchrony (blue arrows) at the same (gamma) frequency in mice (Martorell et al., 2019).

We have described immediate responses to sound in the hippocampus and its possible involvement in a range of auditory tasks, functions and percepts. In Section 2 we also outlined effects of electrically or optogenetically stimulating hippocampus on auditory brain regions. Here we cover cases in which sound stimulation instigates, boosts or entrains hippocampal oscillations to affect non-auditory cognitive function. We consider oscillations over three timescales, as shown in Fig. 5. First, slow (< 1 Hz) oscillations reflect global fluctuations in cellular excitability - alternating phases of hyperpolarization and depolarization across large populations of neurons. In humans these oscillations are likely driven by prefrontal cortex but propagate via parahippocampal and entorhinal cortex to hippocampus (Nir et al., 2011) where they can synchronize ripple events (Born and Wilhelm, 2012). Introducing short bursts of pink noise at the peak of the up state of these oscillations during sleep increases their amplitude and leads to improved memory encoding the following day (Ngo et al., 2013; Fig. 5A). It has not been possible to directly record from hippocampus during overnight auditory stimulation, however in one study the pink noise protocol was applied during a nap, and a picture encoding task was performed subsequently in a scanner (Ong et al., 2018). The magnitude of slow oscillation enhancement during the nap correlated with picture encoding success and with hippocampal BOLD during encoding. This may indicate that boosting slow oscillations allowed previous memories to be consolidated to cortex, freeing up hippocampal resources for subsequent encoding.

The second timescale of oscillations at which auditory entrainment may boost hippocampal function is the theta range. Roberts et al. (2018) presented audiovisual stimuli at either 5.5 Hz or 14 Hz, or white noise, between training and testing of verbal memory. In the 5.5-Hz condition only, theta power recorded at the scalp was enhanced both during entrainment and subsequent retrieval, and hippocampus-dependent source memory was selectively boosted. Other studies have generated binaural beats in the theta range by presenting pure tones of frequencies differing by 5 Hz to separate ears, then directly measuring hippocampal activity in neurosurgical patients (Derner et al., 2021, Derner et al., 2020, Derner et al., 2018; Fig. 5B). Binaural beats were associated with improved source and item memory along with increased phase synchrony and unit firing in human (para)hippocampus, with firing rate differences between monaural and binaural beat conditions correlating across subjects with differences in memory performance.

Third, 40-Hz click trains or stimuli amplitude-modulated at this rate have long been known to elicit a strong steady state response in human auditory cortex (ASSR, Galambos et al., 1981). However they also modulate unit activity in mouse hippocampus (as well as auditory cortex and medial prefrontal cortex) such that firing tends to cluster at fixed phases of the 40-Hz cycle (Martorell et al., 2019; Fig. 5C). Remarkably, click trains presented at this rate (but not with random timing) reduced signs of Alzheimer's pathology (amyloid load and tau phosphorylation) in the hippocampus of this mouse model and boosted memory for the identity and location of objects. Effects were larger when the auditory stimulus was paired with visual flicker at the same rate - this combined stimulation also induced an increase in 40 Hz power and a clustering effect of microglia around amyloid deposits. The implications for dementia treatment are considerable, and work is ongoing to test the same approach in human trials (Chan et al., 2021). The mechanism is not yet understood but networks of inhibitory interneurons generate peaks at this low-gamma frequency, which may also be involved in coupling CA3 and CA1 during memory retrieval (Colgin et al., 2009, Mably and Colgin, 2018).

## 19 Long-term experience with sound and its absence

**Fig. 6:**
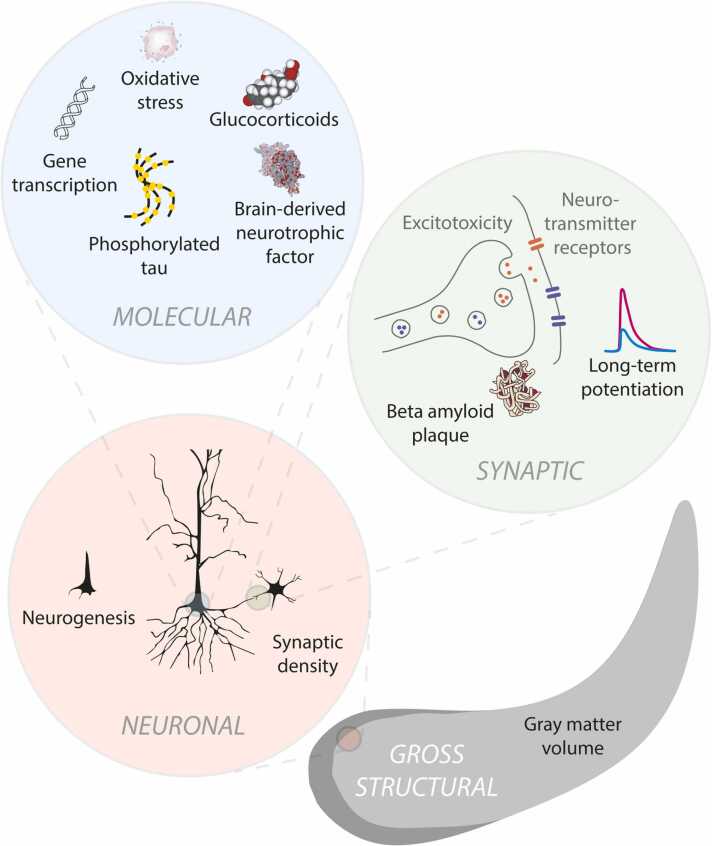
Examples of processes at molecular, synaptic, neuronal and gross structural hippocampal levels on which auditory experience (such as music listening or training, noise exposure, and auditory deprivation) can act. See Section 19 of main text and Supplementary Table N for details and references. The causal pathways underlying such effects are largely yet to be established.

Having considered effects of short-term auditory exposure we turn now to longer-term experience with sound and its positive and negative effects on hippocampal structure and function (see Supplementary Table N and Fig. 6). If the hippocampus maps out non-spatiotemporal dimensions as suggested in Section 10 then experts at navigating such dimensions might be expected to demonstrate gross anatomical differences, given the finding of Maguire et al. (2000) that London taxi drivers have enlarged posterior hippocampi. Such associations have been found, with positive relationships between years of training and anterior hippocampal gray matter volume in musicians (Groussard et al., 2014, Groussard et al., 2010a) and piano-tuners (Teki et al., 2012). For spatial navigation and other hippocampus-dependent tasks, the generalizability of a volume-function relationship to a non-expert population has not been demonstrated (Clark et al., 2020, Weisberg and Ekstrom, 2021). However with respect to music, the volume of parahippocampal gyrus (hippocampus was not tested) was found to correlate positively with an index of musical sophistication in a group of 73 older adults after controlling for intracranial volume (Chaddock-Heyman et al., 2021). Longitudinal studies, similar to those in taxi drivers (Woollett and Maguire, 2011), will be important to establish causality in any of the above relationships.

Functional differences in hippocampus of musicians and non-musicians have been identified through task-related BOLD activity and connectivity (Alluri et al., 2017, Alluri et al., 2015, Burunat et al., 2018, Chapin et al., 2010a, Gagnepain et al., 2017) as well as in scalp EEG responses, for example to incongruous harmonic endings to phrases (James et al., 2008). These cross-sectional studies again may to some extent reflect pre-existing group differences in personality traits, socio-economic status or cognitive factors (Corrigall et al., 2013, Orsmond and Miller, 1999). In one longitudinal study, right hippocampal BOLD activity during music listening and imagery was positively correlated with success in a subsequent six-week piano training course; this marker of predisposition contrasted with cerebellar and fronto-parietal activity that increased over the course of that training (Herholz et al., 2016). In another study, tone patterns containing rhythmic deviants were presented to musicians before and after two semesters of university-level musical training, and to a control group of musicians receiving no such additional training (Herdener et al., 2010). Greater left anterior hippocampal responses to deviants occurred in the second session, only for the group receiving training. A positive relationship between musical aptitude and degree of activity in that same region was also found in cross-sectional analysis of a separate group. Taken together, the results suggest that at least some music-related differences in hippocampal function arise from training rather than innate ability.

The eye-catching finding that listening to a Mozart sonata improves spatial reasoning (Rauscher et al., 1993), an effect later established to be a rather non-specific consequence of arousal (Pietschnig et al., 2010, Thompson et al., 2001), led to interest in whether passive music exposure brings about hippocampal changes in animal models. Such exposure, especially prenatally or in development, can result in differential gene expression and regulation, elevated markers of neurogenesis, and changes in synaptic density and regulation in rodent (Angelucci et al., 2007, Chikahisa et al., 2006, Kim et al., 2006, Lee et al., 2016, Meng et al., 2009, Xing et al., 2016a) and avian (Chaudhury et al., 2010, Chaudhury et al., 2008, Chaudhury et al., 2006, Chaudhury and Wadhwa, 2009) hippocampus (but see Rizzolo et al., 2021). These effects are often accompanied by functional improvements, including in spatial (e.g. Xing et al., 2016a) and fear learning (e.g. Meng et al., 2009) tasks. Findings are mixed as to whether particular musical elements, such as rhythmic structure, versus more general auditory arousal, are responsible for the behavioral and neuronal changes (Angelucci et al., 2007, Chaudhury et al., 2008, Chaudhury et al., 2006, Field et al., 2007, Hu et al., 2014, Kirste et al., 2015, Sanyal et al., 2013a, Sanyal et al., 2013b, Xing et al., 2016b, Yang et al., 2014b, Yang et al., 2014a). Understanding how and why levels of the protein brain-derived neurotrophic factor increase through music exposure and engagement may be key to this question (Brattico et al., 2021).

In contrast to positive effects of music listening, noise exposure elevates hippocampal stress hormone levels (Barzegar et al., 2015, Britton et al., 1992, Campeau and Watson, 1997, Ferrarese et al., 1991, Gai et al., 2017, Jáuregui-Huerta et al., 2011, Jin et al., 2017) and markers of oxidative stress (Ambrosini et al., 2005, Cheng et al., 2016, Cheng et al., 2011, Manikandan et al., 2006, Uran et al., 2014), often resulting in accelerated cell death and reduced neurogenesis (Cui et al., 2009, Cui et al., 2013, Frenzilli et al., 2017, Gonzalez-Perez et al., 2011; B.-K. Kim et al., 2013; Kim et al., 2006; Kraus et al., 2010; Liu et al., 2010; Manikandan et al., 2006; Säljö et al., 2000, Säljö et al., 2002; Uran et al., 2012). Noise-induced reductions in NMDA receptors and related protein expression (Cui et al., 2013, Cui et al., 2009, Kapolowicz and Thompson, 2016, Singer et al., 2013) impair hippocampal long term potentiation (Barzegar et al., 2015, Cunha et al., 2018, Cunha et al., 2015, de Deus et al., 2017) and learning that depends on it (Barzegar et al., 2015, Cheng et al., 2011, Cui et al., 2009, Cui et al., 2012a, Cunha et al., 2015, Cunha et al., 2018, de Deus et al., 2017, de Deus et al., 2021, Di and Qin, 2018, Haider et al., 2012; B.-K. Kim et al., 2013; Kim et al., 2006; Manikandan et al., 2006; Uran et al., 2010, Uran et al., 2012, Uran et al., 2014). Spatial memory deficits may also result through destabilization of place cell receptive fields (Goble et al., 2009). A range of neurotransmitters and other markers of neural activity in the hippocampus are sensitive to noise exposure (Campeau and Watson, 1997, Cui et al., 2009, Cui et al., 2012a, de Deus et al., 2021, Di and Qin, 2018, Fernandes and File, 1993, Haider et al., 2012, Lai, 1987, Lai, 1988, Lai et al., 1989, Lai and Carino, 1990, Manikandan et al., 2006), as is DNA integrity (Frenzilli et al., 2017). Abnormalities in glial cells and levels of their activating proteins have also been described following noise presentation (Cui et al., 2015, Frenzilli et al., 2017, Huet-Bello et al., 2017) as have signatures of Alzheimer’s disease pathology, including tau hyperphosphorylation (Cheng et al., 2011, Cui et al., 2015, Cui et al., 2013, Cui et al., 2012a, Cui et al., 2012b, Frenzilli et al., 2017, Gai et al., 2017) and elevated amyloid-β and pro-inflammatory protein levels (Cui et al., 2015, Jafari et al., 2019).

Some of these changes depend on the duration (Barzegar et al., 2015, Cheng et al., 2011) or level (Hosseini-Sharifabad and Sabahi, 2008, Matt et al., 2018, Singer et al., 2013) of the noise, others can reverse over time (Cui et al., 2012a, Di and Qin, 2018, Frenzilli et al., 2017) or be protected against by various compounds (Abousetta et al., 2014, Alinaghipour et al., 2022, Azman et al., 2016, Li et al., 2014, Sundaramahalingam et al., 2013; S. Wang et al., 2016) or exercise (T.-W. Kim et al., 2013). Many are not specific to sound, arising due to a range of stressors (Chen et al., 2010; B.-K. Kim et al., 2013; Kim and Diamond, 2002). Given rodent work showing that rapid subcortical auditory pathways are more important for conveying noise than tone presence to hippocampus (Zhang et al., 2019), it will be important to establish whether such pathways also exist in humans to judge the clinical relevance of these findings. More in-depth reviews of the effects of noise on hippocampus are available elsewhere (Kraus and Canlon, 2012, Manukyan, 2022, Nadhimi and Llano, 2021, Zhang et al., 2022).

While noise exposure can cause deafness, most acquired hearing loss occurs gradually over the lifetime. Age-related hearing loss (presbycusis) is an independent risk factor for dementia, estimated to account for 9% of cases (Livingston et al., 2017). The hippocampus and entorhinal cortex are among the earliest sites showing dysfunction and atrophy in Alzheimer's disease (Braak and Braak, 1991, Khan et al., 2014), and poorer hearing in midlife is associated with steeper volumetric declines in these regions later in life (Armstrong et al., 2019). A large longitudinal study also found that individuals developing a hearing loss between scans an average of two years apart had greater decline in left hippocampal gray matter volume than those developing no such hearing loss (Fitzhugh and Pa, 2022). This group also showed a greater decrease in functional connectivity over time between auditory cortex and hippocampus (see also Andin, Holmer, 2022 for comparable results in individuals who are deaf from birth compared to controls). Connectivity in subjects with presbycusis is also disrupted between hippocampus and the inferior parietal lobule, to an extent that correlates with the degree of working memory impairment (Chen et al., 2020). The nature of any causal link between age-related hearing loss (or other listening impairments) and the hippocampal pathology and cognitive decline associated with dementia has not been fully established (Griffiths et al., 2020, Nadhimi and Llano, 2021, Tuwaig et al., 2017) but recent animal work offers some clues. The C57BL/6 mouse exhibits progressive hearing impairment and is widely used as a model for presbycusis. These animals, which also demonstrate impaired spatial behavior, suffer synaptic degeneration, decreased cell numbers and abnormal morphology in hippocampal CA1 and CA3 as well as altered neurotransmitter receptor expression (Beckmann et al., 2020, Dong et al., 2018, Yu et al., 2011). In studies with otherwise healthy mice, occluding ears to simulate conductive hearing loss also impairs hippocampal neurogenesis and increases microglial invasion and stress responses (Kurioka et al., 2021). Temporary conductive hearing loss can also be induced through ear drum perforation; in rats this interferes with NMDA receptor-mediated currents in CA1, reducing local field potentials and impairing spatial learning (Zhao et al., 2018). Finally, administration of ototoxic drugs to induce sensorineural hearing loss leads to hippocampal degeneration, impaired spatial learning, and tau phosphorylation (Y. Shen et al., 2021). Whether similar effects occur in humans is not yet known.

## 20 Synthesis and outstanding questions

We have reviewed imaging, recording, lesion and neuropsychological work across species, charting diverse interactions between sound and the hippocampus. These include a hierarchy of responses, from reacting non-selectively during passive listening, through tracking associations between particular sounds and rewards, to mapping out auditory dimensions during behavior. We identified hippocampal involvement in linking sounds with each other, with stimuli in other sensory modalities, and with spatiotemporal context to form episodic memories. We also detailed roles of the hippocampus in processing music, speech, emotional sound, and aberrant auditory percepts, as well as how hippocampal structure and function can be shaped by auditory experience. We described how the machinery that supports spatial navigation and memory may be harnessed in more general sequential processing, including that relating to sound. Questions remain as to the extent to which the hippocampus helps build representations of auditory objects and scenes, which are structured in time, frequency and space. However, the synthesis of auditory-hippocampal interaction that emerges does not support an adequate account of hippocampal function based on spatial navigation and episodic memory. The auditory work requires an explanation based on broader aspects of perception and cognition.

What does it mean to say the hippocampus is “involved in” a function? It may not be critical for a given task, but the stimulus and behavioral information it tracks becomes available either for an optimal solution to the current problem, or as part of a more complex representation than is available upstream and/or that may be drawn on subsequently. Although single-neuron tuning to specific auditory features is most pronounced when they are behaviorally relevant (Aronov et al., 2017, Itskov et al., 2012) we have also identified plenty of cases of hippocampal responses to sound during passive listening. Determining the critical dependency of certain auditory computations and functions on the hippocampus will require a combination of optogenetic techniques in animal models to temporarily shut down pathways, invasive and non-invasive stimulation in humans, and administering standardized batteries of auditory cognition (e.g. those previously applied to dementia patients, Hardy et al., 2020) to groups with particularly circumscribed hippocampal damage (e.g. autoimmune limbic encephalitis, Lad et al., 2019).

What characteristics of hippocampal circuits might lend themselves to auditory cognition? A non-exhaustive list could include: theta phase precession to track past, present and future in sound sequences; a monosynaptic pathway that can learn probabilistic contingencies between sounds and other stimuli; the means to separate patterns of signals arising from distinct auditory objects or in different contexts through sparseness and inhibition in dentate gyrus; the means to compare stored representations in CA3 with, or to complete, or predict from, current auditory input; connectivity with subcortical and cortical sites that provide auditory information processed with respect to distinct features or to different extents.

A number of steps can be taken to further test the idea that circuits in hippocampus and entorhinal cortex that support memory and navigation in physical space can be harnessed for auditory processing. Neuroimaging work that has indirectly found evidence for grid-cell like structures supporting non-spatial navigation (e.g. of semantic spaces) could be extended to cover auditory dimensions. Human intracranial recordings may provide more direct evidence for tuning to auditory features if coupled with the right task. Methodological advances in magnetic sensory technology, headcasts and source localization may facilitate temporally resolved magnetoencephalographic hippocampal recordings during listening (Alberto et al., 2021, Hill et al., 2020, Meyer et al., 2017, Pizzo et al., 2019, Recasens et al., 2018, Tierney et al., 2021).

An important outstanding question is the extent to which spatial coding and processing in the hippocampus dominates over other dimensions of experience. O'Keefe & Krupic (2021) argue that cells responding selectively to non-spatial stimuli are in fact feature-in-place cells, at least in non-humans; for humans they argue the spatial cognitive map has been enhanced by language and its metaphorical use. That is, responses that appear to be determined by stimulus characteristics are in fact primarily driven by the animal's spatial location - but this is masked because animals are not typically tested in different locations. This argument finds support in the Itskov et al. (2012) result that tuning to a particular sound only held when the animal's location was fixed. Also relevant is a finding that hippocampal responses during eyeblink trace conditioning only occurred in cells in whose place field the animal was situated, with conditioning leading to an arousal-related enhancement in firing (Shan et al., 2016). However, Mount et al. (2021) identified different hippocampal populations responding during acquisition versus extinction of trace conditioning. This suggests that arousal-modulated place tuning is unlikely alone to account for selective responses to particular sounds in individual cells.

Relatedly, is the hippocampus only engaged in forming auditory scenes or objects when there is an element of spatial variation rather than when spectral and/or temporal information alone are present? Experiments are needed in which subjects have to perceive, remember or mentally construct combinations of sounds composed of different feature conjunctions (such as a sound with a high amplitude modulation rate and low carrier frequency, followed by one with the reverse configuration). Comparable experiments with visual objects by Maguire and colleagues (Dalton et al., 2018, Zeidman et al., 2015) have been important in establishing the nature of hippocampal involvement in visual scene processing.

More straightforward analogs of experiments in other sensory modalities can answer pressing questions that have already been addressed for olfaction (in rodents) and vision (in humans and non-human primates), and that might be even more relevant for audition for which stimulation always unfolds over time. For example, does the phase at which rat hippocampal neurons fire for sequential sounds precess as the sequence unfolds (c.f. Terada et al., 2017)? Can calcium imaging reveal sparse encoding of behaviorally irrelevant auditory stimuli in mouse dentate gyrus (c.f. Woods et al., 2020)? Do sharp wave ripples in human hippocampus during auditory memory tasks reflect content-specific encoding, retrieval, replay and pre-play, in partnership with cortex (c.f. Norman et al., 2019)? Can predicted auditory content be decoded from human hippocampus and does this depend on stimulus complexity (c.f. Kok et al., 2020)? In addition, some proposed hippocampal coding schemes, based on spatiotemporal similarity (Turk-Browne, 2019), or modality/exemplar-invariant concepts (Quiroga et al., 2009) have not yet been tested against substantial auditory data.

We have seen how rhythmically structured sound at a range of timescales provides a non-invasive means of driving or entraining neural oscillations, which hippocampus plays a role in orchestrating across cortex. There are promising signs that such auditory interventions can improve memory and even disrupt pathology in animal models of disease. Compared to electrical, magnetic and invasive stimulation, an acoustic approach is accessible, inexpensive and generally non-intrusive/intimidating. To harness its full potential it will be important to understand how the auditory pathway and hippocampus work together with other regions including prefrontal cortex in bringing about these changes. More broadly, although we have focused on the hippocampus throughout this piece, considering such a richly connected structure in isolation will have only given part of the picture - whether with respect to conditioning, sound sequences, spatial and temporal aspects of sound, auditory memory, musical emotion, or phantom percepts.

If the findings from animal models concerning links between hippocampus, hearing loss, tinnitus and dementia are to translate into to clinical advances in humans it is important that we understand the degree of overlap between species in terms of anatomy, connectivity, physiology, and function. Recent results are reassuring in this regard. For example the identification of human hippocampal place cells, time cells, and phase precession - originally discovered in rodents - indicate that at least some of the coding and computational principles overlap. At the same time, post-mortem anatomical data on human auditory-hippocampal pathways is almost non-existent. We also acknowledge that different species evolved in the context of unique environmental constraints, with a range of sensory and higher-level capabilities (Basile et al., 2020). Despite these varied contexts under which the hippocampus operates, there are considerable areas of convergence in the auditory results we have reported. Another caveat is that we have surveyed literature linking sound and hippocampus as comprehensively as possible, but have not systematically reviewed each sub-topic in a meta-analytical manner, nor included every study in which no hippocampal involvement was reported. Findings should be interpreted accordingly and we hope that this review provides a launching point for further study.

In conclusion, the hippocampus receives all manner of auditory information regardless of its behavioral relevance at the time. Any tuning to acoustic features or criticality of involvement is strongest when there is a requirement to associate sounds with locations, rewards or punishments separated in time, other sounds, or stimuli in other sensory modalities - either for perception or memory. The structural, synaptic and biochemical components that facilitate such processing are themselves sensitive to the organism's auditory experience - both in the short and long-term.
