# Supplementary material for: The hearing hippocampus
Source: Prog Neurobiol. 2022 Nov;218:102326. doi: 10.1016/j.pneurobio.2022.102326 (PMC10510040; doi:10.1016/j.pneurobio.2022.102326)
Supplement: Supplementary file 1 — Supplementary material [file mmc1.docx]

**Table A. Connectivity between hippocampus/MTL and auditory areas in absence of an auditory stimulus**

*Anatomical tract tracing Diffusion tract tracing Electrical stimulation and physiology Electrical stimulation fMRI Optogenetic fMRI*

*Mouse* Cenquizca & Swanson (2007)^b^

*Rat* Deacon et al. (1983)^a^ Weitz et al. (2015)^b^

Swanson & Köhler (1986)^b^

Vaudano et al. (1991)^a,b^

Romanski & LeDoux (1993)^a^

Mascagni et al. (1993)^a^

Burwell & Amaral (1998)^a^

Linke (1999)^a­­^

Doron & LeDoux (2000)^a^

Burwell (2001)^a^

Kimura et al. (2003)^a^

Agster & Burwell (2009)^b^

Pereira et al. (2016)^a^

*Cat* Room & Groenewegen (1986a)^a^ Parmeggiani & Rapisada (1969)^b^

Room & Groenewegen (1986b)^b^

*Monkey* Jones & Powell (1970)^a^ Blatt et al. (2003)^a^ Rocchi, Oya et al. (2021)^a^

Van Hoesen et al. (1972)^a^

Seltzer & Pandya (1976)^a^

Turner et al. (1980)^a^

Amaral & Crown (1983)^a^

Insausti et al. (1987a)^a^

Insausti et al. (1987b)^a^

Tranel et al. (1988)^a,b^

Suzuki (1994)^a^

Blatt et al. (2003)^a^

Zhong et al. (2005)^a^

Munoz & Insausti (2005)^b^

*Human*  Baker et al. (2018)^c^ Catenoix et al. (2011)^b^ Rocchi, Oya et al. (2021)^a^

Maller et al. (2019)^c^ Enatsu et al. (2015)^b^

Jang & Choi (2022)^c^ Rocchi, Oya et al. (2021)^a,b^

^a^ From earlier auditory areas to hippocampus/MTL

^b^ From hippocampus/MTL to earlier auditory areas

^c^ Direction not determined

**Table B. Hippocampal stimulation with auditory responses elsewhere**

*Units LFP/iEEG*

*Rat* Ewing & Grace (2013)^a,b,c^

*Rabbit* Cazard & Buser (1963)^d^

*Cat* Fox et al. (1967)^e^ Fox et al. (1967)^e^

Parmeggiani et al. (1982)^f^ Redding (1967)^g^

Feldman & Dafny (1968)^h^

Parmeggiani & Rapisada (1969)^f^

^a^ Mediodorsal thalamic response

^b^ Nucleus accumbens response

^c^ Infralimbic cortical response

^d^ Motor cortical response

^e^ Cerebellar response

^f^ Auditory cortical response

^g^ Medial geniculate response

^h^ Hypothalamic response

**Table C. Hippocampal responses to simple sounds in the absence of an auditory task**

*Calcium imaging Intracellular Units LFP/iEEG EEG/MEG/PET/fMRI*

*Mouse* Kaifosh et al. (2013)^a^ Abe et al. (2014a)^a^ Okamoto et al. (2012)^b^ Stevens et al. (1996)^a^ Gurevicius et al. (2009)^a^

Abe et al. (2014b)^a^ Xiao et al. (2018)^a,c^ Stevens & Wear (1997)^a^ Okamoto et al. (2012)^b^

Wang et al. (2017)^a,b,c,i^ Zhang et al. (2018)^a,c,e^ Miller et al. (1999)^a^ Abe et al. (2014a)^a^

Martorell et al. (2019)^b^ Gurevicius et al. (2004)^a^

*Rat* Dietz & Manahan-V'n (2017)^a^ Olds & Hirano (1969)^a^ Pickenhain & Klingberg (1967)^b,e^ Miller & Freedman (1995)^a^

Hirano et al. (1970)^a^ Hall & Borbely (1970)^b^ Hershman et al. (1995)^a^

Olds et al. (1972)^a^ Irmiš et al. (1970)^a^ Bickford & Wear (1995)^a^

Segal & Olds (1972)^a^ Leaton & Buck (1971)^a^ Freedman et al. (1996)^a^

Segal (1973)^a^ Senba & Iwahara (1974)^a^ Stevens & Wear (1997)^a^

Segal (1974)^a^ Kramis et al. (1975)^a^ Adams & Stevens (1998)^a^

Mays & Best (1975)^a^ Deadwyler et al. (1981)^a^ Moxon et al. (1999)^b^

Miller & Groves (1977)^b^ Bland et al. (1981)^a^ Shinba (1999)^a^

Delacour (1980)^a^ Brankačk & Buzsáki (1986)^b^ Adams et al. (2000)^a^

Brankačk & Buzsáki (1986)^b^ Sainsbury et al. (1987)^a^ Bassant & Poindessous-Jazat (2001)^e^

Bickford-Wimer et al. (1990)^a^ Bickford-Wimer et al. (1990)^a^ Krause et al. (2003)^a^

Miller et al. (1992)^a^ Ehlers et al. (1991)^a^ Dissanayake et al. (2008)^a^

Bickford et al. (1993)^a^ Ehlers et al. (1991)^a^ Ma et al. (2009)^a^

Miller & Freedman (1993)^a^ Ehlers & Chaplin (1991)^a^ Dissanayake et al. (2009)^a^

Miller & Freedman (1995)^a^ Ehlers & Chaplin (1992)^a^ Hattori et al. (2010)^a^

Freedman et al. (1996)^a^ O'Connor et al. (1992)^b^ Ma & Leung (2011)^b^

Moxon et al. (1999)^b^ Jirsa et al. (1992)^a^ Klinkenberg et al. (2013)^b^

Shinba (1999)^a^ Miller et al. (1992)^a^ Gurevicius et al. (2013)^a^

Vinnik et al. (2012)^c^ Luntz-Leybman et al. (1992)^a^ Swerdlow et al. (2013)^b,c^

Ruusuvirta et al. (2013)^a^ Bickford et al. (1993)^a^ Long et al. (2014)^c^

Rothschild et al. (2017)^a^ Miller & Freedman (1993)^a^ Chen et al. (2014)^a,c^

Kaneko et al. (1993)^d,f^ Ruusuvirta et al. (2015)^a^

Ehlers et al. (1994)^a^ Rothschild et al. (2017) ^a,c^

Miller et al. (1995)^a^

*Rabbit* Green & Machne (1955)^b^ Jung & Kornmüller (1938)^g^ Bland et al. (1981)^a^

Vinogradova (1970)^a,b^ Green & Arduini (1954)^b,e^ Sinclair et al. (1982)^a^

Vinogradova et al. (1970)^a,b^ Green & Machne (1955)^b^ Whishaw & Dyck (1984)^a^

Livanov et al. (1971)^a,b^ Eidelberg et al. (1959) ^h^ Ruusuvirta et al. (1995a)^a^

Lidsky et al. (1974)^a^ Euler & Green (1960)^c^ Ruusuvirta et al. (1996)^a^

Lidsky et al. (1974)^a^ Ungiadze (1967)^b^ Vinogradova (2001)^a,b^

Sinclair et al. (1982)^a,e^  Lidsky et al. (1974)^a^ Astikainen et al. (2005)^a^

Vinogradova et al. (1993)^a,b,e^ Kramis et al. (1975)^a^ Ruusuvirta et al. (2010)^a^

Ruusuvirta et al. (1996)^a^ Martin et al. (1975)^e^

Vinogradova (2001)^a,b^ Whishaw (1976)^e^

*Guinea pig* Gao et al. (2010)^a^ Liberson & Cadilhac (1953)^g^ Pedemonte et al. (2001)^a^

Sainsbury & Montoya (1984)^a,c,e^ Liberman et al. (2009)^a^

*Phalanger* Adey et al. (1956)^a,b,e^

**Table C. Hippocampal responses to simple sounds in the absence of an auditory task (continued)**

*Calcium imaging Intracellular Units LFP/iEEG EEG/MEG/PET/fMRI*

*Bat* Yu & Moss (2022)^i,j^

*Cat* Brown & Buchwald (1973)^a,c^ Green & Arduini (1954)^b,e^ Başar et al. (1979a)^a^

Brown & Horn (1977) ^a,b^ Green & Adey (1956)^b^ Başar et al. (1979b)^a^

Grastyán et al. (1959)^b,h^ Csépe et al. (1989)^a^ Karmos et al. (1965)^g^ Başar-Eroglu et al. (1991)^a^

Herz (1965)^a^ Başar-Eroglu & Başar (1991)^a^

Radulovački & Adey (1965)^a^ Başar-Eroglu et al. (1991)^a^

Başar & Özesmi (1972)^f^ Demiralp et al. (1994)^a^

Başar & Ungan (1973)^f^ Ruusuvirta et al. (1995b)^a^

Başar et al. (1975a)^f^ Ruusuvirta et al. (1995c)^a^

Başar et al. (1975b)^f^ Demiralp et al. (1996)^a^

Kemp & Kaada (1975)^a^ Karashima et al. (2002)^a^

*Dog* Yoshii et al. (1966)^e,h^ Lucas et al. (1974)^a^

*Monkey* Desimone & Gross (1979)^e^ Gil-da-Costa et al. (2004)^e,j^

Tamura et al. (1990)^a,d,e,h,j^

Tamura et al. (1992)^a,b,d^

Paller et al. (1992)^a^

Sliwa et al. (2014)^g,h^

*Human* Derner et al. (2021)^a^ Brazier (1964)^b^ Boutros et al. (2005)^b^ Andreano et al. (2009)^g^

Halgren et al. (1980)^a^ Rosburg et al (2007)^a^ Mayer et al. (2009)^a^

Velasco et al. (1981)^b^  Boutros et al. (2008)^b^ Herdener et al. (2010)^a^

Velasco et al. (1982)^b^ Derner et al. (2018)^a^  Langers & Melcher (2011)^c^

Grunwald et al. (2003)^b^ Derner et al. (2020)^a^ Tobia et al. (2012)^a^

Yakunina et al. (2013)^c^

Barascud et al. (2016)^a^ Kok & Turk-Browne (2018)^a^

Kok et al. (2020)^a^

^a^ Pure tone

^b^ Click

^c^ Noise

^d^ Complex tone

^e^ Environmental/natural sound (inc. clap, whistle)

^f^ Square/saw-wave tone

^g^ Unspecified

^h^ Speech/voice

^i^ Frequency-modulated tone sweep

^j^ Conspecific call

**Table D. Hippocampal responses during conditioning**

*Calcium imaging Intracellular Units LFP/iEEG EEG/MEG /PET/fMRI*

*Mouse* Modi et al. (2014) Rummell et al. (2016) Desmedt et al. (1998)

Ahmed et al. (2020) Klee et al. (2021) Tang et al. (2003)

Mount et al. (2021) Rummell et al. (2016)

Klee et al. (2021)

*Rat* Shen et al. (2021) Olds & Hirano (1969) Laroche et al. (1987) Buzsáki et al. (1979) McHugh et al. (2013)

Hirano et al. (1970) Foster et al. (1987) Deadwyler et al. (1979a)

Olds et al. (1972) Foster et al. (1988) Deadwyler et al. (1979b)

Segal & Olds (1972) Edeline et al. (1988) Deadwyler et al. (1981)

Segal et al. (1972) Edeline et al. (1990) West et al. (1981)

Segal & Olds (1973) N'r-El Massioui et al. (1991) Bloch & Laroche (1981)

Segal (1973) Sakurai (1994) West et al. (1982)

Segal (1974) Moita et al. (2003) Deadwyler et al. (1985)

Best & Best (1976) Moita et al. (2004) Christan & Deadwyler (1986)

Disterhoft & Segal (1978) Gilmartin & McEchron (2005) Foster et al. (1988)

Deadwyler et al. (1979b) Oler et al. (2008) Jirsa et al. (1992)

West et al. (1981) Donzis et al. (2013) Talnov et al. (2003)

Bloch & Laroche (1981) Shan et al. (2016) Takehara-Nishiuchi et al. (2012)

Laroche et al. (1983) Rothschild et al. (2017) Sakimoto et al. (2013b)

Delacour (1984) Lee et al. (2017) Sakimoto et al. (2013a)

Hirano (1984) Wirtshafter & Wilson (2019) Sakimoto et al. (2013d)

Hirano & Yamaguchi (1985) Takamiya et al. (2021) Sakimoto et al. (2013c)

Sakimoto & Sakata (2013)

Sakimoto & Sakata (2014a)

Sakimoto & Sakata (2014b)

Sakimoto & Sakata (2015a)

Sakimoto & Sakata (2015b)

Sakimoto & Sakata (2015c)

Rothschild et al. (2017)

*Gerbil* Whishaw (1972)

*Rabbit* Disterhoft et al. (1986) Berger et al. (1976) Solomon et al. (1986) Berry & Thompson (1978)

Disterhoft et al. (1988) Berger & Thompson (1977) Port et al. (1987) Weisz et al. (1984)

LoTurco et al. (1988) Berger & Thompson (1978a) Stolar et al. (1989) Stolar et al. (1989)

Coulter et al. (1989) Berger & Thompson (1978b) Weiss et al. (1996) Salvatierra & Berry (1989)

De Jonge et al. (1990) Berry & Thompson (1978) Freeman et al. (1996) Nokia & Wikgren (2009)

Moyer et al. (1996) Hoehler & Thompson (1979) Freeman et al. (1997) Nokia et al. (2015)

Thompson et al. (1996) Hoehler & Thompson (1980) McEchron & Disterhoft (1997)

Berger et al. (1980) Ryou et al. (2001)

Berger & Thompson (1982) McEchron et al. (2001)

Berger et al. (1983) McEchron et al. (2003)

Weisz et al. (1984) Weible et al. (2006)

**Table D. Hippocampal responses during conditioning (continued)**

*Calcium imaging Intracellular Units LFP/iEEG EEG/MEG /PET/fMRI*

*Cat* Patterson et al. (1979) Maclean et al. (1955)

Múnera et al. (2001) Adey et al. (1960)

Radulovački & Adey (1965)

Elazar & Adey (1967)

Grastyán & Vereczkei (1974)

O'Connor & Starr (1985)

Múnera et al. (2001)

*Dog* Arnolds et al. (1979a)

Arnolds et al. (1979b)

*Human* Blaxton et al. (1996)

Schreurs et al. (1997)

Büchel et al. (1999)

Cheng et al. (2008)

Fujioka et al. (2010)

**Table E. Hippocampal responses to simple sounds during an auditory task (not during conditioning)**

*Calcium imaging Intracellular Units LFP/ iEEG EEG/MEG PET/fMRI*

*Rat* Sakurai (1990)^a^ Brankačk et al. (1996)^b^

Sakurai (1994)^a,b^ Shinba et al. (1996)^a^

Sakurai (1996)^b,c^ Givens (1996)^h^

Shinba (1999)^a^ Shinba (1999)^a^

Sakurai (2002)^a,d^ Onoda et al. (2003)^d^

Takahashi & Sakurai (2009)^a^ Hattori et al. (2010)^b^

Itskov et al. (2012)^e^ Shin (2011)^b^

Aronov et al. (2017)^f^ Laursen et al. (2014)^b^

Terada et al. (2017)^g^ Terada et al. (2017)^g^

*Guinea pig* Gao et al. (2010)^b^

*Human*  Wilson (1984)^i^ Halgren et al. (1980)^b^ Tesche et al. (1996)^b^ Yoshiura et al. (1999)^b^

Heit et al. (1990)^b,j^ Squires et al. (1983)^b^ Kikuchi et al. (1997)^b^ Crottaz-Herbette et al. (2005)^b^

Valasco et al. (1986) Nishitani et al. (1998)^b^ Schwenzer & Mathiak (2011)^b,s,t,u^

Smith et al. (1986)^b^ Tesche et al. (1999)^b^ Butler & James (2011)^v,w^

Altafullah et al. (1986)^b^ Alain et al. (2001)^q^ Liang et al. (2013)^x,y^

Meador et al. (1987)^b^ Takakura et al. (2003)^i,r^ Kumar et al. (2014)^z^

Meador et al. (1988)^b^ Recasens et al. (2018)^l^ Teki & Griffiths (2016)^A^

Puce et al. (1989)^b^ Kumar et al. (2016)^a^

McCarthy et al. (1989)^k^ Cousins et al. (2016)^B^

Smith et al. (1990)^b^ Danker et al. (2016)^C^ Heit et al. (1990)^b,j^ Jablonowski et al. (2018)^t^

Paller et al. (1992)^b^

Halgren et al. (1995)^b,j,l^

Kropotov et al. (2000)^b^

Roman et al. (2013)^i^

Becher et al. (2015)^m^

Barbeau et al. (2017)^n^

Curtu et al. (2019)^o^

Arnal et al. (2019)^p^

Kumar et al. (2021)^a^

^a^ Frequency working memory ^l^ Frequency repetition detection ^w^ Sound-image paired associate learning

^b^ Frequency discrimination ^m^ Monaural/binaural beat detection ^x^ Environmental sound discrimination

^c^ Configural frequency-light location discrimination ^n^ Human versus non-human sound discrimination ^y^ Word discrimination

^d^ Duration discrimination ^o^ Perceptual segregation reporting ^z^ Tone cloud repetition detection

^e^ Artificial vowel discrimination and response mapping ^p^ Sound aversion rating ^A^ Duration working memory and reproduction

^f^ Frequency reference memory and manipulation ^q^ Mistuned harmonic detection ^B^ Paired tone-squence and motor-sequence learning

^g^ Configural sound-odor discrimination and response mapping ^r^ Sound sequence order discrimination ^C^ Sound - written word associate learning

^h^ Auditory versus visual working memory ^s^ Frequency identification

^i^ Sound detection ^t^ Frequency contour identification

^j^ Omission detection ^u^ Sound localization

^k^ Intensity discrimination ^v^ Sound-sound paired associate learning

**Table F. Recording and functional imaging of human hippocampus - speech**

*Units LFP/iEEG PET/SPEC T/fMRI*

*Verbal memory* Gelbard-Sagiv et al. (2008) Babiloni et al. (2009) Grasby et al. (1993) Maguire & Frith (2004) Ren et al. (2018)

Paz et al. (2010) Babiloni et al. (2010) Dolan & Fletcher (1997) Breitenstein et al. (2005) Michelmann et al. (2021)

Urgolites et al. (2020) Henin et al. (2021) Kato et al. (1998) Peters et al. (2007) Gottlieb et al. (2010)

Urgolites et al. (2022) Saykin et al. (1999) Poppenk et al. (2008) Gagnepain et al. (2011)

Alkire et al. (1998) Davis et al. (2009) Persson et al. (2011)

Maguire & Mummery (1999) Svoboda & Levine (2009) Gottlieb et al. (2012)

Tulving et al. (1999) Straube et al. (2009) Bellace et al. (2012)

Petersson et al. (1999) Park & Rugg (2009) Kalm et al. (2013)

Small et al. (2001) Cuzzocreo et al. (2009) Dhanjal et al. (2013)

Habib et al. (2003) Lositsky et al. (2016) Thakral et al. (2015)

*Vocal emotion* Phillips et al. (1998) Alba-Ferrara et al. (2011) Kotz et al. (2013)

Paller et al. (2003) Musser et al. (2012) Beaucousin et al. (2006)

Mitchell et al. (2003)

*Speech perception/intelligibility* Michelmann et al. (2021) Davis & Johnsrude (2003) Davis et al. (2011) Blank et al. (2018)

Schmithorst et al. (2006) Manan et al. (2012)

Awad et al. (2007) Clos et al. (2014b)

*Self-generated speech* Awad et al. (2007) Van de Ven (2020)

*Target detection* Rocchi, Oya et al. (2021) Pollmann et al. (2004)

*Syntax* Meyer et al. (2005)

*Semantics* Meyer et al. (2005)

Piai et al. (2016)

Jafapour et al. (2017)

*Passive mismatch* Zevin & McCandliss (2005)

*Irrelevant/conflicting speech* Oehrn et al. (2015) Petersson et al. (2006) Oehrn et al. (2015)

*Cross-modal perception*  Joassin et al. (2011) Love et al. (2011)

*Concept/category selectivity* Quiroga et al. (2009)

Chan et al. (2011)

*Sequences and boundaries* Henin et al. (2021) Kalm et al. (2013)

Michelmann et al. (2021)

Ramos-Escobar et al. (2022)

*Voice familiarity* Abrams et al. (2016)

*Proverb familiarity* Gagnepain et al. (2017)

**Table G. Recording and functional imaging of human hippocampus - music**

*Units LFP/ iEEG EEG/MEG PET/SPEC T/fMRI*

*No task or only contrast versus silence* Brown et al. (2004) Mutschler et al. (2010)

Schmithorst (2005)

*Pleasure/pleasantness/liking* Blood & Zatorre (2001) Wilkins et al. (2014)

Koelsch et al. (2006) Mueller et al. (2015)

Koelsch et al. (2007) Cheung et al. (2019)

Salimpoor et al. (2013)

*Emotion* Baumgartner et al. (2006) Pereira et al. (2011)

Eldar et al. (2007) Trost et al. (2012)

Mitterschiffthaler et al. (2007) Koelsch et al. (2013)

Lerner et al. (2009) Skouras et al. (2014)

Mueller et al. (2011) Koelsch & Skouras (2014)

*Familiarity* Müller et al. (2013) Müller et al. (2013) Plailly et al. (2007) Gagnepain et al. (2017)

Groussard et al. (2010) Prabhakar et al. (2018)

Pereira et al. (2011) Kubit et al. (2018)

Karmonik et al. (2016)

*Melody recognition* Bonetti et al. (2020) Watanabe et al. (2008)

*Actual/imagined singing* Kleber et al. (2007)

*Tone detection* Lehne et al. (2014)

*Timbre/tonality deviant detection* Janata (2002)

*Preserved temporal order* Mueller et al. (2011) Mueller et al. (2015)

*Phrase boundary perception* Knösche et al. (2005)

*Harmonic congruity*  James et al. (2008) Cheung et al. (2019)

*Expressivity/spontaneity* Chapin et al. (2010) Engel & Keller (2011)

*Memory encoding* Bonetti et al. (2021)

*Episodic/autobiographic memory* Gelbard-Sagiv et al. (2008) Ford et al. (2011) Kubit et al. (2018)

Paz et al. (2010) Sekeres et al. (2018)

*Motif repetition* Burunat et al. (2014)

*Predictability*  Burunat et al. (2018) Cheung et al. (2019)

*Binding melody and lyrics*  Alonso et al. (2016)

**Table H. Auditory behaviour after manipulation of hippocampus**

*Orienting Acoustic startle Conditioning Auditory memory*

*Mouse* Park et al. (2002) Huerta et al. (2000) Balu et al. (2012) Zheng et al. (2022)

Ransome & Turnley (2008) Graves et al. (2002) Hanna et al. (2012)

Sun et al. (2010) Park et al. (2002) Kenney et al. (2012)

Pisansky et al. (2013) Schimanski et al. (2002) Wang et al. (2013)

Inta et al. (2014) Tseng et al. (2004) Raybuck & Lattal (2014)

Chowdhury et al. (2005) Madronal et al. (2016)

Misane et al. (2005) Madronal et al. (2016)

Rekart et al. (2005) Reichel et al. (2016)

Shen et al. (2006) Lee et al. (2016)

McOmish et al. (2008) Sellami et al. (2017)

McHugh & Tonegawa (2009) Wilmot et al. (2019)

Poulin et al. (2010) Twining et al. (2020)

Ricobaraza et al. (2012) Oh & Han (2020)

Gaisler-Salomon et al. (2012) Faucher et al. (2022)

*Rat* Niki (1967) Kemble & Ison (1971) Brady & Hunt (1955) Anagnostaras et al. (1999) Robinson et al. (2012) Meck et al. (1984)

Riddell et al. (1969) Coover & Levine (1972) Isaacson et al. (1961) McEchron et al. (2000) Fiorenza et al. (2012) Meck et al. (1987)

Hendrickson et al. (1969) Groves et al. (1974) Schwartzbaum et al. (1964) Shors et al. (2001) Tam & Bonardi (2012) Meck (1988)

Crowne & Riddell (1969) Leaton (1981) Swanson & Isaacson (1967) Beylin et al. (2001) DuPont et al. (2014) Olton et al. (1988)

Sanwald et al. (1970) Mickley & Ferguson (1989) Caul et al. (1969) Corcoran & Maren (2001) Lovett-Baron et al. (2014)

Köhler (1976) Caine et al. (1992) Barcik (1970) Quinn et al. (2002) Tam et al. (2015)

Honey et al. (1998) Lipska et al. (1995) Micco & Schwartz (1971) Talk et al. (2002) Wang et al. (2015)

Honey & Good (2000) Wan et al. (1996) Shinkman & Kaufman (1972a) Heldt et al. (2002) Lin et al. (2016)

Lee & Davis (1997a) Shinkman & Kaufman (1972b) Takehara et al. (2003) Fast et al. (2016)

Lee & Davis (1997b) Freeman et al. (1973) Bast et al. (2003) Talk et al. (2016)

Bakshi & Geyer (1998) Gustafson et al. (1975) Maren & Holt (2004) Miniaci et al. (2016)

Klarner et al. (1998) Rickert et al. (1978) Lee & Kesner et al. (2004) Riaz et al. (2017)

Daenen et al. (2003) Rickert et al. (1979) Quinn et al. (2005) Staib et al. (2018)

Shoemaker et al. (2005) Mastroianni (1979) Burman et al. (2006) Bazaz et al. (2022)

Fitting et al. (2006a) Rickert et al. (1981) Hobin et al. (2006) Asadian et al. (2022)

Fitting et al. (2006b) Ross et al. (1984) Bangasser et al. (2006)

Manahan-Vaughan et al. (2008) Winocur et al. (1987) Yoon & Otto (2007)

Ma & Leung (2011) Davidson & Jarrard (1989) Hunsaker & Kesner (2008)

Swerdlow et al. (2012) Jarrard & Davidson (1991) Quinn et al. (2008)

Swerdlow et al. (2013) Alvarado & Rudy (1995) Seo et al. (2008)

McDonald et al. (1997) Czerniawski et al. (2009)

Richmond et al. (1997) Esclassan et al. (2009)

McEchron et al. (1998) Iordanova et al. (2009)

Han et al. (1998) Pang et al. (2010)

Good et al. (1998) Lin & Honey (2011)

Roseler et al. (1998) Iordanova et al. (2011)

Sacchetti et al. (1999) Iordanova et al. (2011)

Maren (1999) Sierra-Mercado et al. (2011)

Holt & Maren (1999) Thomas & Tran (2012)

Holland et al. (1999) Czerniawski et al. (2012)

**Table H. Auditory behaviour after manipulation of hippocampus (continued)**

*Orienting Acoustic startle Conditioning Auditory memory*

*Rabbit* Schmaltz & Theios (1972) Port et al. (1987)

Solomon & Moore (1975) Loechner & Weisz (1987)

Solomon (1977) James et al. (1987)

Salafia et al. (1977) Salvatierra & Berry (1989)

Salafia et al. (1979) Akase et al. (1989)

Berger & Orr (1982) Rudy & Sutherland (1989)

Berger & Orr (1983) Moyer et al. (1990)

Port & Patterson (1984) Kim et al. (1995)

Solomon et al. (1986) Allen et al. (2002)

Port et al. (1986) Smith et al. (2004)

*Guinea pig* Wu et al. (2013)

*Bird* Gentle et al. (1978)

Oades (1980)

*Cat* Karmos et al. (1965) Brady et al. (1954) Karmos & Grastyán (1962)

Rogozea & Ungher (1968) Flynn & Wasman (1960)

*Dog*  Kowalska (1995)

Kowalska (1999)

*Monkey* Bagshaw et al. (1965) Stepien et al. (1960)

Fritz et al. (2005)

**Table I.** **Human hippocampal patients - tasks with simple auditory stimuli**

*Conditioning Memory Timing perception Audiospatial perception Contour perception* *Statistical learning*

*Resection* Daum et al. (1991) Stepien & Sierpinski (1960) Melgire et al. (2005) Tezer et al. (2012) Wickelgren (1968)

Milner & Teuber (1968)

Richards (1973)

Zatorre & Samson (1991)

Lancelot et al. (2003b)

Lancelot et al. (2003a)

Lancelot et al. (2005)

Borders et al. (2017)

*Epilepsy* Daum et al. (1989) Stepien & Sierpinski (1960) Ehrlé et al. (2001) Kotelenko et al. (2007) Han et al. (2011) Schapiro et al. (2014)

McGlinchey-Berroth et al. (1997) Samson et al. (2001) Tezer et al. (2012)

Han et al. (2011) Kotelenko et al. (2013)

Rabelo et al. (2015)

*Tumour* Luria & Karasseva (1968)

*Stroke* Chao & Knight (1995)

Borders et al. (2017)

*Hypoxia/anoxia* McGlinchey-Berroth et al. (1997) Bechara et al. (1995) Covington et al. (2018)

Borders et al. (2017)

*Encephalitis* McGlinchey-Berroth et al. (1997) Squire et al. (2001) Covington et al. (2018) Borders et al. (2017)

*Autoimmune encephalopathy* Borders et al. (2017)

*Traumatic brain injury* Borders et al. (2017)

**Table J. Human hippocampal patients - tasks with speech**

*Verbal memory Segmentation Emotion recognition Ambiguity detection Degraded speech Dichotic speech Referent processing Statistical learning*

*Resection* Scoville & Milner (1957) Lackner (1974) Adolphs et al. (2001) Mackay et al. (1998) Boatman et al. (2006)

Penfield & Milner (1958) Milner (1958)

Wickelgren (1968)

Milner & Teuber (1968)

Milner (1970)

Rausch & Crandall (1982)

McMillan et al. (1987)

Gabrieli et al. (1988)

Goldstein et al. (1988)

Frisk & Milner (1990a)

Frisk & Milner (1990b)

Samson & Zatorre (1991)

Samson & Zatorre (1992)

Helmstaedter & Elger (1996)

Helmstaedter et al. (1997)

Merhav et al. (2014)

Warren et al. (2016)

*Epilepsy* Meyer & Yates (1955) Bonora et al. (2011) Boatman et al. (2006) Han et al. (2011)

Rausch & Crandall (1982) Rajasekaran et al. (2014)

Helmstaedter et al. (1997)

O'Brien et al. (2003)

Dulay et al. (2004)

Mueller et al. (2012)

Witt et al. (2014)

Coras et al. (2014)

Grewe et al. (2020)

*Tumour* Luria & Karasseva (1968)

Cavazuti et al. (1980)

Jayakar et al. (2015)

*Leukoencephalopathy*  Ghika-Schmid et al. (1997)

*Encephalitis* Stefanacci et al. (2000) Kurcezk et al. (2013) Schapiro et al. (2014)

Barbeau et al. (2005) Covington et al. (2018)

Rubin et al. (2011)

Finke et al. (2012)

Warren & Duff (2014)

Merhav et al. (2014)

Hilverman et al. (2018)

**Table J. Human hippocampal patients - tasks with speech (continued)**

*Verbal memory Segmentation Emotion recognition Ambiguity detection Degraded speech Dichotic speech Referent processing Statistical learning*

*Hypoxia/anoxia* Gadian et al. (2000) Kurcezk et al. (2013) Covington et al. (2018)

Duff et al. (2006)

Gardiner et al. (2008)

Rubin et al. (2011)

Smith et al. (2014)

Warren & Duff (2014)

Merhav et al. (2014)

Hilverman et al. (2018)

*Stroke* Mayes et al. (2004)

Peters et al. (2007)

*Head injury* Duff et al. (2006)

*Alzheimer's disease* Boon et al. (2011)

**Table K. Human hippocampal patients - task with music**

*Melody discrimination/recognition Liking/pleasantness Timbre discimination Anisochrony discrimination Emotion recognition*

*Resection*  Zatorre (1985) Samson & Peretz (2005) Samson & Zatorre (1994) Samson et al. (2001) Gosselin et al. (2005) Samson & Zatorre (1991) Khalfa et al. (2008) Khalfa et al. (2008)

Samson & Zatorre (1992) Gosselin et al. (2011)

Samson & Peretz (2005)

*Epilepsy* Samson & Peretz (2005) Samson & Peretz (2005)

Papp et al. (2014)

*Frontotemporal lobar degeneration* Omar et al. (2011)

**Table L. Tinnitus**

*Structure Inactivation LFP EEG PET/SPECT fMRI*

*Rat* Singer et al. (2013) Chen et al. (2014) Chen et al. (2015)

*Guinea pig* Zhang et al. (2018)

Zhang et al. (2021)

*Human* Rey et al. (1984) De Ridder et al. (2006) Mohan et al. (2018) Shulman et al. (1995) Ueyama et al. (2013)

Kreyberg et al. (1992) Lockwood et al. (1998) Chen et al. (2017)

Corkin et al. (1997) Schecklmann et al. (2013) Lan et al. (2022)

Landgrebe et al. (2009) Simonetti et al. (2022)

Boyen et al. (2013)

Gunbey et al. (2015)

Tae et al. (2018)

**Table M. Auditory hallucinations**

*Structure PET/SPECT fMRI Electrical stimulation*

*Trait* Takebayashi et al. (2002) Musalek et al. (1989) Rotarska-Jagiela et al. (2010)

Suzuki et al. (2003) Copolov et al. (2003) Sommer et al. (2012)

Lahti et al. (2006) Shinn et al. (2013)

Clos et al. (2014a)

Li et al. (2017)

Lin et al. (2020)

Gao et al. (2020)

Hare et al. (2021)

*State* Silbersweig et al. (1995) Dierks et al. (1999) Kumar et al. (2022)

Liddle et al. (2000) Shergill et al. (2000)

Copolov et al. (2003) Lennox et al. (2000)

Shergill et al. (2001)

Sommer et al. (2012)

Clos et al. (2014a)

**Table N. Longer-term effects of sound exposure or absence**

*Noise Other detrimental Music Other beneficial*

*Mouse* Chen et al. (2010) Cheng et al. (2016) Yu et al. (2011) Chikahisa et al. (2006) Kirst et al. (2015)

Gonzalez-Perez et al. (2011) Liu et al. (2016) Busceti et al. (2015) Angelucci et al. (2007) Matt et al. (2018)

Cheng et al. (2011) Liu et al. (2018) Di & Qin (2018) Meng et al. (2009) Martorell et al. (2019)

Tao et al. (2015) Jafari et al. (2019) Dong et al. (2018) Kirst et al. (2015)

Wang et al. (2016) Beckmann et al. (2020) Mao et al. (2022)

Shen et al. (2021)

Kurioka et al. (2021)

*Rat* Lai (1987) Jáuregui-Huerta et al. (2011) Eraslan et al. (2015) Drago et al. (1996) Xing et al. (2016b) Chaudhury et al. (2009)

Lai (1988) Hawley & Leasure (2011) Barzegar et al. (2015) Hosseini & Sabahi (2008) Xing et al. (2016a) Chaudhury et al. (2009)

Lai et al. (1989) Cui et al. (2012a) Azman et al. (2016) Goble et al. (2009) Lee et al. (2016) Chaudhury et al. (2010)

Lai (1990) Cui et al. (2012b) Kapolowicz et al. (2016) Liu et al. (2010)

Ferrarese et al. (1991) Uran et al. (2012) De Deus et al. (2017) Yang et al. (2014a)

Britton et al. (1992) Haider et al. (2012) Gai et al. (2017) Yang et al. (2014b)

Fernandes & File (1993) Singer et al. (2013) Huet-Bello et al. (2017) Hu et al. (2014)

Campeau & Watson (1997) Cui et al. (2013) Frenzilli et al. (2017) Xing et al. (2016b)

Säljö et al. (2000) Kim et al. (2013) Jin et al. (2017) Cunha et al. (2018)

Säljö et al. (2002) Kim et al. (2013) Hayes et al. (2019) Zhao et al. (2018)

Kim et al. (2006) Sundaramahalingam et al. (2013) Cunha et al. (2019)

Manikandan et al. (2006) Li et al. (2014) Lee et al. (2020)

Cui et al. (2009) Uran et al. (2014) Zhang et al. (2021)

Kraus et al. (2010) Abousetta et al. (2014) De Deus et al. (2021)

Uran et al. (2010) Cui et al. (2015) Alinaghipour et al. (2022)

*Guinea pig* Zhang et al. (2018) Zhang et al. (2021)

*Bird* Sanyal et al. (2013b) Kathpalia et al. (2019) Chaudhury et al. (2006)

Sanyal et al. (2013a) Chaudhury et al. (2008)

Chaudhury et al. (2009)

Chaudhury & Wadhwa (2009)

Chaudhury et al. (2010)

Sanyal et al. (2013b)

Sanyal et al. (2013a)

Kathpalia et al. (2019)

*Human* Chapin et al. (2010) Ong et al. (2018)

Alluri et al. (2015)

Herholz et al. (2016)

Gagnepain et al. (2017)

Alluri et al. (2017)

Burunat et al. (2018)

**References**

Abe, R., Sakaguchi, T., Kitajo, K., Ishikawa, D., Matsumoto, N., Matsuki, N., Ikegaya, Y., 2014a. Sound-induced modulation of hippocampal θ oscillations: NeuroReport 25, 1368–1374. https://doi.org/10.1097/WNR.0000000000000274

Abe, R., Sakaguchi, T., Matsumoto, N., Matsuki, N., Ikegaya, Y., 2014b. Sound-induced hyperpolarization of hippocampal neurons: NeuroReport 25, 1013–1017. https://doi.org/10.1097/WNR.0000000000000206

Abousetta, A., Makhlouf, N.A., El-Beshbishy, R.A., 2014. The effects of concomitant Ginkgo intake on noise induced Hippocampus injury. Possible auditory clinical correlate. Egyptian Journal of Ear, Nose, Throat and Allied Sciences 15, 231–239. https://doi.org/10.1016/j.ejenta.2014.05.003

Abrams, D.A., Chen, T., Odriozola, P., Cheng, K.M., Baker, A.E., Padmanabhan, A., Ryali, S., Kochalka, J., Feinstein, C., Menon, V., 2016. Neural circuits underlying mother’s voice perception predict social communication abilities in children. Proceedings of the National Academy of Sciences 113, 6295–6300. https://doi.org/10.1073/pnas.1602948113

Adams, C.E., Stevens, K.E., 1998. Inhibition of Nitric Oxide Synthase Disrupts Inhibitory Gating of Auditory Responses in Rat Hippocampus 287, 6.

Adams, C.E., Stevens, K.E., Kem, W.R., Freedman, R., 2000. Inhibition of nitric oxide synthase prevents a7 nicotinic receptor- mediated restoration of inhibitory auditory gating in rat hippocampus. Brain Research 10.

Adey, W.R., Dunlop, C.W., Hendrix, C.E., 1960. Hippocampal Slow Waves: Distribution and Phase Relationships in the Course of Approach Learning. Archives of Neurology 3, 74. https://doi.org/10.1001/archneur.1960.00450010074007

Adey, W.R., Merrillees, N.C.R., Sunderland, S., 1956. The entorhinal area: Behavioural, evoked potential, and histological studies of its interrelationships with brain-stem regions 34.

Adolphs, R., Tranel, D., Damasio, H., 2001. Emotion recognition from faces and prosody following temporal lobectomy. Neuropsychology 15, 396–404. https://doi.org/10.1037//0894-4105.15.3.396

Agster, K.L., Burwell, R.D., 2009. Cortical efferents of the perirhinal, postrhinal, and entorhinal cortices of the rat. Hippocampus 19, 1159–1186. https://doi.org/10.1002/hipo.20578

Ahmed, M.S., Priestley, J.B., Castro, A., Stefanini, F., Solis Canales, A.S., Balough, E.M., Lavoie, E., Mazzucato, L., Fusi, S., Losonczy, A., 2020. Hippocampal Network Reorganization Underlies the Formation of a Temporal Association Memory. Neuron. https://doi.org/10.1016/j.neuron.2020.04.013

Akase, E., Alkon, D.L., Disterhoft, J.F., 1989. Hippocampal Lesions Impair Memory of Short-Delay Conditioned Eye Blink in Rabbits. Behavioral Neuroscience 103, 935–943.

Alain, C., Arnott, S.R., Picton, T.W., 2001. Bottom-up and top-down influences on auditory scene analysis: Evidence from event-related brain potentials. Journal of Experimental Psychology: Human Perception and Performance 27, 1071–1089.

Alba-Ferrara, L., Hausmann, M., Mitchell, R.L., Weis, S., 2011. The Neural Correlates of Emotional Prosody Comprehension: Disentangling Simple from Complex Emotion. PLoS ONE 6, e28701. https://doi.org/10.1371/journal.pone.0028701

Alinaghipour, A., Ashabi, G., Riahi, E., Soheili, M., Salami, M., Nabavizadeh, F., 2022. Effects of nano-curcumin on noise stress-induced hippocampus-dependent memory impairment: behavioral and electrophysiological aspects. Pharmacol. Rep. https://doi.org/10.1007/s43440-022-00354-3

Alkire, M.T., Haier, R.J., Fallon, J.H., Cahill, L., 1998. Hippocampal, but not amygdala, activity at encoding correlates with long-term, free recall of nonemotional information. Proceedings of the National Academy of Sciences 95, 14506–14510. https://doi.org/10.1073/pnas.95.24.14506

Allen, M.T., Padilla, Y., Myers, C.E., Gluck, M.A., 2002. Selective hippocampal lesions disrupt a novel cue effect but fail to eliminate blocking in rabbit eyeblink conditioning. Cognitive, Affective, & Behavioral Neuroscience 2, 318–328. https://doi.org/10.3758/CABN.2.4.318

Alluri, V., Brattico, E., Toiviainen, P., Burunat, I., Bogert, B., Numminen, J., Kliuchko, M., 2015. Musical expertise modulates functional connectivity of limbic regions during continuous music listening. Psychomusicology: Music, Mind, and Brain 25, 443–454. https://doi.org/10.1037/pmu0000124

Alluri, V., Toiviainen, P., Burunat, I., Kliuchko, M., Vuust, P., Brattico, E., 2017. Connectivity patterns during music listening: Evidence for action-based processing in musicians: Connectivity Patterns During Music Listening. Human Brain Mapping 38, 2955–2970. https://doi.org/10.1002/hbm.23565

Alonso, I., Davachi, L., Valabrègue, R., Lambrecq, V., Dupont, S., Samson, S., 2016. Neural correlates of binding lyrics and melodies for the encoding of new songs. NeuroImage 127, 333–345. https://doi.org/10.1016/j.neuroimage.2015.12.018

Altafullah, I., Halgren, E., Stapleton, J.M., Crandall, P.H., 1986. Interictal spike-wave complexes in the human medial temporal lobe: Typical topography and comparisons with cognitive potentials. Electroencephalography and Clinical Neurophysiology 63, 503–516.

Alvarado, M.C., Rudy, J.W., 1995. A Comparison of Kainic Acid Plus Colchicine and Ibotenic Acid-Induced Hippocampal Formation Damage on Four Configural Tasks in Rats. Behavioral Neuroscience 109, 1052–1062.

Amaral, D.G., Insausti, R., Cowan, W.M., 1983. Evidence for a direct projection from the superior temporal gyrus to the entorhinal cortex in the monkey. Brain Research 275, 263–277. https://doi.org/10.1016/0006-8993(83)90987-3

Anagnostaras, S.G., Maren, S., Fanselow, M.S., 1999. Temporally Graded Retrograde Amnesia of Contextual Fear after Hippocampal Damage in Rats: Within-Subjects Examination. J. Neurosci. 19, 1106–1114. https://doi.org/10.1523/JNEUROSCI.19-03-01106.1999

Andreano, J., Liang, K., Kong, L., Hubbard, D., Wiederhold, B.K., Wiederhold, M.D., 2009. Auditory Cues Increase the Hippocampal Response to Unimodal Virtual Reality. CyberPsychology & Behavior 12, 309–313. https://doi.org/10.1089/cpb.2009.0104

Angelucci, F., Fiore, M., Ricci, E., Padua, L., Sabino, A., Tonali, P.A., 2007. Investigating the neurobiology of music: brain-derived neurotrophic factor modulation in the hippocampus of young adult mice: Behavioural Pharmacology 18, 491–496. https://doi.org/10.1097/FBP.0b013e3282d28f50

Arnal, L.H., Kleinschmidt, A., Spinelli, L., Giraud, A.-L., Mégevand, P., 2019. The rough sound of salience enhances aversion through neural synchronisation. Nature Communications 10. https://doi.org/10.1038/s41467-019-11626-7

Arnolds, D.E.A.T., Lopes da Silva, F.H., Aitink, J.W., Kamp, A., 1979a. Hippocampal EEG and behaviour in dog. I. Hippocampal EEG correlates of gross motor behaviour. Electroencephalography and Clinical Neurophysiology 46, 552–570. https://doi.org/10.1016/0013-4694(79)90009-9

Arnolds, D.E.A.T., Lopes da Silva, F.H., Aitink, J.W., Kamp, A., 1979b. Hippocampal EEG and behaviour in dog. III. Hippocampal EEG correlates of stimulus-response tasks and of sexual behaviour. Electroencephalography and Clinical Neurophysiology 46, 581–591. https://doi.org/10.1016/0013-4694(79)90011-7

Aronov, D., Nevers, R., Tank, D.W., 2017. Mapping of a non-spatial dimension by the hippocampal–entorhinal circuit. Nature 543, 719–722. https://doi.org/10.1038/nature21692

Asadian, N., Parsaie, H., Vafaei, A.A., Dadkhah, M., Omoumi, S., Sedaghat, K., 2022. Chronic light deprivation induces different effects on spatial and fear memory and hippocampal BDNF/TRKB expression during light and dark phases of rat diurnal rhythm. Behavioural Brain Research 418, 113638. https://doi.org/10.1016/j.bbr.2021.113638

Astikainen, P., Ruusuvirta, T., Korhonen, T., 2005. Longer storage of auditory than of visual information in the rabbit brain: evidence from dorsal hippocampal electrophysiology. Experimental Brain Research 160, 189–193. https://doi.org/10.1007/s00221-004-1999-6

Awad, M., Warren, J.E., Scott, S.K., Turkheimer, F.E., Wise, R.J.S., 2007. A Common System for the Comprehension and Production of Narrative Speech. Journal of Neuroscience 27, 11455–11464. https://doi.org/10.1523/JNEUROSCI.5257-06.2007

Azman, K.F., Zakaria, R., Abdul Aziz, C.B., Othman, Z., 2016. Tualang Honey Attenuates Noise Stress-Induced Memory Deficits in Aged Rats. Oxidative Medicine and Cellular Longevity 2016, 1–11. https://doi.org/10.1155/2016/1549158

Babiloni, C., Vecchio, F., Mirabella, G., Buttiglione, M., Sebastiano, F., Picardi, A., Di Gennaro, G., Quarato, P.P., Grammaldo, L.G., Buffo, P., Esposito, V., Manfredi, M., Cantore, G., Eusebi, F., 2009. Hippocampal, amygdala, and neocortical synchronization of theta rhythms is related to an immediate recall during rey auditory verbal learning test. Human Brain Mapping 30, 2077–2089. https://doi.org/10.1002/hbm.20648

Babiloni, C., Vecchio, F., Mirabella, G., Sebastiano, F., Gennaro, G.D., Quarato, P.P., Buffo, P., Esposito, V., Manfredi, M., Cantore, G., Eusebi, F., 2010. Activity of hippocampal, amygdala, and neocortex during the Rey auditory verbal learning test: An event-related potential study in epileptic patients. Clinical Neurophysiology 121, 1351–1357. https://doi.org/10.1016/j.clinph.2010.02.160

Bagshaw, M.H., Kimble, D.P., Pribram, K.H., 1965. The GSR of monkeys during orienting and habituation and after ablation of the amygdala, hippocampus and inferotemporal cortex. Neuropsychologia 3, 111–119. https://doi.org/10.1016/0028-3932(65)90037-0

Baker, C.M., Burks, J.D., Briggs, R.G., Milton, C.K., Conner, A.K., Glenn, C.A., Sali, G., McCoy, T.M., Battiste, J.D., O’Donoghue, D.L., Sughrue, M.E., 2018. A Connectomic Atlas of the Human Cerebrum—Chapter 6: The Temporal Lobe. Operative Neurosurgery 15, S245–S294. https://doi.org/10.1093/ons/opy260

Bakshi, V.P., Geyer, M.A., 1998. Multiple Limbic Regions Mediate the Disruption of Prepulse Inhibition Produced in Rats by the Noncompetitive NMDA Antagonist Dizocilpine. J. Neurosci. 18, 8394–8401. https://doi.org/10.1523/JNEUROSCI.18-20-08394.1998

Balu, D.T., Carlson, G.C., Talbot, K., Kazi, H., Hill-Smith, T.E., Easton, R.M., Birnbaum, M.J., Lucki, I., 2012. Akt1 deficiency in schizophrenia and impairment of hippocampal plasticity and function. Hippocampus 22, 230–240. https://doi.org/10.1002/hipo.20887

Bangasser, D.A., Waxler, D.E., Santollo, J., Shors, T.J., 2006. Trace Conditioning and the Hippocampus: The Importance of Contiguity. Journal of Neuroscience 26, 8702–8706. https://doi.org/10.1523/JNEUROSCI.1742-06.2006

Barascud, N., Pearce, M.T., Griffiths, T.D., Friston, K.J., Chait, M., 2016. Brain responses in humans reveal ideal observer-like sensitivity to complex acoustic patterns. Proceedings of the National Academy of Sciences 113, E616–E625. https://doi.org/10.1073/pnas.1508523113

Barbeau, E.J., Chauvel, P., Moulin, C.J.A., Regis, J., Liégeois-Chauvel, C., 2017. Hippocampus duality: Memory and novelty detection are subserved by distinct mechanisms. Hippocampus 27, 405–416. https://doi.org/10.1002/hipo.22699

Barbeau, E.J., Felician, O., Joubert, S., Sontheimer, A., Ceccaldi, M., Poncet, M., 2005. Preserved visual recognition memory in an amnesic patient with hippocampal lesions. Hippocampus 15, 587–596. https://doi.org/10.1002/hipo.20079

Barcik, J.D., 1970. Hippocampal afterdischarges and conditioned emotional response. Psychonomic Science 20, 297–299. https://doi.org/10.3758/BF03329076

Barzegar, M., Sajjadi, F.S., Talaei, S.A., Hamidi, G., Salami, M., 2015. Prenatal exposure to noise stress: Anxiety, impaired spatial memory, and deteriorated hippocampal plasticity in postnatal life: PRENATAL SOUND STRESS AND COGNITION. Hippocampus 25, 187–196. https://doi.org/10.1002/hipo.22363

Başar, E., Demir, N., Gönder, A., Ungan, P., 1979a. Combined dynamics of EEG and evoked potentials I. Studies of simultaneously recorded EEG-EPograms in the auditory pathway, reticular formation, and hippocampus of the cat brain during the waking stage. Biological Cybernetics 34, 1–19.

Başar, E., Durusan, R., Gönder, A., Ungan, P., 1979b. Combined dynamics of EEG and evoked potentials II. Studies of simultaneously recorded EEG-EPograms in the auditory pathway, reticular formation, and hippocampus of the cat brain during sleep. Biological Cybernetics 34, 21–30.

Başar, E., Gönder, A., Özesmi, Ç., Ungan, P., 1975a. Dynamics of brain rhythmic and evoked potentials II. Studies in the auditory pathway, reticular formation, and hippocampus during the waking stage. Biological Cybernetics 20, 145–160.

Başar, E., Gönder, A., Özesmi, Ç., Ungan, P., 1975b. Dynamics of brain rhythmic and evoked potentials III. Studies in the auditory pathway, reticular formation, and hippocampus during sleep. Biological Cybernetics 20, 161–169.

Başar, E., Özesmi, Ç., 1972. The hippocampal EEG-activity and a systems analytical interpretation of averaged evoked potentials of the brain. Kybernetik 12, 45–54. https://doi.org/10.1007/BF00289236

Başar, E., Ungan, P., 1973. A component analysis and principles derived for the understanding of evoked potentials of the brain: Studies in the hippocampus. Kybernetik 12, 133–140. https://doi.org/10.1007/BF00289165

Başar-Eroglu, C., Başar, E., 1991. A Compound P300-40Hz Response of the Cat Hippocampus. International Journal of Neuroscience 60, 227–237. https://doi.org/10.3109/00207459109080642

Başar-Eroglu, C., Başar, E., Schmielau, F., 1991. P300 In Freely Moving Cats with Intracranial Electrodes. International Journal of Neuroscience 60, 215–226. https://doi.org/10.3109/00207459109080641

Başar-Eroglu, C, Schmielau, F., Schramm, U., Schult, J., 1991. P300 Response of Hippocampus Analyzed by Means of Multielectrodes in Cats. International Journal of Neuroscience 60, 239–248.

Bassant, M.-H., Poindessous-Jazat, F., 2001. Ventral tegmental nucleus of Gudden: A pontine hippocampal theta generator? Hippocampus 11, 809–813. https://doi.org/10.1002/hipo.1096

Bast, T., Zhang, W.-N., Feldon, J., 2003. Dorsal hippocampus and classical fear conditioning to tone and context in rats: Effects of local NMDA-receptor blockade and stimulation. Hippocampus 13, 657–675. https://doi.org/10.1002/hipo.10115

Baumgartner, T., Lutz, K., Schmidt, C.F., Jäncke, L., 2006. The emotional power of music: How music enhances the feeling of affective pictures. Brain Research 1075, 151–164. https://doi.org/10.1016/j.brainres.2005.12.065

Bazaz, A., Ghanbari, A., Vafaei, A.A., Khaleghian, A., Rashidy-Pour, A., 2022. Oxytocin in dorsal hippocampus facilitates auditory fear memory extinction in rats. Neuropharmacology 202, 108844. https://doi.org/10.1016/j.neuropharm.2021.108844

Beaucousin, V., Lacheret, A., Turbelin, M.-R., Morel, M., Mazoyer, B., Tzourio-Mazoyer, N., 2006. FMRI Study of Emotional Speech Comprehension. Cerebral Cortex 17, 339–352. https://doi.org/10.1093/cercor/bhj151

Bechara, A., Tranel, D., Damasio, H., Adolphs, R., Rockland, C., Damasio, A.R., 1995. Double Dissociation of Conditioning and Declarative Knowledge Relative to the Amygdala and Hippocampus in Humans 269, 5.

Becher, A.-K., Höhne, M., Axmacher, N., Chaieb, L., Elger, C.E., Fell, J., 2015. Intracranial electroencephalography power and phase synchronization changes during monaural and binaural beat stimulation. European Journal of Neuroscience 41, 254–263. https://doi.org/10.1111/ejn.12760

Beckmann, D., Feldmann, M., Shchyglo, O., Manahan-Vaughan, D., 2020. Hippocampal Synaptic Plasticity, Spatial Memory, and Neurotransmitter Receptor Expression Are Profoundly Altered by Gradual Loss of Hearing Ability. Cerebral Cortex. https://doi.org/10.1093/cercor/bhaa061

Bellace, M., Williams, J.M., Mohamed, F.B., Faro, S.H., 2012. An fMRI Study of the Activation of the Hippocampus by Emotional Memory. International Journal of Neuroscience 123, 121–127. https://doi.org/10.3109/00207454.2012.742894

Berger, T., Alger, B., Thompson, R., 1976. Neuronal substrate of classical conditioning in the hippocampus. Science 192, 483–485. https://doi.org/10.1126/science.1257783

Berger, T., Thompson, R., 1977. Limbic system interrelations: functional division among hippocampal-septal connections. Science 197, 587–589. https://doi.org/10.1126/science.560062

Berger, T.W., Laham, R.I., Thompson, R.F., 1980. Hippocampal unit-behavior correlations during classical conditioning. Brain Research 193, 229–248. https://doi.org/10.1016/0006-8993(80)90960-9

Berger, T.W., Orr, W.B., 1983. Hippocampectomy selectively disrupts discrimination reversal conditioning of the rabbit nictitating membrane response. Behavioural Brain Research 8, 49–68. https://doi.org/10.1016/0166-4328(83)90171-7

Berger, T.W., Orr, W.B., 1982. Role of the hippocampus in reversal learning of the rabbit nictitating membrane response, in: Woody (Ed.), Conditioning: Representation of Involved Neural Functions. p. 12.

Berger, T.W., Rinaldi, P.C., Weisz, D.J., Thompson, R.F., 1983. Single-unit analysis of different hippocampal cell types during classical conditioning of rabbit nictitating membrane response. Journal of Neurophysiology 50, 1197–1219. https://doi.org/10.1152/jn.1983.50.5.1197

Berger, T.W., Thompson, R.F., 1982. Hippocampal cellular plasticity during extinction of classically conditioned nictitating membrane behavior. Behavioural Brain Research 4, 63–76. https://doi.org/10.1016/0166-4328(82)90165-6

Berger, T.W., Thompson, R.F., 1978a. Neuronal plasticity in the limbic system during classical conditioning of the rabbit nictitating membrane response. I. The hippocampus. Brain Research 145, 323–346. https://doi.org/10.1016/0006-8993(78)90866-1

Berger, T.W., Thompson, R.F., 1978b. Identification of pyramidal cells as the critical elements in hippocampal neuronal plasticity during learning. Proceedings of the National Academy of Sciences 75, 1572–1576. https://doi.org/10.1073/pnas.75.3.1572

Berry, S., Thompson, R., 1978. Prediction of learning rate from the hippocampal electroencephalogram. Science 200, 1298–1300. https://doi.org/10.1126/science.663612

Best, M.R., Best, P.J., 1976. The effects of state of consciousness and latent inhibition on hippocampal unit activity in the rat during conditioning. Experimental Neurology 51, 564–573. https://doi.org/10.1016/0014-4886(76)90180-1

Beylin, A.V., Gandhi, C.C., Wood, G.E., Talk, A.C., Matzel, L.D., Shors, T.J., 2001. The Role of the Hippocampus in Trace Conditioning: Temporal Discontinuity or Task Difficulty? Neurobiology of Learning and Memory 76, 447–461. https://doi.org/10.1006/nlme.2001.4039

Bickford, P.C., Luntz-Leybman, V., Freedman, R., 1993. Auditory sensory gating in the rat hippocampus: modulation by brainstem activity. Brain Research 607, 33–38. https://doi.org/10.1016/0006-8993(93)91486-C

Bickford, P.C., Wear, K.D., 1995. Restoration of sensory gating of auditory evoked response by nicotine in fimbria-fornix lesioned rats. Brain Research 705, 235–240. https://doi.org/10.1016/0006-8993(95)01157-9

Bickford-Wimer, P.C., Nagamoto, H., Johnson, R., Adler, L.E., Egan, M., Rose, G.M., Freedman, R., 1990. Auditory sensory gating in hippocampal neurons: A model system in the rat. Biological Psychiatry 27, 183–192. https://doi.org/10.1016/0006-3223(90)90648-L

Bland, B.H., Sainsbury, R.S., Seto, M., Sinclair, B.R., Whishaw, I.Q., 1981. The use of sodium pentobarbital for the study of immobility-related (type 2) hippocampal theta. Physiology & Behavior 27, 363–368. https://doi.org/10.1016/0031-9384(81)90281-X

Blank, H., Spangenberg, M., Davis, M.H., 2018. Neural Prediction Errors Distinguish Perception and Misperception of Speech. J. Neurosci. 38, 6076–6089. https://doi.org/10.1523/JNEUROSCI.3258-17.2018

Blatt, G.J., Pandya, D.N., Rosene, D.L., 2003. Parcellation of cortical afferents to three distinct sectors in the parahippocampal gyrus of the rhesus monkey: An anatomical and neurophysiological study. The Journal of Comparative Neurology 466, 161–179. https://doi.org/10.1002/cne.10866

Blaxton, T.A., Zeffiro, T.A., Gabrieli, J.D.E., Bookheimer, S.Y., Carrillo, M.C., Theodore, W.H., Disterhoft, J.F., 1996. Functional Mapping of Human Learning: A Positron Emission Tomography Activation Study of Eyeblink Conditioning. The Journal of Neuroscience 16, 4032–4040. https://doi.org/10.1523/JNEUROSCI.16-12-04032.1996

Bloch, V., 1981. Conditioning of hippocampal cells: Its acceleration and long-term facilitation by post-trial reticular stimulation. Behavioural Brain Research 3, 23–42. https://doi.org/10.1016/0166-4328(81)90026-7

Blood, A.J., Zatorre, R.J., 2001. Intensely pleasurable responses to music correlate with activity in brain regions implicated in reward and emotion. Proceedings of the National Academy of Sciences 98, 11818–11823. https://doi.org/10.1073/pnas.191355898

Boatman, D.F., Lesser, R.P., Crone, N.E., Krauss, G., Lenz, F.A., Miglioretti, D.L., 2006. Speech Recognition Impairments in Patients with Intractable Right Temporal Lobe Epilepsy. Epilepsia 47, 1397–1401. https://doi.org/10.1111/j.1528-1167.2006.00562.x

Bonetti, L., Brattico, E., Carlomagno, F., Cabral, J., Stevner, A., Deco, G., Whybrow, P.C., Pearce, M., Pantazis, D., Vuust, P., Kringelbach, M.L., 2020. Spatiotemporal whole-brain dynamics of auditory patterns recognition (preprint). Neuroscience. https://doi.org/10.1101/2020.06.23.165191

Bonetti, L., Brattico, E., Carlomagno, F., Donati, G., Cabral, J., Haumann, N.T., Deco, G., Vuust, P., Kringelbach, M.L., 2021. Rapid encoding of musical tones discovered in whole-brain connectivity. NeuroImage 245, 118735. https://doi.org/10.1016/j.neuroimage.2021.118735

Bonora, A., Benuzzi, F., Monti, G., Mirandola, L., Pugnaghi, M., Nichelli, P., Meletti, S., 2011. Recognition of emotions from faces and voices in medial temporal lobe epilepsy. Epilepsy & Behavior 20, 648–654. https://doi.org/10.1016/j.yebeh.2011.01.027

Boon, M.E., Melis, R.J.F., Rikkert, M.O., Kessels, R.P.C., 2011. Atrophy in the Medial Temporal Lobe is Specifically Associated With Encoding and Storage of Verbal Information in MCI and Alzheimer Patients. Journal of Neurology Research. https://doi.org/10.4021/jnr18w

Borders, A.A., Aly, M., Parks, C.M., Yonelinas, A.P., 2017. The hippocampus is particularly important for building associations across stimulus domains. Neuropsychologia 99, 335–342. https://doi.org/10.1016/j.neuropsychologia.2017.03.032

Boutros, N.N., Mears, R., Pflieger, M.E., Moxon, K.A., Ludowig, E., Rosburg, T., 2008. Sensory gating in the human hippocampal and rhinal regions: Regional differences. Hippocampus 18, 310–316. https://doi.org/10.1002/hipo.20388

Boutros, N.N., Trautner, P., Rosburg, T., Korzyukov, O., Grunwald, T., Schaller, C., Elger, C.E., Kurthen, M., 2005. Sensory gating in the human hippocampal and rhinal regions. Clinical Neurophysiology 116, 1967–1974. https://doi.org/10.1016/j.clinph.2005.04.017

Boyen, K., Langers, D.R.M., de Kleine, E., van Dijk, P., 2013. Gray matter in the brain: Differences associated with tinnitus and hearing loss. Hearing Research 295, 67–78. https://doi.org/10.1016/j.heares.2012.02.010

Brady, J.V., Hunt, H.F., 1955. An Experimental Approach to the Analysis of Emotional Behavior. The Journal of Psychology 40, 313–324. https://doi.org/10.1080/00223980.1955.9712986

Brankačk, J., Buzsáki, G., 1986. Hippocampal responses evoked by tooth pulp and acoustic stimulation: Depth profiles and effect of behavior. Brain Research 378, 303–314. https://doi.org/10.1016/0006-8993(86)90933-9

Brankačk, J., Seidenbecher, T., M�ller-G�rtner, H.-W., 1996. Task-relevant late positive component in rats: Is it related to hippocampal theta rhythm? Hippocampus 6, 475–482. https://doi.org/10.1002/(SICI)1098-1063(1996)6:5<475::AID-HIPO1>3.0.CO;2-I

Brazier, M.A.B., 1964. Evoked responses recorded from the depths of the human brain. Annals of the New York Academy of Sciences 112, 33–59. https://doi.org/10.1111/j.1749-6632.1964.tb26741.x

Breitenstein, C., Jansen, A., Deppe, M., Foerster, A.-F., Sommer, J., Wolbers, T., Knecht, S., 2005. Hippocampus activity differentiates good from poor learners of a novel lexicon. NeuroImage 25, 958–968. https://doi.org/10.1016/j.neuroimage.2004.12.019

Britton, K.T., S. Segal, D., Kuczenski, R., Hauger, R., 1992. Dissociation between in vivo hippocampal norepinephrine response and behavioral/neuroendocrine responses to noise stress in rats. Brain Research 574, 125–130. https://doi.org/10.1016/0006-8993(92)90808-M

Brown, K.A., Buchwald, J.S., 1973. Acoustic responses and plasticity of limbic units in cats. Experimental Neurology 40, 608–631. https://doi.org/10.1016/0014-4886(73)90099-X

Brown, M.W., Horn, G., 1977. Responsiveness of neurones in the hippocampal region of anaesthetised and unanaesthetised cats to stimulation of sensory pathways. Brain Research 123, 241–259. https://doi.org/10.1016/0006-8993(77)90477-2

Brown, S., Martinez, M.J., Parsons, L.M., 2004. Passive music listening spontaneously engages limbic and paralimbic systems: NeuroReport 15, 2033–2037. https://doi.org/10.1097/00001756-200409150-00008

Büchel, C., Dolan, R.J., Armony, J.L., Friston, K.J., 1999. Amygdala–Hippocampal Involvement in Human Aversive Trace Conditioning Revealed through Event-Related Functional Magnetic Resonance Imaging. The Journal of Neuroscience 19, 10869–10876. https://doi.org/10.1523/JNEUROSCI.19-24-10869.1999

Burman, M.A., Starr, M.J., Gewirtz, J.C., 2006. Dissociable effects of hippocampus lesions on expression of fear and trace fear conditioning memories in rats. Hippocampus 16, 103–113. https://doi.org/10.1002/hipo.20137

Burunat, I., Alluri, V., Toiviainen, P., Numminen, J., Brattico, E., 2014. Dynamics of brain activity underlying working memory for music in a naturalistic condition. Cortex 57, 254–269. https://doi.org/10.1016/j.cortex.2014.04.012

Burunat, I., Brattico, E., Hartmann, M., Vuust, P., Särkämö, T., Toiviainen, P., 2018. Musical training predicts cerebello-hippocampal coupling during music listening. Psychomusicology: Music, Mind, and Brain 28, 152–163. https://doi.org/10.1037/pmu0000215

Burwell, R.D., 2001. Borders and cytoarchitecture of the perirhinal and postrhinal cortices in the rat. The Journal of Comparative Neurology 437, 17–41. https://doi.org/10.1002/cne.1267

Burwell, R.D., Amaral, D.G., 1998. Cortical afferents of the perirhinal, postrhinal, and entorhinal cortices of the rat. The Journal of Comparative Neurology 398, 179–205. https://doi.org/10.1002/(SICI)1096-9861(19980824)398:2<179::AID-CNE3>3.0.CO;2-Y

Busceti, C.L., Pietro, P.D., Riozzi, B., Traficante, A., Biagioni, F., Nisticò, R., Fornai, F., Battaglia, G., Nicoletti, F., Bruno, V., 2015. 5-HT2C serotonin receptor blockade prevents tau protein hyperphosphorylation and corrects the defect in hippocampal synaptic plasticity caused by a combination of environmental stressors in mice. Pharmacological Research 99, 258–268. https://doi.org/10.1016/j.phrs.2015.06.017

Butler, A.J., James, K.H., 2011. Cross-modal versus within-modal recall: Differences in behavioral and brain responses. Behavioural Brain Research. https://doi.org/10.1016/j.bbr.2011.06.017

Buzsáki, G., Grastyán, E., Tveritskaya, I.N., Czopf, J., 1979. Hippocampal evoked potentials and EEG changes during classical conditioning in the rat. Electroencephalography and Clinical Neurophysiology 47, 64–74. https://doi.org/10.1016/0013-4694(79)90033-6

Caine, S.B., Geyer, M.A., Swerdlow, N.R., 1992. Hippocampal modulation of acoustic startle and prepulse inhibition in the rat. Pharmacology Biochemistry and Behavior 43, 1201–1208. https://doi.org/10.1016/0091-3057(92)90503-8

Campeau, S., Watson, S.J., 1997. Neuroendocrine and Behavioral Responses and Brain Pattern of c-fos Induction Associated with Audiogenic Stress. Journal of Neuroendocrinology 9, 577–588. https://doi.org/10.1046/j.1365-2826.1997.00593.x

Catenoix, H., Magnin, M., Mauguière, F., Ryvlin, P., 2011. Evoked potential study of hippocampal efferent projections in the human brain. Clinical Neurophysiology 122, 2488–2497. https://doi.org/10.1016/j.clinph.2011.05.007

Caul, W.F., Jarrard, L.E., Miller, R.E., Korn, J.H., 1969. Effects of hippocampal lesions on heart rate in aversive classical conditioning. Physiology & Behavior 4, 917–922. https://doi.org/10.1016/0031-9384(69)90041-9

Cavazzuti, V., Winston, K., Baker, R., Welch, K., 1980. Psychological changes following surgery for tumors in the temporal lobe. Journal of Neurosurgery 53, 618–626. https://doi.org/10.3171/jns.1980.53.5.0618

Cazard, P., Buser, P., 1963. Modification des réponses sensorielles corticales par stimulation de l’hippocampe dorsal chez le lapin. Electroencephalography and Clinical Neurophysiology 15, 413–425.

Cenquizca, L.A., Swanson, L.W., 2007. Spatial organization of direct hippocampal field CA1 axonal projections to the rest of the cerebral cortex. Brain Research Reviews 56, 1–26. https://doi.org/10.1016/j.brainresrev.2007.05.002

Chan, A.M., Baker, J.M., Eskandar, E., Schomer, D., Ulbert, I., Marinkovic, K., Cash, S.S., Halgren, E., 2011. First-Pass Selectivity for Semantic Categories in Human Anteroventral Temporal Lobe. Journal of Neuroscience 31, 18119–18129. https://doi.org/10.1523/JNEUROSCI.3122-11.2011

Chao, L.L., Knight, R.T., 1995. Human prefrontal lesions increase distractibility to irrelevant sensory inputs. NeuroReport 6, 1605–1610.

Chapin, H., Jantzen, K., Scott Kelso, J.A., Steinberg, F., Large, E., 2010. Dynamic Emotional and Neural Responses to Music Depend on Performance Expression and Listener Experience. PLoS ONE 5, e13812. https://doi.org/10.1371/journal.pone.0013812

Chaudhury, S., Jain, S., Wadhwa, S., 2010. Expression of Synaptic Proteins in the Hippocampus and Spatial Learning in Chicks following Prenatal Auditory Stimulation. Developmental Neuroscience 32, 114–124. https://doi.org/10.1159/000279758

Chaudhury, S., Nag, T.C., Wadhwa, S., 2009. Effect of prenatal auditory stimulation on numerical synaptic density and mean synaptic height in the posthatch Day 1 chick hippocampus. Synapse 63, 152–159. https://doi.org/10.1002/syn.20585

Chaudhury, S., Nag, T.C., Wadhwa, S., 2008. Calbindin D-28K and parvalbumin expression in embryonic chick hippocampus is enhanced by prenatal auditory stimulation. Brain Research 1191, 96–106. https://doi.org/10.1016/j.brainres.2007.11.021

Chaudhury, S., Nag, T.C., Wadhwa, S., 2006. Prenatal acoustic stimulation influences neuronal size and the expression of calcium-binding proteins (calbindin D-28K and parvalbumin) in chick hippocampus. Journal of Chemical Neuroanatomy 32, 117–126. https://doi.org/10.1016/j.jchemneu.2006.07.002

Chaudhury, S., Wadhwa, S., 2009. Prenatal auditory stimulation alters the levels of CREB mRNA, p-CREB and BDNF expression in chick hippocampus. International Journal of Developmental Neuroscience 27, 583–590. https://doi.org/10.1016/j.ijdevneu.2009.06.004

Chen, G.-D., Radziwon, K.E., Kashanian, N., Manohar, S., Salvi, R., 2014. Salicylate-Induced Auditory Perceptual Disorders and Plastic Changes in Nonclassical Auditory Centers in Rats. Neural Plasticity 2014, 1–18. https://doi.org/10.1155/2014/658741

Chen, Y., Rex, C.S., Rice, C.J., Dube, C.M., Gall, C.M., Lynch, G., Baram, T.Z., 2010. Correlated memory defects and hippocampal dendritic spine loss after acute stress involve corticotropin-releasing hormone signaling. Proceedings of the National Academy of Sciences 107, 13123–13128. https://doi.org/10.1073/pnas.1003825107

Chen, Y.-C., Li, X., Liu, L., Wang, J., Lu, C.-Q., Yang, M., Jiao, Y., Zang, F.-C., Radziwon, K., Chen, G.-D., Sun, W., Krishnan Muthaiah, V.P., Salvi, R., Teng, G.-J., 2015. Tinnitus and hyperacusis involve hyperactivity and enhanced connectivity in auditory-limbic-arousal-cerebellar network. eLife 4. https://doi.org/10.7554/eLife.06576

Chen, Y.-C., Xia, W., Chen, H., Feng, Y., Xu, J.-J., Gu, J.-P., Salvi, R., Yin, X., 2017. Tinnitus distress is linked to enhanced resting-state functional connectivity from the limbic system to the auditory cortex: Altered Effective Connectivity in Tinnitus. Human Brain Mapping 38, 2384–2397. https://doi.org/10.1002/hbm.23525

Cheng, D.T., Disterhoft, J.F., Power, J.M., Ellis, D.A., Desmond, J.E., 2008. Neural substrates underlying human delay and trace eyeblink conditioning. Proceedings of the National Academy of Sciences 105, 8108–8113. https://doi.org/10.1073/pnas.0800374105

Cheng, L., Wang, S.-H., Chen, Q.-C., Liao, X.-M., 2011. Moderate noise induced cognition impairment of mice and its underlying mechanisms. Physiology & Behavior 104, 981–988. https://doi.org/10.1016/j.physbeh.2011.06.018

Cheng, L., Wang, S.-H., Huang, Y., Liao, X.-M., 2016. The hippocampus may be more susceptible to environmental noise than the auditory cortex. Hearing Research 333, 93–97. https://doi.org/10.1016/j.heares.2016.01.001

Cheung, V.K.M., Harrison, P.M.C., Meyer, L., Pearce, M.T., Haynes, J.-D., Koelsch, S., 2019. Uncertainty and Surprise Jointly Predict Musical Pleasure and Amygdala, Hippocampus, and Auditory Cortex Activity. Current Biology 29. https://doi.org/10.1016/j.cub.2019.09.067

Chikahisa, S., Sei, H., Morishima, M., Sano, A., Kitaoka, K., Nakaya, Y., Morita, Y., 2006. Exposure to music in the perinatal period enhances learning performance and alters BDNF/TrkB signaling in mice as adults. Behavioural Brain Research 169, 312–319. https://doi.org/10.1016/j.bbr.2006.01.021

Chowdhury, N., Quinn, J.J., Fanselow, M.S., 2005. Dorsal hippocampus involvement in trace fear conditioning with long, but not short, trace intervals in mice. Behavioral Neuroscience 119, 1396–1402. https://doi.org/10.1037/0735-7044.119.5.1396

Christian, E.P., Deadwyler, S.A., 1986. Behavioral functions and hippocampal cell types: evidence for two nonoverlapping populations in the rat. Journal of Neurophysiology 55, 331–348. https://doi.org/10.1152/jn.1986.55.2.331

Clos, M., Diederen, K.M.J., Meijering, A.L., Sommer, I.E., Eickhoff, S.B., 2014a. Aberrant connectivity of areas for decoding degraded speech in patients with auditory verbal hallucinations. Brain Structure and Function 219, 581–594. https://doi.org/10.1007/s00429-013-0519-5

Clos, M., Langner, R., Meyer, M., Oechslin, M.S., Zilles, K., Eickhoff, S.B., 2014b. Effects of prior information on decoding degraded speech: An fMRI study. Hum. Brain Mapp 35, 61–74. https://doi.org/10.1002/hbm.22151

Coover, G.D., Levine, S., 1972. Auditory startle response of hippocampectomized rats. Physiology & Behavior 9, 75–77. https://doi.org/10.1016/0031-9384(72)90268-5

Copolov, D.L., Seal, M.L., Maruff, P., Ulusoy, R., Wong, M.T.H., Tochon-Danguy, H.J., Egan, G.F., 2003. Cortical activation associated with the experience of auditory hallucinations and perception of human speech in schizophrenia: a PET correlation study. Psychiatry Research: Neuroimaging 122, 139–152. https://doi.org/10.1016/S0925-4927(02)00121-X

Coras, R., Pauli, E., Li, J., Schwarz, M., Rössler, K., Buchfelder, M., Hamer, H., Stefan, H., Blumcke, I., 2014. Differential influence of hippocampal subfields to memory formation: insights from patients with temporal lobe epilepsy. Brain 137, 1945–1957. https://doi.org/10.1093/brain/awu100

Corcoran, K.A., Maren, S., 2001. Hippocampal Inactivation Disrupts Contextual Retrieval of Fear Memory after Extinction. The Journal of Neuroscience 21, 1720–1726. https://doi.org/10.1523/JNEUROSCI.21-05-01720.2001

Corkin, S., Amaral, D.G., González, R.G., Johnson, K.A., Hyman, B.T., 1997. H. M.’s Medial Temporal Lobe Lesion: Findings from Magnetic Resonance Imaging. The Journal of Neuroscience 17, 3964–3979. https://doi.org/10.1523/JNEUROSCI.17-10-03964.1997

Coulter, D.A., Lo Turco, J.J., Kubota, M., Disterhoft, J.F., Moore, J.W., Alkon, D.L., 1989. Classical conditioning reduces amplitude and duration of calcium-dependent afterhyperpolarization in rabbit hippocampal pyramidal cells. Journal of Neurophysiology 61, 971–981. https://doi.org/10.1152/jn.1989.61.5.971

Cousins, J.N., El-Deredy, W., Parkes, L.M., Hennies, N., Lewis, P.A., 2016. Cued Reactivation of Motor Learning during Sleep Leads to Overnight Changes in Functional Brain Activity and Connectivity. PLoS Biol 14, e1002451. https://doi.org/10.1371/journal.pbio.1002451

Covington, N.V., Brown-Schmidt, S., Duff, M.C., 2018. The Necessity of the Hippocampus for Statistical Learning. Journal of Cognitive Neuroscience 30, 680–697. https://doi.org/10.1162/jocn_a_01228

Crottaz-Herbette, S., Lau, K.M., Glover, G.H., Menon, V., 2005. Hippocampal involvement in detection of deviant auditory and visual stimuli. Hippocampus 15, 132–139. https://doi.org/10.1002/hipo.20039

Crowne, D.P., Riddell, W.I., 1969. Hippocampal lesions and the cardiac component of the orienting response in the rat. Journal of Comparative and Physiological Psychology 69, 748–755. https://doi.org/10.1037/h0028172

Csépe, V., Karmos, G., Molnár, M., 1989. Subcortical Evoked Potential Correlates of Early Information Processing: Mismatch Negativity in Cats, in: Brain Dynamics: Progress and Perspectives. Springer, Berlin.

Cui, B., Li, K., Gai, Z., She, X., Zhang, N., Xu, C., Chen, X., An, G., Ma, Q., Wang, R., 2015. Chronic Noise Exposure Acts Cumulatively to Exacerbate Alzheimer’s Disease-Like Amyloid-β Pathology and Neuroinflammation in the Rat Hippocampus. Scientific Reports 5. https://doi.org/10.1038/srep12943

Cui, B., Wu, M., She, X., 2009. Effects of Chronic Noise Exposure on Spatial Learning and Memory of Rats in Relation to Neurotransmitters and NMDAR2B Alteration in the Hippocampus. Journal of Occupational Health 51, 152–158. https://doi.org/10.1539/joh.L8084

Cui, B., Wu, M., She, X., Liu, H., 2012a. Impulse noise exposure in rats causes cognitive deficits and changes in hippocampal neurotransmitter signaling and tau phosphorylation. Brain Research 1427, 35–43. https://doi.org/10.1016/j.brainres.2011.08.035

Cui, B., Wu, M.Q., Zhu, L.X., She, X.J., Qiang, M.A., Liu, H.T., 2013. Effect of chronic noise exposure on expression of N-Methyl-D-Aspartic acid receptor 2B and tau phosphorylation in hippocampus of rats. Biomedical Environmental Science 26, 163–168.

Cui, B., Zhu, L., She, X., Wu, M., Ma, Q., Wang, T., Zhang, N., Xu, C., Chen, X., An, G., Liu, H., 2012b. Chronic noise exposure causes persistence of tau hyperphosphorylation and formation of NFT tau in the rat hippocampus and prefrontal cortex. Experimental Neurology 238, 122–129. https://doi.org/10.1016/j.expneurol.2012.08.028

Cunha, A.O.S., Ceballos, C.C., de Deus, J.L., Leão, R.M., 2018. Long-term high-intensity sound stimulation inhibits h current ( *I* _h_ ) in CA1 pyramidal neurons. European Journal of Neuroscience 47, 1401–1413. https://doi.org/10.1111/ejn.13954

Cunha, A.O.S., de Deus, J.L., Ceballos, C.C., Leão, R.M., 2019. Increased hippocampal GABAergic inhibition after long-term high-intensity sound exposure. PLOS ONE 14, e0210451. https://doi.org/10.1371/journal.pone.0210451

Curtu, R., Wang, X., Brunton, B.W., Nourski, K.V., 2019. Neural Signatures of Auditory Perceptual Bistability Revealed by Large-Scale Human Intracranial Recordings. J. Neurosci. 39, 6482–6497. https://doi.org/10.1523/JNEUROSCI.0655-18.2019

Cuzzocreo, J.L., Yassa, M.A., Verduzco, G., Honeycutt, N.A., Scott, D.J., Bassett, S.S., 2009. Effect of handedness on fMRI activation in the medial temporal lobe during an auditory verbal memory task. Human Brain Mapping 30, 1271–1278. https://doi.org/10.1002/hbm.20596

Czerniawski, J., Ree, F., Chia, C., Otto, T., 2012. Dorsal versus ventral hippocampal contributions to trace and contextual conditioning: Differential effects of regionally selective nmda receptor antagonism on acquisition and expression. Hippocampus 22, 1528–1539. https://doi.org/10.1002/hipo.20992

Czerniawski, J., Yoon, T., Otto, T., 2009. Dissociating space and trace in dorsal and ventral hippocampus. Hippocampus 19, 20–32. https://doi.org/10.1002/hipo.20469

Daenen, E.W.P.M., Wolterink, G., Van Der Heyden, J.A., Kruse, C.G., Van Ree, J.M., 2003. Neonatal lesions in the amygdala or ventral hippocampus disrupt prepulse inhibition of the acoustic startle response; implications for an animal model of neurodevelopmental disorders like schizophrenia. European Neuropsychopharmacology 13, 187–197. https://doi.org/10.1016/S0924-977X(03)00007-5

Danker, J.F., Tompary, A., Davachi, L., 2016. Trial-by-Trial Hippocampal Encoding Activation Predicts the Fidelity of Cortical Reinstatement During Subsequent Retrieval. Cerebral Cortex bhw146. https://doi.org/10.1093/cercor/bhw146

Daum, I., Channon, S., Canavan, A.G., 1989. Classical conditioning in patients with severe memory problems. Journal of Neurology, Neurosurgery & Psychiatry 52, 47–51. https://doi.org/10.1136/jnnp.52.1.47

Daum, I., Channon, S., Polkey, C.E., Gray, J.A., 1991. Classical Conditioning After Temporal Lobe Lesions in Man: Impairment in Conditional Discrimination. Behavioral Neuroscience 105, 396–408.

Davidson, T.L., Jarrard, L.E., 1989. Retention of concurrent conditional discriminations in rats with ibotenate lesions of hippocampus. Psychobiology 17, 49–60.

Davis, M.H., Di Betta, A.M., Macdonald, M.J.E., Gaskell, M.G., 2009. Learning and Consolidation of Novel Spoken Words. Journal of Cognitive Neuroscience 21, 803–820. https://doi.org/10.1162/jocn.2009.21059

Davis, M.H., Ford, M.A., Kherif, F., Johnsrude, I.S., 2011. Does Semantic Context Benefit Speech Understanding through “Top–Down” Processes? Evidence from Time-resolved Sparse fMRI. Journal of Cognitive Neuroscience 23, 3914–3932. https://doi.org/10.1162/jocn_a_00084

Davis, M.H., Johnsrude, I.S., 2003. Hierarchical Processing in Spoken Language Comprehension. The Journal of Neuroscience 23, 3423–3431. https://doi.org/10.1523/JNEUROSCI.23-08-03423.2003

de Deus, J.L., Amorim, M.R., Ribeiro, A.B., Barcellos-Filho, P.C.G., Ceballos, C.C., Branco, L.G.S., Cunha, A.O.S., Leão, R.M., 2021. Loss of Brain-Derived Neurotrophic Factor Mediates Inhibition of Hippocampal Long-Term Potentiation by High-Intensity Sound. Cell Mol Neurobiol 41, 751–763. https://doi.org/10.1007/s10571-020-00881-8

de Deus, J.L., Cunha, A.O.S., Terzian, A.L., Resstel, L.B., Elias, L.L.K., Antunes-Rodrigues, J., Almeida, S.S., Leão, R.M., 2017. A single episode of high intensity sound inhibits long-term potentiation in the hippocampus of rats. Scientific Reports 7. https://doi.org/10.1038/s41598-017-14624-1

de Jonge, M.C., Black, J., Deyo, R.A., Disterhoft, J.F., 1990. Learning-induced afterhyperpolarization reductions in hippocampus are specific for cell type and potassium conductance. Exp Brain Res 80, 456–462. https://doi.org/10.1007/BF00227987

De Ridder, D., Fransen, H., Francois, O., Sunaert, S., Kovacs, S., Van De Heyning, P., 2006. Amygdalohippocampal involvement in tinnitus and auditory memory. Acta Oto-Laryngologica 126, 50–53. https://doi.org/10.1080/03655230600895580

Deacon, T.W., Eichenbaum, H., Rosenberg, P., Eckmann, K.W., 1983. Afferent connections of the perirhinal cortex in the rat. The Journal of Comparative Neurology 220, 168–190. https://doi.org/10.1002/cne.902200205

Deadwyler, S., West, M., Robinson, J., 1981. Entorhinal and septal inputs differentially control sensory-evoked responses in the rat dentate gyrus. Science 211, 1181–1183. https://doi.org/10.1126/science.7466392

Deadwyler, S.A., West, M., Lynch, G., 1979a. Synaptically identified hippocampal slow potentials during behavior. Brain Research 161, 211–225. https://doi.org/10.1016/0006-8993(79)90064-7

Deadwyler, S.A., West, M., Lynch, G., 1979b. Activity of dentate granule cells during learning: Differentiation of perforant path input. Brain Research 169, 29–43. https://doi.org/10.1016/0006-8993(79)90371-8

Deadwyler, S.A., West, M.O., Christian, E.P., Hampson, R.E., Foster, T.C., 1985. Sequence-related changes in sensory-evoked potentials in the dentate gyrus: A mechanism for item-specific short-term information storage in the hippocampus. Behavioral and Neural Biology 44, 201–212. https://doi.org/10.1016/S0163-1047(85)90198-0

Delacour, J., 1984. Two neuronal systems are involved in a classical conditioning in the rat. Neuroscience 13, 705–715. https://doi.org/10.1016/0306-4522(84)90090-3

Delacour, J., 1980. Conditioned modifications of arousal and unit activity in the rat hippocampus. Experimental Brain Research 38. https://doi.org/10.1007/BF00237935

Demiralp, T., Başar-Eroglu, C., Başar, E., 1996. Distributed Gamma Band Responses in the Brain Studied in Cortex, Reticular Formation, Hippocampus and Cerebellum. International Journal of Neuroscience 84, 1–13. https://doi.org/10.3109/00207459608987246

Demiralp, T., Başar-Eroglu, C., Rahn, E., Başar, E., 1994. Event-related theta rhythms in cat hippocampus and prefrontal cortex during an omitted stimulus paradigm. International Journal of Psychophysiology 18, 35–48. https://doi.org/10.1016/0167-8760(84)90013-8

Derner, M., Chaieb, L., Dehnen, G., Reber, T.P., Borger, V., Surges, R., Staresina, B.P., Mormann, F., Fell, J., 2021. Auditory Beat Stimulation Modulates Memory-Related Single-Neuron Activity in the Human Medial Temporal Lobe. Brain Sciences 11, 364. https://doi.org/10.3390/brainsci11030364

Derner, M., Chaieb, L., Surges, R., Staresina, B.P., Fell, J., 2018. Modulation of Item and Source Memory by Auditory Beat Stimulation: A Pilot Study With Intracranial EEG. Frontiers in Human Neuroscience 12. https://doi.org/10.3389/fnhum.2018.00500

Derner, M., Dehnen, G., Chaieb, L., Reber, T.P., Borger, V., Surges, R., Staresina, B.P., Mormann, F., Fell, J., 2020. Patterns of single-neuron activity during associative recognition memory in the human medial temporal lobe. NeuroImage 221, 117214. https://doi.org/10.1016/j.neuroimage.2020.117214

Desimone, R., Gross, C.G., 1979. Visual areas in the temporal cortex of the macaque. Brain Research 178, 363–380. https://doi.org/10.1016/0006-8993(79)90699-1

Desmedt, A., Garcia, R., Jaffard, R., 1998. Differential Modulation of Changes in Hippocampal–Septal Synaptic Excitability by the Amygdala as a Function of Either Elemental or Contextual Fear Conditioning in Mice. The Journal of Neuroscience 18, 480–487. https://doi.org/10.1523/JNEUROSCI.18-01-00480.1998

Dhanjal, N.S., Warren, J.E., Patel, M.C., Wise, R.J.S., 2013. Auditory cortical function during verbal episodic memory encoding in Alzheimer’s disease: Auditory Cortical Function. Annals of Neurology 73, 294–302. https://doi.org/10.1002/ana.23789

Di, G.-Q., Qin, Z.-Q., 2018. Influences of combined traffic noise on the ability of learning and memory in mice. Noise Health 20, 9–15.

Dierks, T., Linden, D.E.J., Jandl, M., Formisano, E., Goebel, R., Lanfermann, H., Singer, W., 1999. Activation of Heschl’s Gyrus during Auditory Hallucinations. Neuron 22, 615–621. https://doi.org/10.1016/S0896-6273(00)80715-1

Dietz, B., Manahan-Vaughan, D., 2017. Hippocampal long-term depression is facilitated by the acquisition and updating of memory of spatial auditory content and requires mGlu5 activation. Neuropharmacology 115, 30–41. https://doi.org/10.1016/j.neuropharm.2016.02.026

Dissanayake, D.W.N., Zachariou, M., Marsden, C.A., Mason, R., 2009. Effects of phencyclidine on auditory gating in the rat hippocampus and the medial prefrontal cortex. Brain Research 1298, 153–160. https://doi.org/10.1016/j.brainres.2009.08.032

Dissanayake, D.W.N., Zachariou, M., Marsden, C.A., Mason, R., 2008. Auditory gating in rat hippocampus and medial prefrontal cortex: Effect of the cannabinoid agonist WIN55,212-2. Neuropharmacology 55, 1397–1404. https://doi.org/10.1016/j.neuropharm.2008.08.039

Disterhoft, J.F., Coulter, D.A., Alkon, D.L., 1986. Conditioning-specific membrane changes of rabbit hippocampal neurons measured in vitro. Proceedings of the National Academy of Sciences 83, 2733–2737. https://doi.org/10.1073/pnas.83.8.2733

Disterhoft, J.F., Golden, D.T., Read, H.L., Coulter, D.A., Alkon, D.L., 1988. AHP reductions in rabbit hippocampal neurons during conditioning correlate with acquisition of the learned response. Brain Research 462, 118–125. https://doi.org/10.1016/0006-8993(88)90593-8

Disterhoft, J.F., Segal, M., 1978. Neuron activity in rat hippocampus and motor cortex during discrimination reversal. Brain Research Bulletin 3, 583–588. https://doi.org/10.1016/0361-9230(78)90003-5

Dolan, R.J., Fletcher, P.C., 1997. Dissociating prefrontal and hippocampal function in episodic memory encoding. Nature 388, 582–585. https://doi.org/10.1038/41561

Dong, Y., Guo, C.-R., Chen, D., Chen, S.-M., Peng, Y., Song, H., Shi, J.-R., 2018. Association between age‑related hearing loss and cognitive decline in C57BL/6J mice. Molecular Medicine Reports 18, 1726–1732. https://doi.org/10.3892/mmr.2018.9118

Donzis, E.J., Rennaker, R.L., Thompson, L.T., 2013. Fear conditioning alters neuron-specific hippocampal place field stability via the basolateral amygdala. Brain Research 1525, 16–25. https://doi.org/10.1016/j.brainres.2013.06.015

Doron, N.N., Ledoux, J.E., 2000. Cells in the posterior thalamus project to both amygdala and temporal cortex: A quantitative retrograde double-labeling study in the rat. The Journal of Comparative Neurology 425, 257–274. https://doi.org/10.1002/1096-9861(20000918)425:2<257::AID-CNE8>3.0.CO;2-Y

Drago, F., Musco, S., Nardo, L., Valerio, C., Grassi, M., Rampello, L., 1996. Behavioral and neurochemical alterations induced by reversible conductive hearing loss in aged male rats. Neuroscience Letters 205, 1–4. https://doi.org/10.1016/0304-3940(96)12369-7

Duff, M.C., Hengst, J., Tranel, D., Cohen, N.J., 2006. Development of shared information in communication despite hippocampal amnesia. Nature Neuroscience 9, 140–146. https://doi.org/10.1038/nn1601

Dulay, M.F., Schefft, B.K., Fargo, J.D., Privitera, M.D., Yeh, H., 2004. Severity of depressive symptoms, hippocampal sclerosis, auditory memory, and side of seizure focus in temporal lobe epilepsy. Epilepsy & Behavior 5, 522–531. https://doi.org/10.1016/j.yebeh.2004.04.007

DuPont, C.M., Coppola, J.J., Kaercher, R.M., Lindquist, D.H., 2014. Impaired trace fear conditioning and diminished ERK1/2 phosphorylation in the dorsal hippocampus of adult rats administered alcohol as neonates. Behavioral Neuroscience 128, 187–198. https://doi.org/10.1037/a0035989

Edeline, J.-M., Dutrieux, G., Neuenschwander-El Massioui, N., 1988. Multiunit changes in hippocampus and medial geniculate body in free-behaving rats during acquisition and retention of a conditioned response to a tone. Behavioral and Neural Biology 50, 61–79. https://doi.org/10.1016/S0163-1047(88)90780-7

Edeline, J.-M., Neuenschwander-El Massioui, N., Dutrieux, G., 1990. Discriminative long-term retention of rapidly induced multiunit changes in the hippocampus, medial geniculate and auditory cortex. Behavioural Brain Research 39, 145–155. https://doi.org/10.1016/0166-4328(90)90101-J

Ehlers, C.L., Chaplin, R.I., 1992. Long latency event related potentials in rats: The effects of changes in stimulus parameters and neurochemical lesions. Journal of Neural Transmission 88, 61–75. https://doi.org/10.1007/BF01245037

Ehlers, C.L., Chaplin, R.I., 1991. EEG and ERP response to chronic ethanol exposure in rats. Psychopharmacology 104, 67–74. https://doi.org/10.1007/BF02244556

Ehlers, C. L., Chaplin, R.I., Lumeng, L., Li, T.K., 1991. Electrophysiological Response to Ethanol in P and NP Rats. Alcoholism: Clinical and Experimental Research 15, 739–744. https://doi.org/10.1111/j.1530-0277.1991.tb00590.x

Ehlers, C.L., Kaneko, W.M., Robledo, P., Lopez, A.L., 1994. Long-latency event-related potentials in rats: Effects of task and stimulus parameters. Neuroscience 62, 759–769. https://doi.org/10.1016/0306-4522(94)90474-X

Ehlers, C.L., Wall, T.L., Chaplin, R.I., 1991. Long latency event-related potentials in rats: Effects of dopaminergic and serotonergic depletions. Pharmacology Biochemistry and Behavior 38, 789–793. https://doi.org/10.1016/0091-3057(91)90243-U

Ehrlé, N., 2001. Processing of rapid auditory information in epileptic patients with left temporal lobe damage. Neuropsychologia 39, 525–531. https://doi.org/10.1016/S0028-3932(00)00121-4

Eidelberg, E., White, J.C., Brazier, M.A.B., 1959. The hippocampal arousal pattern in rabbits. Experimental Neurology 1, 483–490. https://doi.org/10.1016/0014-4886(59)90045-7

Elazar, Z., Adey, W.R., 1967. Spectral analysis of low frequency components in the electrical activity of the hippocampus during learning. Electroencephalography and Clinical Neurophysiology 23, 225–240. https://doi.org/10.1016/0013-4694(67)90119-8

Eldar, E., Ganor, O., Admon, R., Bleich, A., Hendler, T., 2007. Feeling the Real World: Limbic Response to Music Depends on Related Content. Cerebral Cortex 17, 2828–2840. https://doi.org/10.1093/cercor/bhm011

Enatsu, R., Gonzalez-Martinez, J., Bulacio, J., Kubota, Y., Mosher, J., Burgess, R.C., Najm, I., Nair, D.R., 2015. Connections of the limbic network: A corticocortical evoked potentials study. Cortex 62, 20–33. https://doi.org/10.1016/j.cortex.2014.06.018

Engel, A., Keller, P.E., 2011. The Perception of Musical Spontaneity in Improvised and Imitated Jazz Performances. Frontiers in Psychology 2. https://doi.org/10.3389/fpsyg.2011.00083

Eraslan, E., Akyazi, İ., Ergül-Ekiz, E., Matur, E., 2015. Noise Stress-Induced Changes in mRNA Levels of Corticotropin-Releasing Hormone Family Molecules and Glucocorticoid Receptors in the Rat Brain. Folia Biologica (Praha) 61, 66–73.

Esclassan, F., Coutureau, E., Di Scala, G., Marchand, A.R., 2009. Differential contribution of dorsal and ventral hippocampus to trace and delay fear conditioning. Hippocampus 19, 33–44. https://doi.org/10.1002/hipo.20473

Euler, C.V., Green, J.D., 1960. Excitation, Inhibition and Rhythmical Activity in Hippocampal Pyramidal Cells in Rabbit. Acta Physiologica Scandinavica 48, 110–125. https://doi.org/10.1111/j.1748-1716.1960.tb01851.x

Ewing, S.G., Grace, A.A., 2013. Deep brain stimulation of the ventral hippocampus restores deficits in processing of auditory evoked potentials in a rodent developmental disruption model of schizophrenia. Schizophrenia Research 143, 377–383. https://doi.org/10.1016/j.schres.2012.11.023

Fast, C.D., Flesher, M.M., Nocera, N.A., Fanselow, M.S., Blaisdell, A.P., 2016. Learning history and cholinergic modulation in the dorsal hippocampus are necessary for rats to infer the status of a hidden event: HIPPOCAMPUS AND EXPERIENCE MODULATE RAT INFERENCES. Hippocampus 26, 804–815. https://doi.org/10.1002/hipo.22564

Faucher, P., Huguet, C., Mons, N., Micheau, J., 2022. Acute pre-learning stress selectively impairs hippocampus-dependent fear memory consolidation: Behavioral and molecular evidence. Neurobiology of Learning and Memory 188, 107585. https://doi.org/10.1016/j.nlm.2022.107585

Feldman, S., Dafny, N., 1968. Acoustic responses in the hypothalamus. Electroencephalography and Clinical Neurophysiology 25, 150–159.

Fernandes, C., File, S.E., 1993. Beware the builders: Construction noise changes [14C]GABA release and uptake from amygdaloid and hippocampal slices in the rat. Neuropharmacology 32, 1333–1336. https://doi.org/10.1016/0028-3908(93)90028-2

Ferrarese, C., Mennini, T., Pecora, N., Gobbi, M., Appollonio, I., Bernasconi, P., Frigo, M., Regondi, C., Pierpaoli, C., Frattola, L., Garattini, S., 1991. Acute noise stress in rats increases the levels of diazepam binding inhibitor (DBI) in hippocampus and adrenal gland. Psychopharmacology 103, 339–342. https://doi.org/10.1007/BF02244287

Finke, C., Esfahani, N.E., Ploner, C.J., 2012. Preservation of musical memory in an amnesic professional cellist. Current Biology 22, R591–R592. https://doi.org/10.1016/j.cub.2012.05.041

Fiorenza, N.G., Rosa, J., Izquierdo, I., Myskiw, J.C., 2012. Modulation of the extinction of two different fear-motivated tasks in three distinct brain areas. Behavioural Brain Research 232, 210–216. https://doi.org/10.1016/j.bbr.2012.04.015

Fitting, S., Booze, R.M., Hasselrot, U., Mactutus, C.F., 2006a. Intrahippocampal injections of Tat: Effects on prepulse inhibition of the auditory startle response in adult male rats. Pharmacology Biochemistry and Behavior 84, 189–196. https://doi.org/10.1016/j.pbb.2006.04.014

Fitting, S., Booze, R.M., Mactutus, C.F., 2006b. Neonatal hippocampal Tat injections: developmental effects on prepulse inhibition (PPI) of the auditory startle response. International Journal of Developmental Neuroscience 24, 275–283. https://doi.org/10.1016/j.ijdevneu.2006.02.001

Flynn, J.P., Wasman, M., 1960. Learning and Cortically Evoked Movement during Propagated Hippocampal Afterdischarges. Science 131, 1607–1608. https://doi.org/10.1126/science.131.3413.1607

Ford, J.H., Addis, D.R., Giovanello, K.S., 2011. Differential neural activity during search of specific and general autobiographical memories elicited by musical cues. Neuropsychologia 49, 2514–2526. https://doi.org/10.1016/j.neuropsychologia.2011.04.032

Foster, T., Hampson, R., West, M., Deadwyler, S., 1988. Control of sensory activation of granule cells in the fascia dentata by extrinsic afferents: septal and entorhinal inputs. J. Neurosci. 8, 3869–3878. https://doi.org/10.1523/JNEUROSCI.08-10-03869.1988

Foster, T.C., Christian, E.P., Hampson, R.E., Campbell, K.A., Deadwyler, S.A., 1987. Sequential dependencies regulate sensory evoked responses of single units in the rat hippocampus. Brain Research 408, 86–96. https://doi.org/10.1016/0006-8993(87)90361-1

Fox, S.S., Liebeskind, J.C., O’Brien, J.H., Dingle, R.D.H., 1967. Mechanisms for Limbic Modification of Cerebellar and Cortical Afferent Information, in: Progress in Brain Research. Elsevier, pp. 254–280. https://doi.org/10.1016/S0079-6123(08)63104-0

Freedman, R., Adler, L.E., Myles-Worsley, M., Nagamoto, H.T., Miller, C., Kisley, M., McRae, K., Cawthra, E., Waldo, M., 1996. Inhibitory Gating of an Evoked Response to Repeated Auditory Stimuli in Schizophrenic and Normal Subjects: Human Recordings, Computer Simulation, and an Animal Model. Arch Gen Psychiatry 53, 1114. https://doi.org/10.1001/archpsyc.1996.01830120052009

Freeman, F., Kramarcy, N., Lee, J., 1973. Discrimination learning and stimulus generalization in rats with hippocampal lesions. Physiology & Behavior 11, 273–275. https://doi.org/10.1016/0031-9384(73)90362-4

Freeman, J.H., Cuppernell, C., Flannery, K., Gabriel, M., 1996. Limbic thalamic, cingulate cortical and hippocampal neuronal correlates of discriminative approach learning in rabbits. Behavioural Brain Research 80, 123–136. https://doi.org/10.1016/0166-4328(96)00027-7

Freeman Jr., J.H., Weible, A., Rossi, J., Gabriel, M., 1997. Lesions of the entorhinal cortex disrupt behavioral and neuronal responses to context change during extinction of discriminative avoidance behavior: Experimental Brain Research 115, 445–457. https://doi.org/10.1007/PL00005714

Frenzilli, G., Ryskalin, L., Ferrucci, M., Cantafora, E., Chelazzi, S., Giorgi, F.S., Lenzi, P., Scarcelli, V., Frati, A., Biagioni, F., Gambardella, S., Falleni, A., Fornai, F., 2017. Loud Noise Exposure Produces DNA, Neurotransmitter and Morphological Damage within Specific Brain Areas. Frontiers in Neuroanatomy 11. https://doi.org/10.3389/fnana.2017.00049

Frisk, V., Milner, B., 1990a. The relationship of working memory to the immediate recall of stories following unilateral temporal or frontal lobectomy. Neuropsychologia 28, 121–135. https://doi.org/10.1016/0028-3932(90)90095-6

Frisk, V., Milner, B., 1990b. The role of the left hippocampal region in the acquisition and retention of story content. Neuropsychologia 28, 349–359. https://doi.org/10.1016/0028-3932(90)90061-R

Fritz, J., Mishkin, M., Saunders, R.C., 2005. In search of an auditory engram. Proceedings of the National Academy of Sciences 102, 9359–9364. https://doi.org/10.1073/pnas.0503998102

Fujioka, T., Zendel, B.R., Ross, B., 2010. Endogenous Neuromagnetic Activity for Mental Hierarchy of Timing. Journal of Neuroscience 30, 3458–3466. https://doi.org/10.1523/JNEUROSCI.3086-09.2010

Gabrieli, J.D.E., Cohen, N.J., Corkin, S., 1988. The impaired learning of semantic knowledge following bilateral medial temporal-lobe resection. Brain and Cognition 7, 157–177. https://doi.org/10.1016/0278-2626(88)90027-9

Gadian, D.G., Aicardi, J., Watkins, K.E., Porter, D.A., Mishkin, M., Vargha-Khadem, F., 2000. Developmental amnesia associated with early hypoxic-ischaemic injury. Brain 123, 499–507. https://doi.org/10.1093/brain/123.3.499

Gagnepain, P., Fauvel, B., Desgranges, B., Gaubert, M., Viader, F., Eustache, F., Groussard, M., Platel, H., 2017. Musical Expertise Increases Top–Down Modulation Over Hippocampal Activation during Familiarity Decisions. Frontiers in Human Neuroscience 11. https://doi.org/10.3389/fnhum.2017.00472

Gagnepain, P., Henson, R., Chételat, G., Desgranges, B., Lebreton, K., Eustache, F., 2011. Is Neocortical–Hippocampal Connectivity a Better Predictor of Subsequent Recollection than Local Increases in Hippocampal Activity? New Insights on the Role of Priming. Journal of Cognitive Neuroscience 23, 391–403. https://doi.org/10.1162/jocn.2010.21454

Gai, Z., Su, D., Wang, Y., Li, W., Cui, B., Li, K., She, X., Wang, R., 2017. Effects of chronic noise on the corticotropin-releasing factor system in the rat hippocampus: relevance to Alzheimer’s disease-like tau hyperphosphorylation. Environmental Health and Preventive Medicine 22. https://doi.org/10.1186/s12199-017-0686-8

Gaisler-Salomon, I., Wang, Y., Chuhma, N., Zhang, H., Golumbic, Y.N., Mihali, A., Arancio, O., Sibille, E., Rayport, S., 2012. Synaptic underpinnings of altered hippocampal function in glutaminase-deficient mice during maturation. Hippocampus 22, 1027–1039. https://doi.org/10.1002/hipo.22014

Gao, J., Wu, Y., Zhu, Z., Yang, C., Cheng, P., Liu, L., Sui, J., 2010. Neuronal firing activity of hippocampal pyramidal cells during an auditory discrimination task in conscious guinea pigs. Behavioural Brain Research 212, 35–40. https://doi.org/10.1016/j.bbr.2010.03.031

Gao, J., Zhang, D., Wang, L., Wang, W., Fan, Y., Tang, M., Zhang, Xin, Lei, X., Wang, Y., Yang, J., Zhang, Xiaoling, 2020. Altered Effective Connectivity in Schizophrenic Patients With Auditory Verbal Hallucinations: A Resting-State fMRI Study With Granger Causality Analysis. Front. Psychiatry 11, 575. https://doi.org/10.3389/fpsyt.2020.00575

Gardiner, J.M., Brandt, K.R., Baddeley, A.D., Vargha-Khadem, F., Mishkin, M., 2008. Charting the acquisition of semantic knowledge in a case of developmental amnesia. Neuropsychologia 46, 2865–2868. https://doi.org/10.1016/j.neuropsychologia.2008.05.021

Gelbard-Sagiv, H., Mukamel, R., Harel, M., Malach, R., Fried, I., 2008. Internally Generated Reactivation of Single Neurons in Human Hippocampus During Free Recall. Science 322, 96–101. https://doi.org/10.1126/science.1164685

Gentle, M.J., Wood-Gush, D.G.M., Gordon, J., 1978. Behavioural effects of hyperstriatal ablation in Gallus domesticus. Behavioural Processes 3, 137–148. https://doi.org/10.1016/0376-6357(78)90040-2

Ghika-Schmid, F., Ghika, J., Vuilleumier, P., Assal, G., Vuadens, P., Scherer, K., Maeder, P., Uske, A., Bogousslavsky, J., 1997. Bihippocampal damage with emotional dysfunction: Impaired auditory recognition of fear. European Neurology 38, 276–283.

Gil-da-Costa, R., Braun, A., Lopes, M., Hauser, M.D., Carson, R.E., Herscovitch, P., Martin, A., 2004. Toward an evolutionary perspective on conceptual representation: Species-specific calls activate visual and affective processing systems in the macaque. Proceedings of the National Academy of Sciences 101, 17516–17521. https://doi.org/10.1073/pnas.0408077101

Gilmartin, M.R., McEchron, M.D., 2005. Single Neurons in the Dentate Gyrus and CA1 of the Hippocampus Exhibit Inverse Patterns of Encoding During Trace Fear Conditioning. Behavioral Neuroscience 119, 164–179. https://doi.org/10.1037/0735-7044.119.1.164

Givens, B., 1996. Stimulus-evoked resetting of the dentate theta rhythm: relation to working memory. Neuroreport 8, 159–163. https://doi.org/10.1097/00001756-199612200-00032

Goble, T.J., Møller, A.R., Thompson, L.T., 2009. Acute high-intensity sound exposure alters responses of place cells in hippocampus. Hearing Research 253, 52–59. https://doi.org/10.1016/j.heares.2009.03.002

Goldstein, L.H., Canavan, A.G.M., Polkey, C.E., 1988. Verbal and Abstract Designs Paired Associate Learning After Unilateral Temporal Lobectomy. Cortex 24, 41–52. https://doi.org/10.1016/S0010-9452(88)80016-9

Gonzalez-Perez, O., Chavez-Casillas, O., Jauregui-Huerta, F., Lopez-Virgen, V., Guzman-Muniz, J., Moy-Lopez, N., Gonzalez-Castaneda, R.E., Luquin, S., 2011. Stress by noise produces differential effects on the proliferation rate of radial astrocytes and survival of neuroblasts in the adult subgranular zone. Neuroscience Research 70, 243–250. https://doi.org/10.1016/j.neures.2011.03.013

Good, M., de Hoz, L., Morris, R.G.M., 1998. Contingent versus incidental context processing during conditioning: Dissociation after excitotoxic hippocampal plus dentate gyrus lesions. Hippocampus 8, 147–159. https://doi.org/10.1002/(SICI)1098-1063(1998)8:2<147::AID-HIPO7>3.0.CO;2-I

Gosselin, N., Peretz, I., Hasboun, D., Baulac, M., Samson, S., 2011. Impaired recognition of musical emotions and facial expressions following anteromedial temporal lobe excision. Cortex 47, 1116–1125. https://doi.org/10.1016/j.cortex.2011.05.012

Gosselin, N., Peretz, I., Noulhiane, M., Hasboun, D., Beckett, C., Baulac, M., Samson, S., 2005. Impaired recognition of scary music following unilateral temporal lobe excision. Brain 128, 628–640. https://doi.org/10.1093/brain/awh420

Gottlieb, L.J., Uncapher, M.R., Rugg, M.D., 2010. Dissociation of the neural correlates of visual and auditory contextual encoding. Neuropsychologia 48, 137–144. https://doi.org/10.1016/j.neuropsychologia.2009.08.019

Gottlieb, L.J., Wong, J., de Chastelaine, M., Rugg, M.D., 2012. Neural correlates of the encoding of multimodal contextual features. Learning & Memory 19, 605–614. https://doi.org/10.1101/lm.027631.112

Grasby, P.M., Frith, C.D., Friston, K., Frackowiak, R.S.J., Dolan, R.J., 1993. Activation of the human hippocampal formation during auditory-verbal long-term memory function. Neuroscience Letters 163, 185–188. https://doi.org/10.1016/0304-3940(93)90378-X

Grastyán, E., Lissák, K., Madarász, I., Donhoffer, H., 1959. Hippocampal electrical activity during the development of conditioned reflexes. Electroencephalography and Clinical Neurophysiology 11, 409–430. https://doi.org/10.1016/0013-4694(59)90040-9

Grastyán, E., Vereczkei, L., 1974. Effects of spatial separation of the conditioned signal from the reinforcement: a demonstration of the conditioned character of the orienting response or the orientational character of conditioning. Behavioral Biology 10, 121–146. https://doi.org/10.1016/S0091-6773(74)91725-8

Graves, L., Dalvi, A., Lucki, I., Blendy, J.A., Abel, T., 2002. Behavioral analysis of CREB αΔ mutation on a B6/129 F1 hybrid background. Hippocampus 12, 18–26. https://doi.org/10.1002/hipo.10003

Green, J.D., Adey, W.R., 1956. Electrophysiological studies of hippocampal connections and excitability. Electroencephalography and Clinical Neurophysiology 8, 245–262. https://doi.org/10.1016/0013-4694(56)90117-1

Green, J.D., Arduini, A.A., 1954. Hippocampal electrical activity in arousal. Journal of Neurophysiology 17, 533–557. https://doi.org/10.1152/jn.1954.17.6.533

Green, J.D., Machne, X., 1955. Unit Activity of Rabbit Hippocampus. American Journal of Physiology-Legacy Content 181, 219–224. https://doi.org/10.1152/ajplegacy.1955.181.2.219

Grewe, P., Neu, D., Aengenendt, J., Woermann, F.G., Mertens, M., Bien, C.G., Kissler, J., 2020. Rhinal and hippocampal contributions to spontaneous inter-item binding and verbal memory recall: Evidence from temporal lobe epilepsy. Cortex 124, 204–216. https://doi.org/10.1016/j.cortex.2019.11.015

Groussard, M., La Joie, R., Rauchs, G., Landeau, B., Chételat, G., Viader, F., Desgranges, B., Eustache, F., Platel, H., 2010. When Music and Long-Term Memory Interact: Effects of Musical Expertise on Functional and Structural Plasticity in the Hippocampus. PLoS ONE 5, e13225. https://doi.org/10.1371/journal.pone.0013225

Groves, P.M., Wilson, C.J., Boyle, R.D., 1974. Brain Stem Pathways, Cortical Modulation, and Habituation of the Acoustic Startle Response I. Behavioral Biology 10, 391–418.

Grunwald, T., Boutros, N.N., Pezer, N., von Oertzen, J., Fernández, G., Schaller, C., Elger, C.E., 2003. Neuronal substrates of sensory gating within the human brain. Biological Psychiatry 53, 511–519. https://doi.org/10.1016/S0006-3223(02)01673-6

Gunbey, H.P., Gunbey, E., Aslan, K., Bulut, T., Unal, A., Incesu, L., 2015. Limbic-Auditory Interactions of Tinnitus: An Evaluation Using Diffusion Tensor Imaging. Clinical Neuroradiology 27, 221–230. https://doi.org/10.1007/s00062-015-0473-0

Gurevicius, K., Gureviciene, I., Valjakka, A., Schachner, M., Tanila, H., 2004. Enhanced cortical and hippocampal neuronal excitability in mice deficient in the extracellular matrix glycoprotein tenascin-R. Molecular and Cellular Neuroscience 25, 515–523. https://doi.org/10.1016/j.mcn.2003.12.001

Gurevicius, K., Kuang, F., Stoenica, L., Irintchev, A., Gureviciene, I., Dityatev, A., Schachner, M., Tanila, H., 2009. Genetic ablation of tenascin-C expression leads to abnormal hippocampal CA1 structure and electrical activity in vivo. Hippocampus 19, 1232–1246. https://doi.org/10.1002/hipo.20585

Gurevicius, K., Lipponen, A., Minkeviciene, R., Tanila, H., 2013. Short- and long-term habituation of auditory event-related potentials in the rat. F1000Research 2.

Gustafson, J.W., Lidsky, T.I., Schwartzbaum, J.S., 1975. Effects of hippocampal stimulation on acquisition, extinction, and generalization of conditioned suppression in the rat. Journal of Comparative and Physiological Psychology 89, 1136–1148. https://doi.org/10.1037/h0077179

Habib, R., McIntosh, A.R., Wheeler, M.A., Tulving, E., 2003. Memory encoding and hippocampally-based novelty/familiarity discrimination networks. Neuropsychologia 41, 271–279. https://doi.org/10.1016/S0028-3932(02)00160-4

Haider, S., Naqvi, F., Batool, Z., Tabassum, S., Perveen, T., Saleem, S., Haleem, D.H., 2012. Decreased Hippocampal 5-HT and DA Levels Following Sub-Chronic Exposure to Noise Stress: Impairment in both Spatial and Recognition Memory in Male Rats. Scientia Pharmaceutica 80, 1001–1011. https://doi.org/10.3797/scipharm.1207-15

Halgren, E., Baudena, P., Clarke, J.M., Heit, G., Marinkovic, K., Devaux, B., Vignal, J.-P., Biraben, A., 1995. Intracerebral potentials to rare target and distractor auditory and visual stimuli. II. Medial, lateral and posterior temporal lobe. Electroencephalography and Clinical Neurophysiology 94, 229–250. https://doi.org/10.1016/0013-4694(95)98475-N

Halgren, E., Squires, N., Wilson, C., Rohrbaugh, J., Babb, T., Crandall, P., 1980. Endogenous potentials generated in the human hippocampal formation and amygdala by infrequent events. Science 210, 803–805. https://doi.org/10.1126/science.7434000

Hall, R.D., Borbely, A.A., 1970. Acoustically evoked potentials in the rat during sleep and waking. Experimental Brain Research 11. https://doi.org/10.1007/BF00234203

Han, J.-S., Gallagher, M., Holland, P., 1998. Hippocampal lesions enhance configural learning by reducing proactive interference. Hippocampus 8, 138–146. https://doi.org/10.1002/(SICI)1098-1063(1998)8:2<138::AID-HIPO6>3.0.CO;2-H

Han, M.W., Ahn, J.H., Kang, J.K., Lee, E.M., Lee, J.H., Bae, J.H., Chung, J.W., 2011. Central auditory processing impairment in patients with temporal lobe epilepsy. Epilepsy & Behavior 20, 370–374. https://doi.org/10.1016/j.yebeh.2010.12.032

Hanna, A., Iremonger, K., Das, P., Dickson, D., Golde, T., Janus, C., 2012. Age-related increase in amyloid plaque burden is associated with impairment in conditioned fear memory in CRND8 mouse model of amyloidosis. Alzheimer’s Research & Therapy 4, 21. https://doi.org/10.1186/alzrt124

Hare, S.M., Adhikari, B.M., Du, X., Garcia, L., Bruce, H., Kochunov, P., Simon, J.Z., Hong, L.E., 2021. Local versus long-range connectivity patterns of auditory disturbance in schizophrenia. Schizophrenia Research 228, 262–270. https://doi.org/10.1016/j.schres.2020.11.052

Hattori, M., Onoda, K., Sakata, S., 2010. Identification of rat P3-like processes in the anterior cingulate cortex and hippocampus. Neuroscience Letters 472, 43–46. https://doi.org/10.1016/j.neulet.2010.01.052

Hawley, D.F., Leasure, J.L., 2011. Region-specific response of the hippocampus to chronic unpredictable stress. Hippocampus 22, 1338–1349. https://doi.org/10.1002/hipo.20970

Hayes, S.H., Manohar, S., Majumdar, A., Allman, B.L., Salvi, R., 2019. Noise-induced hearing loss alters hippocampal glucocorticoid receptor expression in rats. Hearing Research 379, 43–51. https://doi.org/10.1016/j.heares.2019.04.013

Heit, G., Smith, M.E., Halgren, E., 1990. Neuronal activity in the human medial temporal lobe during recognition memory. Brain 113, 1093–1112. https://doi.org/10.1093/brain/113.4.1093

Heldt, S.A., Coover, G.D., Falls, W.A., 2002. Posttraining but not pretraining lesions of the hippocampus interfere with feature-negative discrimination of fear-potentiated startle. Hippocampus 12, 774–786. https://doi.org/10.1002/hipo.10033

Helmstaedter, C., Elger, C.E., 1996. Cognitive Consequences of Two-Thirds Anterior Temporal Lobectomy on Verbal Memory in 144 Patients: A Three-Month Follow-Up Study. Epilepsia 37, 171–180. https://doi.org/10.1111/j.1528-1157.1996.tb00009.x

Helmstaedter, C., Grunwald, Th., Lehnertz, K., Gleißner, U., Elger, C.E., 1997. Differential Involvement of Left Temporolateral and Temporomesial Structures in Verbal Declarative Learning and Memory: Evidence from Temporal Lobe Epilepsy. Brain and Cognition 35, 110–131. https://doi.org/10.1006/brcg.1997.0930

Hendrickson, C.W., Kimble, R.J., Kimble, D.P., 1969. Hippocampal lesions and the orienting response. Journal of Comparative and Physiological Psychology 67, 220–227. https://doi.org/10.1037/h0026757

Henin, S., Turk-Browne, N.B., Friedman, D., Liu, A., Dugan, P., Flinker, A., Doyle, W., Devinsky, O., Melloni, L., 2021. Learning hierarchical sequence representations across human cortex and hippocampus. Sci. Adv. 7, eabc4530. https://doi.org/10.1126/sciadv.abc4530

Herdener, M., Esposito, F., di Salle, F., Boller, C., Hilti, C.C., Habermeyer, B., Scheffler, K., Wetzel, S., Seifritz, E., Cattapan-Ludewig, K., 2010. Musical Training Induces Functional Plasticity in Human Hippocampus. Journal of Neuroscience 30, 1377–1384. https://doi.org/10.1523/JNEUROSCI.4513-09.2010

Herholz, S.C., Coffey, E.B.J., Pantev, C., Zatorre, R.J., 2016. Dissociation of Neural Networks for Predisposition and for Training-Related Plasticity in Auditory-Motor Learning. Cerebral Cortex 26, 3125–3134. https://doi.org/10.1093/cercor/bhv138

Hershman, K.M., Freedman, R., Bickford, P.C., 1995. GABAB antagonists diminish the inhibitory gating of auditory response in the rat hippocampus. Neuroscience Letters 190, 133–136. https://doi.org/10.1016/0304-3940(95)11523-Y

Herz, A., 1965. Cortical and Subcortical Auditory Evoked Potentials during Wakefulness and Sleep in the Cat, in: Progress in Brain Research. Elsevier, pp. 63–69. https://doi.org/10.1016/S0079-6123(08)63583-9

Hilverman, C., Clough, S.A., Duff, M.C., Cook, S.W., 2018. Patients with hippocampal amnesia successfully integrate gesture and speech. Neuropsychologia 117, 332–338. https://doi.org/10.1016/j.neuropsychologia.2018.06.012

Hirano, T., 1984. Unit activity of the septo-hippocampal system in classical conditioning with rewarding brain stimulation. Brain Research 295, 41–49. https://doi.org/10.1016/0006-8993(84)90814-X

Hirano, T., Best, P., Olds, J., 1970. Units during habituation, discrimination learning, and extinction. Electroencephalography and Clinical Neurophysiology 28, 127–135. https://doi.org/10.1016/0013-4694(70)90180-X

Hirano, T., Yamaguchi, M., 1985. Hippocampal unit response during temporal single alternation of classical conditioning with rewarding brain stimulation in the rat. Physiological Psychology 13, 7–14. https://doi.org/10.3758/BF03326488

Hobin, J.A., Ji, J., Maren, S., 2006. Ventral hippocampal muscimol disrupts context-specific fear memory retrieval after extinction in rats. Hippocampus 16, 174–182. https://doi.org/10.1002/hipo.20144

Hoehler, F.K., Thompson, R.F., 1980. Effect of the interstimulus (CS–UCS) interval on hippocampal unit activity during classical conditioning of the nictitating membrane response of the rabbit (Oryctolagus cuniculus). Journal of Comparative and Physiological Psychology 94, 201–215. https://doi.org/10.1037/h0077658

Hoehler, F.K., Thompson, R.F., 1979. The effect of temporal single alternation on learned increases in hippocampal unit activity in classical conditioning of the rabbit nictitating membrane response. Physiological Psychology 7, 345–351. https://doi.org/10.3758/BF03326655

Holland, P.C., Lamoureux, J.A., Han, J.-S., Gallagher, M., 1999. Hippocampal lesions interfere with Pavlovian negative occasion setting. Hippocampus 9, 143–157. https://doi.org/10.1002/(SICI)1098-1063(1999)9:2<143::AID-HIPO6>3.0.CO;2-Z

Holt, W., Maren, S., 1999. Muscimol Inactivation of the Dorsal Hippocampus Impairs Contextual Retrieval of Fear Memory. J. Neurosci. 19, 9054–9062. https://doi.org/10.1523/JNEUROSCI.19-20-09054.1999

Honey, R.C., Good, M., 2000. Associative modulation of the orienting response: Distinct effects revealed by hippocampal lesions. Journal of Experimental Psychology: Animal Behavior Processes 26, 3–14.

Honey, R.C., Watt, A., Good, M., 1998. Hippocampal Lesions Disrupt an Associative Mismatch Process. The Journal of Neuroscience 18, 2226–2230. https://doi.org/10.1523/JNEUROSCI.18-06-02226.1998

Hosseini-Sharifabad, M., Sabahi, A., 2008. Exposure to chronic noise reduces the volume of hippocampal subregions in rats. Iranian Journal of Basic Medical Sciences 11, 18–24.

Hu, L., Yang, J., Song, T., Hou, N., Liu, Y., Zhao, X., Zhang, D., Wang, L., Wang, T., Huang, C., 2014. A new stress model, a scream sound, alters learning and monoamine levels in rat brain. Physiology & Behavior 123, 105–113. https://doi.org/10.1016/j.physbeh.2013.09.010

Huerta, P.T., Sun, L.D., Wilson, M.A., Tonegawa, S., 2000. Formation of Temporal Memory Requires NMDA Receptors within CA1 Pyramidal Neurons. Neuron 25, 473–480. https://doi.org/10.1016/S0896-6273(00)80909-5

Huet-Bello, O., Ruvalcaba-Delgadillo, Y., Feria-Velasco, A., Gonzalez-Castaneda, R.E., Garcia-Estrada, J., Macias-Islas, M.A., Jauregui-Huerta, F., Luquin, S., 2017. Environmental noise exposure modifies astrocyte morphology in hippocampus of young male rats. Noise and Health 19, 239–244.

Hunsaker, M.R., Kesner, R.P., 2008. Dissociations across the dorsal–ventral axis of CA3 and CA1 for encoding and retrieval of contextual and auditory-cued fear. Neurobiology of Learning and Memory 89, 61–69. https://doi.org/10.1016/j.nlm.2007.08.016

Insausti, R., Amaral, D.G., Cowan, W.M., 1987a. The entorhinal cortex of the monkey: II. Cortical afferents. The Journal of Comparative Neurology 264, 356–395. https://doi.org/10.1002/cne.902640306

Insausti, R., Amaral, D.G., Cowan, W.M., 1987b. The entorhinal cortex of the monkey: III. Subcortical afferents. The Journal of Comparative Neurology 264, 396–408. https://doi.org/10.1002/cne.902640307

Inta, D., Vogt, M.A., Elkin, H., Weber, T., Lima-Ojeda, J.M., Schneider, M., Luoni, A., Riva, M.A., Gertz, K., Hellmann-Regen, J., Kronenberg, G., Meyer-Lindenberg, A., Sprengel, R., Gass, P., 2014. Phenotype of mice with inducible ablation of GluA1 AMPA receptors during late adolescence: Relevance for mental disorders: BEHAVIORAL CHANGES FOLLOWING ADOLESCENT GLUA1 ABLATION. Hippocampus 24, 424–435. https://doi.org/10.1002/hipo.22236

Iordanova, M.D., Burnett, D.J., Aggleton, J.P., Good, M., Honey, R.C., 2009. The role of the hippocampus in mnemonic integration and retrieval: complementary evidence from lesion and inactivation studies. European Journal of Neuroscience 30, 2177–2189. https://doi.org/10.1111/j.1460-9568.2009.07010.x

Iordanova, Mihaela D., Burnett, D.J., Good, M., Honey, R.C., 2011. Pattern memory involves both elemental and configural processes: Evidence from the effects of hippocampal lesions. Behavioral Neuroscience 125, 567–577. https://doi.org/10.1037/a0023762

Iordanova, M. D., Good, M., Honey, R.C., 2011. Retrieval-Mediated Learning Involving Episodes Requires Synaptic Plasticity in the Hippocampus. Journal of Neuroscience 31, 7156–7162. https://doi.org/10.1523/JNEUROSCI.0295-11.2011

Irmiš, F., Radil-Weiss, T., Lát, J., Krekule, I., 1970. Inter-individual differences in hippocampal theta activity during habituation. Electroencephalography and Clinical Neurophysiology 28, 24–31. https://doi.org/10.1016/0013-4694(70)90004-0

Isaacson, R.L., Douglas, R.J., Moore, R.Y., 1961. The effect of radical hippocampal ablation on acquisition of avoidance response. Journal of Comparative and Physiological Psychology 54, 625–628. https://doi.org/10.1037/h0047286

Itskov, P.M., Vinnik, E., Honey, C., Schnupp, J., Diamond, M.E., 2012. Sound sensitivity of neurons in rat hippocampus during performance of a sound-guided task. Journal of Neurophysiology 107, 1822–1834. https://doi.org/10.1152/jn.00404.2011

Jablonowski, J., Taesler, P., Fu, Q., Rose, M., 2018. Implicit acoustic sequence learning recruits the hippocampus. PLOS ONE 13, e0209590. https://doi.org/10.1371/journal.pone.0209590

Jafari, Z., Okuma, M., Karem, H., Mehla, J., Kolb, B.E., Mohajerani, M.H., 2019. Prenatal noise stress aggravates cognitive decline and the onset and progression of beta amyloid pathology in a mouse model of Alzheimer’s disease. Neurobiology of Aging 77, 66–86. https://doi.org/10.1016/j.neurobiolaging.2019.01.019

Jafarpour, A., Piai, V., Lin, J.J., Knight, R.T., 2017. Human hippocampal pre-activation predicts behavior. Scientific Reports 7. https://doi.org/10.1038/s41598-017-06477-5

James, C.E., Britz, J., Vuilleumier, P., Hauert, C.-A., Michel, C.M., 2008. Early neuronal responses in right limbic structures mediate harmony incongruity processing in musical experts. NeuroImage 42, 1597–1608. https://doi.org/10.1016/j.neuroimage.2008.06.025

James, G.O., Hardiman, M.J., Yeo, C.H., 1987. Hippocampal lesions and trace conditioning in the rabbit. Behavioural Brain Research 23, 109–116. https://doi.org/10.1016/0166-4328(87)90048-9

Janata, P., 2002. The Cortical Topography of Tonal Structures Underlying Western Music. Science 298, 2167–2170. https://doi.org/10.1126/science.1076262

Jang, S.H., Choi, E.B., 2022. Evaluation of Structural Neural Connectivity Between the Primary Auditory Cortex and Cognition-Related Brain Areas Using Diffusion Tensor Tractography in 43 Normal Adults. Med Sci Monit 28. https://doi.org/10.12659/MSM.936131

Jarrard, L.E., Davidson, T.L., 1991. On the hippocampus and learned conditional responding: Effects of aspiration versus ibotenate lesions. Hippocampus 1, 107–117. https://doi.org/10.1002/hipo.450010110

Jáuregui-Huerta, F., García-Estrada, J., Ruvalcaba-Delgadillo, Y., Trujillo, X., Huerta, M., Feria-Velasco, A., Gonzalez-Perez, O., Luquín, S., 2011. Chronic exposure of juvenile rats to environmental noise impairs hippocampal cell proliferation in adulthood. Noise and Health 13, 286. https://doi.org/10.4103/1463-1741.82961

Jayakar, R., King, T.Z., Morris, R., Na, S., 2015. Hippocampal volume and auditory attention on a verbal memory task with adult survivors of pediatric brain tumor. Neuropsychology 29, 303–319. https://doi.org/10.1037/neu0000183

Jin, S.G., Kim, M.J., Park, S.Y., Park, S.N., 2017. Stress hormonal changes in the brain and plasma after acute noise exposure in mice. Auris Nasus Larynx 44, 272–276. https://doi.org/10.1016/j.anl.2016.07.013

Jirsa, R., Poc, R., Radil, T., 1992. Hippocampal Auditory Evoked Response Threshold in the Rat Behavioral Modulation. Brain Research Bulletin 28, 149–153.

Joassin, F., Pesenti, M., Maurage, P., Verreckt, E., Bruyer, R., Campanella, S., 2011. Cross-modal interactions between human faces and voices involved in person recognition. Cortex 47, 367–376. https://doi.org/10.1016/j.cortex.2010.03.003

Jones, E.G., Powell, T.P.S., 1970. An anatomical study of converging sensory pathways within the cerebral cortex of the monkey. Brain 93, 793–820. https://doi.org/10.1093/brain/93.4.793

Jung, R., Kornmüller, A.E., 1938. Eine Methodik der Ableitung Iokalisierter Potentialschwankungen aus subcorticalen Hirngebieten. Archiv für Psychiatrie und Nervenkrankheiten 109, 1–30. https://doi.org/10.1007/BF02157817

Kaifosh, P., Lovett-Barron, M., Turi, G.F., Reardon, T.R., Losonczy, A., 2013. Septo-hippocampal GABAergic signaling across multiple modalities in awake mice. Nature Neuroscience 16, 1182–1184. https://doi.org/10.1038/nn.3482

Kalm, K., Davis, M.H., Norris, D., 2013. Individual Sequence Representations in the Medial Temporal Lobe. Journal of Cognitive Neuroscience 25, 1111–1121. https://doi.org/10.1162/jocn_a_00378

Kaneko, W.M., Riley, E.P., Ehlers, C.L., 1993. Electrophysiological and behavioral findings in rats prenatally exposed to alcohol. Alcohol 10, 169–178. https://doi.org/10.1016/0741-8329(93)90099-A

Kapolowicz, M.R., Thompson, L.T., 2016. Acute high-intensity noise induces rapid Arc protein expression but fails to rapidly change GAD expression in amygdala and hippocampus of rats: Effects of treatment with D-cycloserine. Hearing Research 342, 69–79. https://doi.org/10.1016/j.heares.2016.09.010

Karashima, A., Nakamura, K., Horiuchi, M., Nakao, M., Katayama, N., Yamamoto, M., 2002. Elicited ponto-geniculo-occipital waves by auditory stimuli are synchronized with hippocampal theta-waves. Psychiatry and Clinical Neurosciences 56, 343–344. https://doi.org/10.1046/j.1440-1819.2002.01019.x

Karmonik, C., Brandt, A., Anderson, J.R., Brooks, F., Lytle, J., Silverman, E., Frazier, J.T., 2016. Music Listening Modulates Functional Connectivity and Information Flow in the Human Brain. Brain Connectivity 6, 632–641. https://doi.org/10.1089/brain.2016.0428

Karmos, G., Grastyán, E., 1962. Influence of hippocampal lesions on simple and delayed conditional reflexes. Acta Physiologica Academiae Scientiarum Hungaricae 21, 215–224.

Karmos, G., Grastyán, E., Losonczy, H., Vereczeky, L., Grosz, J., 1965. The possible role of the hippocampus in the organization of the orientation reaction. Acta Physiologica Academiae Scientiarum Hungaricae 26, 131.

Kathpalia, P., Nag, T.C., Chattopadhyay, P., Sharma, A., Bhat, M.A., Roy, T.S., Wadhwa, S., 2019. In ovo Sound Stimulation Mediated Regulation of BDNF in the Auditory Cortex and Hippocampus of Neonatal Chicks. Neuroscience 408, 293–307. https://doi.org/10.1016/j.neuroscience.2019.04.014

Kato, T., Erhard, P., Takayama, Y., Strupp, J., Le, T.H., Ogawa, S., Ugurbil, K., 1998. Human hippocampal long-term sustained response during word memory processing. NeuroReport 9, 1041–1047.

Kemble, E.D., Ison, J.R., 1971. Limbic lesions and the inhibition of startle reactions in the rat by conditions of preliminary stimulation. Physiology & Behavior 7, 925–928. https://doi.org/10.1016/0031-9384(71)90068-0

Kemp, I.R., Kaada, B.R., 1975. The relation of hippocampal theta activity to arousal, attentive behaviour and somato-motor movements in unrestrained cats. Brain Research 95, 323–342. https://doi.org/10.1016/0006-8993(75)90110-9

Kenney, J.W., Raybuck, J.D., Gould, T.J., 2012. Nicotinic receptors in the dorsal and ventral hippocampus differentially modulate contextual fear conditioning. Hippocampus 22, 1681–1690. https://doi.org/10.1002/hipo.22003

Khalfa, S., Guye, M., Peretz, I., Chapon, F., Girard, N., Chauvel, P., Liégeois-Chauvel, C., 2008. Evidence of lateralized anteromedial temporal structures involvement in musical emotion processing. Neuropsychologia 46, 2485–2493. https://doi.org/10.1016/j.neuropsychologia.2008.04.009

Kikuchi, Y., Endo, H., Yoshizawa, S., Kait, M., Nishimura, C., Tanaka, M., Kumagai, T., Takeda, T., 1997. Human cortico-hippocampal activity related to auditory discrimination revealed by neuromagnetic field. NeuroReport 8, 1657–1661.

Kim, B.-K., Ko, I.-G., Kim, S.-E., Kim, C.-J., Yoon, J.-S., Baik, H.-H., Jin, B.-K., Lee, C.-Y., Baek, S.-B., Shin, M.-S., 2013. Impact of Several Types of Stresses on Short-term Memory and Apoptosis in the Hippocampus of Rats. Int Neurourol J 17, 114–120. https://doi.org/10.5213/inj.2013.17.3.114

Kim, H., Lee, M.-H., Chang, H.-K., Lee, T.-H., Lee, H.-H., Shin, M.-C., Shin, M.-S., Won, R., Shin, H.-S., Kim, C.-J., 2006. Influence of prenatal noise and music on the spatial memory and neurogenesis in the hippocampus of developing rats. Brain and Development 28, 109–114. https://doi.org/10.1016/j.braindev.2005.05.008

Kim, J.J., Clark, R.E., Thompson, R.F., 1995. Hippocampectomy Impairs the Memory of Recently, but Not Remotely, Acquired Trace Eyeblink Conditioned Responses. Behavioral Neuroscience 109, 195–203.

Kim, T.-W., Shin, M.-S., Park, J.-K., Shin, M.-A., Lee, H.-H., Lee, S.-J., 2013. Treadmill exercise alleviates prenatal noise stress-induced impairment of spatial learning ability through enhancing hippocampal neurogenesis in rat pups. Journal of Exercise Rehabilitation 9, 451–456. https://doi.org/10.12965/jer.130064

Kimura, A., Donishi, T., Sakoda, T., Hazama, M., Tamai, Y., 2003. Auditory thalamic nuclei projections to the temporal cortex in the rat. Neuroscience 117, 1003–1016. https://doi.org/10.1016/S0306-4522(02)00949-1

Kirste, I., Nicola, Z., Kronenberg, G., Walker, T.L., Liu, R.C., Kempermann, G., 2015. Is silence golden? Effects of auditory stimuli and their absence on adult hippocampal neurogenesis. Brain Structure and Function 220, 1221–1228. https://doi.org/10.1007/s00429-013-0679-3

Klarner, A., Koch, M., Schnitzler, H.-U., 1998. Induction of Fos-protein in the forebrain and disruption of sensorimotor gating following N-methyl-d-aspartate infusion into the ventral hippocampus of the rat. Neuroscience 84, 443–452. https://doi.org/10.1016/S0306-4522(97)00475-2

Kleber, B., Birbaumer, N., Veit, R., Trevorrow, T., Lotze, M., 2007. Overt and imagined singing of an Italian aria. NeuroImage 36, 889–900. https://doi.org/10.1016/j.neuroimage.2007.02.053

Klee, J.L., Souza, B.C., Battaglia, F.P., 2021. Learning differentially shapes prefrontal and hippocampal activity during classical conditioning. eLife 10, e65456. https://doi.org/10.7554/eLife.65456

Klinkenberg, I., Sambeth, A., Blokland, A., 2013. Cholinergic gating of hippocampal auditory evoked potentials in freely moving rats. European Neuropsychopharmacology 23, 988–997. https://doi.org/10.1016/j.euroneuro.2012.08.014

Knösche, T.R., Neuhaus, C., Haueisen, J., Alter, K., Maess, B., Witte, O.W., Friederici, A.D., 2005. Perception of phrase structure in music: Perception of Phrase Structure in Music. Human Brain Mapping 24, 259–273. https://doi.org/10.1002/hbm.20088

Koelsch, S., Fritz, T., v. Cramon, D.Y., Müller, K., Friederici, A.D., 2006. Investigating emotion with music: An fMRI study. Human Brain Mapping 27, 239–250. https://doi.org/10.1002/hbm.20180

Koelsch, S., Remppis, A., Sammler, D., Jentschke, S., Mietchen, D., Fritz, T., Bonnemeier, H., Siebel, W.A., 2007. A cardiac signature of emotionality: A cardiac signature of emotionality. European Journal of Neuroscience 26, 3328–3338. https://doi.org/10.1111/j.1460-9568.2007.05889.x

Koelsch, S., Skouras, S., 2014. Functional centrality of amygdala, striatum and hypothalamus in a “small-world” network underlying joy: An fMRI study with music: A Neural Network Underlying Joy. Human Brain Mapping 35, 3485–3498. https://doi.org/10.1002/hbm.22416

Koelsch, S., Skouras, S., Fritz, T., Herrera, P., Bonhage, C., Küssner, M.B., Jacobs, A.M., 2013. The roles of superficial amygdala and auditory cortex in music-evoked fear and joy. NeuroImage 81, 49–60. https://doi.org/10.1016/j.neuroimage.2013.05.008

Köhler, C., 1976. Habituation after dorsal hippocampal lesions: a test dependent phenomenon. Behavioral Biology 18, 89–110. https://doi.org/10.1016/S0091-6773(76)91780-6

Kok, P., Rait, L.I., Turk-Browne, N.B., 2020. Content-based Dissociation of Hippocampal Involvement in Prediction. Journal of Cognitive Neuroscience 32, 527–545. https://doi.org/10.1162/jocn_a_01509

Kok, P., Turk-Browne, N.B., 2018. Associative Prediction of Visual Shape in the Hippocampus. The Journal of Neuroscience 38, 6888–6899. https://doi.org/10.1523/JNEUROSCI.0163-18.2018

Kotelenko, L.M., Fed’ko, L.I., Shustin, V.A., 2007. The subjective auditory space of epileptic patients with lesions in both the temporal cortical area and the hippocampus. Human Physiology 33, 539–545. https://doi.org/10.1134/S0362119707050040

Kotelenko, L.M., Nikitin, N.I., Altman, Ya.A., 2013. Estimation by humans of signals simulating different sound movement directions and specificity of the perception of these signals by patients with temporal epilepsy. Human Physiology 39, 241–247. https://doi.org/10.1134/S0362119713030122

Kotz, S.A., Kalberlah, C., Bahlmann, J., Friederici, A.D., Haynes, J.-D., 2013. Predicting vocal emotion expressions from the human brain. Human Brain Mapping 34, 1971–1981. https://doi.org/10.1002/hbm.22041

Kowalska, D.M., 1999. Effects of the anterior temporal lobe lesions, separate or combined with hippocampal damage, on spatial delayed responses guided by auditory stimulus. Acta Neurobiol Exp (Wars) 59, 303–313.

Kowalska, D.M., 1995. Effects of hippocampal lesions on spatial delayed responses in dog. Hippocampus 5, 363–370. https://doi.org/10.1002/hipo.450050409

Kramis, R., Vanderwolf, C.H., Bland, B.H., 1975. Two types of hippocampal rhythmical slow activity in both the rabbit and the rat: Relations to behavior and effects of atropine, diethyl ether, urethane, and pentobarbital. Experimental Neurology 49, 58–85. https://doi.org/10.1016/0014-4886(75)90195-8

Kraus, K.S., Mitra, S., Jimenez, Z., Hinduja, S., Ding, D., Jiang, H., Gray, L., Lobarinas, E., Sun, W., Salvi, R.J., 2010. Noise trauma impairs neurogenesis in the rat hippocampus. Neuroscience 167, 1216–1226. https://doi.org/10.1016/j.neuroscience.2010.02.071

Krause, M., Hoffmann, W.E., Hajós, M., 2003. Auditory sensory gating in hippocampus and reticular thalamic neurons in anesthetized rats. Biological Psychiatry 53, 244–253. https://doi.org/10.1016/S0006-3223(02)01463-4

Kreyberg, S., Torvik, A., Bjørneboe, A., Wilk-Larsen, W., Jacobsen, D., 1992. Trimethyltin poisoning: report of a case with postmortem examination. Clinical Neuropathology 11, 256–259.

Kropotov, J.D., Alho, K., Näätänen, R., Ponomarev, V.A., Kropotova, O.V., Anichkov, A.D., Nechaev, V.B., 2000. Human auditory-cortex mechanisms of preattentive sound discrimination. Neuroscience Letters 280, 87–90. https://doi.org/10.1016/S0304-3940(00)00765-5

Kubit, B., Janata, P., 2018. Listening for memories: Attentional focus dissociates functional brain networks engaged by memory-evoking music. Psychomusicology: Music, Mind, and Brain 28, 82–100. https://doi.org/10.1037/pmu0000210

Kumar, S., Bonnici, H.M., Teki, S., Agus, T.R., Pressnitzer, D., Maguire, E.A., Griffiths, T.D., 2014. Representations of specific acoustic patterns in the auditory cortex and hippocampus. Proceedings of the Royal Society B: Biological Sciences 281, 20141000–20141000. https://doi.org/10.1098/rspb.2014.1000

Kumar, S., Gander, P.E., Berger, J.I., Billig, A.J., Nourski, K.V., Oya, H., Kawasaki, H., Howard, M.A., Griffiths, T.D., 2021. Oscillatory correlates of auditory working memory examined with human electrocorticography. Neuropscyhologia 150. https://doi.org/10.1101/2020.06.19.161901

Kumar, S., Joseph, S., Gander, P.E., Barascud, N., Halpern, A.R., Griffiths, T.D., 2016. A Brain System for Auditory Working Memory. Journal of Neuroscience 36, 4492–4505. https://doi.org/10.1523/JNEUROSCI.4341-14.2016

Kurczek, J., Brown-Schmidt, S., Duff, M., 2013. Hippocampal contributions to language: Evidence of referential processing deficits in amnesia. Journal of Experimental Psychology: General 142, 1346–1354. https://doi.org/10.1037/a0034026

Kurioka, T., Mogi, S., Yamashita, T., 2021. Decreasing auditory input induces neurogenesis impairment in the hippocampus. Sci Rep 11, 423. https://doi.org/10.1038/s41598-020-80218-z

Lackner, J., 1974. Observations on the speech processing capabilities of an amnesic patient: Several aspects of H.M.’s language function. Neuropsychologia 12, 199–207. https://doi.org/10.1016/0028-3932(74)90005-0

Lahti, A.C., Weiler, M.A., Holcomb, H.H., Tamminga, C.A., Carpenter, W.T., McMahon, R., 2006. Correlations Between rCBF and Symptoms in Two Independent Cohorts of Drug-Free Patients with Schizophrenia. Neuropsychopharmacol 31, 221–230. https://doi.org/10.1038/sj.npp.1300837

Lai, H., 1988. Effects of repeated exposure to white noise on central cholinergic activity in the rat. Brain Research 442, 403–406. https://doi.org/10.1016/0006-8993(88)91535-1

Lai, H., 1987. Acute exposure to noise affects sodium-dependent high-affinity choline uptake in the central nervous system of the rat. Pharmacology Biochemistry and Behavior 28, 147–151. https://doi.org/10.1016/0091-3057(87)90205-X

Lai, H., Carino, M.A., 1990. Acute White Noise Exposure Affects the Concentration of Benzodiazepine Receptors in the Brain of the Rat. Pharmacology Biochemistry and Behavior 36, 985–987.

Lai, H., Carino, M.A., Wen, Y.-F., 1989. Repeated noise exposure affects muscarinic cholinergic receptors in the rat brain. Brain Research 488, 361–364. https://doi.org/10.1016/0006-8993(89)90731-2

Lan, L., Chen, Y.-C., Shang, S., Lu, L., Xu, J.-J., Yin, X., Wu, Y., Cai, Y., 2022. Topological features of limbic dysfunction in chronicity of tinnitus with intact hearing: New hypothesis for ‘noise-cancellation’ mechanism. Progress in Neuro-Psychopharmacology and Biological Psychiatry 113, 110459. https://doi.org/10.1016/j.pnpbp.2021.110459

Lancelot, C., Ahad, P., Noulhiane, M., Hasboun, D., Baulac, M., Samson, S., 2005. Loss of memory for auditory–spatial associations following unilateral medial temporal-lobe damage. Neuropsychologia 43, 1975–1982. https://doi.org/10.1016/j.neuropsychologia.2005.03.006

Lancelot, C., Ahad, P., Noulhiane, M., Hasboun, D., Baulac, M., Samson, S., 2003a. Spatial and non-spatial auditory short-term memory in patients with temporal-lobe lesion: NeuroReport 14, 2203–2207. https://doi.org/10.1097/00001756-200312020-00014

Lancelot, C., Samson, S., Ahad, P., Baulac, M., 2003b. Effect of unilateral temporal lobe resection on short-term memory for auditory object and sound location. Annals of the New York Academy of Sciences 999, 377–380.

Landgrebe, M., Langguth, B., Rosengarth, K., Braun, S., Koch, A., Kleinjung, T., May, A., de Ridder, D., Hajak, G., 2009. Structural brain changes in tinnitus: Grey matter decrease in auditory and non-auditory brain areas. NeuroImage 46, 213–218. https://doi.org/10.1016/j.neuroimage.2009.01.069

Langers, D.R.M., Melcher, J.R., 2011. Hearing Without Listening: Functional Connectivity Reveals the Engagement of Multiple Nonauditory Networks During Basic Sound Processing. Brain Connectivity 1, 233–244. https://doi.org/10.1089/brain.2011.0023

Laroche, S., Falcou, R., Bloch, V., 1983. Post-trial reticular facilitation of associative changes in multiunit activity: comparison between dentate gyrus and entorhinal cortex. Behavioural Brain Research 9, 381–387. https://doi.org/10.1016/0166-4328(83)90139-0

Laroche, S., Neuenschwander-el Massioui, N., Edeline, J.-M., Dutrieux, G., 1987. Hippocampal associative cellular responses: dissociation with behavioral responses revealed by a transfer-of-control technique. Behavioral and Neural Biology 47, 356–368. https://doi.org/10.1016/S0163-1047(87)90474-2

Laursen, B., Mørk, A., Kristiansen, U., Bastlund, J.F., 2014. Hippocampal P3-Like Auditory Event-Related Potentials are Disrupted in a Rat Model of Cholinergic Degeneration in Alzheimer’s Disease: Reversal by Donepezil Treatment. Journal of Alzheimer’s Disease 42, 1179–1189. https://doi.org/10.3233/JAD-131502

Leaton, R.N., 1981. Habituation of startle response, lick suppression, and exploratory behavior in rats with hippocampal lesions. Journal of Comparative and Physiological Psychology 95, 813–826. https://doi.org/10.1037/h0077832

Leaton, R.N., Buck, R.L., 1971. Habituation of the arousal response in rats. Journal of Comparative and Physiological Psychology 75, 430–434. https://doi.org/10.1037/h0030931

Lee, C.H., Kim, K.W., Lee, S.M., Kim, S.Y., 2020. Effect of acute noise trauma on the gene expression profile of the hippocampus. BMC Neurosci 21, 45. https://doi.org/10.1186/s12868-020-00599-9

Lee, I., Kesner, R.P., 2004. Differential contributions of dorsal hippocampal subregions to memory acquisition and retrieval in contextual fear-conditioning. Hippocampus 14, 301–310. https://doi.org/10.1002/hipo.10177

Lee, S.-H., Huh, N., Lee, J.W., Ghim, J.-W., Lee, I., Jung, M.W., 2017. Neural Signals Related to Outcome Evaluation Are Stronger in CA1 than CA3. Frontiers in Neural Circuits 11. https://doi.org/10.3389/fncir.2017.00040

Lee, S.-M., Kim, B.-K., Kim, T.-W., Ji, E.-S., Choi, H.-H., 2016. Music application alleviates short-term memory impairments through increasing cell proliferation in the hippocampus of valproic acid-induced autistic rat pups. Journal of Exercise Rehabilitation 12, 148–155. https://doi.org/10.12965/jer.1632638.319

Lee, V., MacKenzie, G., Hooper, A., Maguire, J., 2016. Reduced tonic inhibition in the dentate gyrus contributes to chronic stress-induced impairments in learning and memory: Stress Decreases Tonic Inhibition and Impairs Memory. Hippocampus 26, 1276–1290. https://doi.org/10.1002/hipo.22604

Lee, Y., Davis, M., 1997a. Role of the Hippocampus, the Bed Nucleus of the Stria Terminalis, and the Amygdala in the Excitatory Effect of Corticotropin-Releasing Hormone on the Acoustic Startle Reflex. The Journal of Neuroscience 17, 6434–6446. https://doi.org/10.1523/JNEUROSCI.17-16-06434.1997

Lee, Y., Davis, M., 1997b. Role of the Septum in the Excitatory Effect of Corticotropin-Releasing Hormone on the Acoustic Startle Reflex. The Journal of Neuroscience 17, 6424–6433. https://doi.org/10.1523/JNEUROSCI.17-16-06424.1997

Lehne, M., Rohrmeier, M., Koelsch, S., 2014. Tension-related activity in the orbitofrontal cortex and amygdala: an fMRI study with music. Social Cognitive and Affective Neuroscience 9, 1515–1523. https://doi.org/10.1093/scan/nst141

Lennox, B.R., Park, S.B.G., Medley, I., Morris, P.G., Jones, P.B., 2000. The functional anatomy of auditory hallucinations in schizophrenia. Psychiatry Research: Neuroimaging 100, 13–20. https://doi.org/10.1016/S0925-4927(00)00068-8

Lerner, Y., Papo, D., Zhdanov, A., Belozersky, L., Hendler, T., 2009. Eyes Wide Shut: Amygdala Mediates Eyes-Closed Effect on Emotional Experience with Music. PLoS ONE 4, e6230. https://doi.org/10.1371/journal.pone.0006230

Li, B., Cui, L.-B., Xi, Y.-B., Friston, K.J., Guo, F., Wang, H.-N., Zhang, L.-C., Bai, Y.-H., Tan, Q.-R., Yin, H., Lu, H., 2017. Abnormal Effective Connectivity in the Brain is Involved in Auditory Verbal Hallucinations in Schizophrenia. Neurosci. Bull. 33, 281–291. https://doi.org/10.1007/s12264-017-0101-x

Li, K., Jia, H., She, X., Cui, B., Zhang, N., Chen, X., Xu, C., An, G., Ma, Q., 2014. Role of NMDA receptors in noise-induced tau hyperphosphorylation in rat hippocampus and prefrontal cortex. Journal of the Neurological Sciences 340, 191–197. https://doi.org/10.1016/j.jns.2014.03.027

Liang, J.C., Wagner, A.D., Preston, A.R., 2013. Content Representation in the Human Medial Temporal Lobe. Cerebral Cortex 23, 80–96. https://doi.org/10.1093/cercor/bhr379

Liberman, T., Velluti, R.A., Pedemonte, M., 2009. Temporal correlation between auditory neurons and the hippocampal theta rhythm induced by novel stimulations in awake guinea pigs. Brain Research 1298, 70–77. https://doi.org/10.1016/j.brainres.2009.08.061

Liberson, W.T., Cadilhac, J.G., 1953. Electroshock and Rhinencephalic Seizure States. Stereotactic and Functional Neurosurgery 13, 278–286. https://doi.org/10.1159/000105425

Liddle, P.F., Lane, C.J., Ngan, E.T.C., 2000. Immediate effects of risperidone on cortico–striato–thalamic loops and the hippocampus. British Journal of Psychiatry 177, 402–407. https://doi.org/10.1192/bjp.177.5.402

Lidsky, T. I., Levine, M.S., MacGregor, S., 1974. Tonic and phasic effects evoked concurrently by sensory stimuli in hippocampal units. Experimental Neurology 44, 130–134.

Lidsky, T.I., Levine, M.S., MacGregor, S., 1974. Hippocampal units during orienting and arousal in rabbits. Experimental Neurology 44, 171–186. https://doi.org/10.1016/0014-4886(74)90057-0

Lin, T.-C.E., Dumigan, N.M., Good, M., Honey, R.C., 2016. Novel sensory preconditioning procedures identify a specific role for the hippocampus in pattern completion. Neurobiology of Learning and Memory 130, 142–148. https://doi.org/10.1016/j.nlm.2016.02.006

Lin, T.-C.E., Honey, R.C., 2011. Encoding specific associative memory: Evidence from behavioral and neural manipulations. Journal of Experimental Psychology: Animal Behavior Processes 37, 317–329. https://doi.org/10.1037/a0022497

Lin, X., Zhuo, C., Li, G., Li, J., Gao, X., Chen, C., Jiang, D., 2020. Functional brain alterations in auditory hallucination subtypes in individuals with auditory hallucinations without the diagnosis of specific neurological diseases and mental disorders at the current stage. Brain Behav 10. https://doi.org/10.1002/brb3.1487

Linke, R., 1999. Organization of projections to temporal cortex originating in the thalamic posterior intralaminar nucleus of the rat. Experimental Brain Research 127, 314–320. https://doi.org/10.1007/s002210050801

Lipska, B.K., Weinberger, D.R., Swerdlow, N.R., Geyer, M.A., Braff, D.L., Jaskiw, G.E., 1995. Neonatal excitotoxic hippocampal damage in rats causes post-pubertal changes in prepulse inhibition of startle and its disruption by apomorphine. Psychopharmacology 122, 35–43. https://doi.org/10.1007/BF02246439

Liu, J., Lin, T., Yan, X., Jiang, W., Shi, M., Ye, R., Rao, Z., Zhao, G., 2010. Effects of infrasound on cell proliferation in the dentate gyrus of adult rats: NeuroReport 21, 585–589. https://doi.org/10.1097/WNR.0b013e32833a7dc4

Liu, L., Shen, P., He, T., Chang, Y., Shi, L., Tao, S., Li, X., Xun, Q., Guo, X., Yu, Z., Wang, J., 2016. Noise induced hearing loss impairs spatial learning/memory and hippocampal neurogenesis in mice. Scientific Reports 6. https://doi.org/10.1038/srep20374

Liu, L., Xuan, C., Shen, P., He, T., Chang, Y., Shi, L., Tao, S., Yu, Z., Brown, R.E., Wang, J., 2018. Hippocampal Mechanisms Underlying Impairment in Spatial Learning Long After Establishment of Noise-Induced Hearing Loss in CBA Mice. Frontiers in Systems Neuroscience 12. https://doi.org/10.3389/fnsys.2018.00035

Livanov, M.N., Dudaeva, K.I., Vinogradova, O.S., 1971. Effect of reticular formation on hippocampal neurons (field CA1). Neirofiziologiya 3, 227–235.

Lockwood, A.H., Salvi, R.J., Coad, M.L., Towsley, M.L., Wack, D.S., Murphy, B.W., 1998. The functional neuroanatomy of tinnitus. Neurology 50, 114–120.

Loechner, K.J., Weisz, D.J., 1987. Hippocampectomy and feature-positive discrimination. Behavioural Brain Research 26, 63–73. https://doi.org/10.1016/0166-4328(87)90017-9

Long, L.L., Hinman, J.R., Chen, C.-M.A., Stevenson, I.H., Read, H.L., Escabi, M.A., Chrobak, J.J., 2014. Novel acoustic stimuli can alter locomotor speed to hippocampal theta relationship: Sound and Hippocampal Theta. Hippocampus 24, 1053–1058. https://doi.org/10.1002/hipo.22308

Lositsky, O., Chen, J., Toker, D., Honey, C.J., Shvartsman, M., Poppenk, J.L., Hasson, U., Norman, K.A., 2016. Neural pattern change during encoding of a narrative predicts retrospective duration estimates. eLife 5, 40.

LoTurco, J.L., Coulter, D.A., Alkon, D.L., 1988. Enhancement of synaptic potentials in rabbit CA1 pyramidal neurons following classical conditioning. PNAS 85, 1672–1676. https://doi.org/10.1073/pnas.85.5.1672

Love, S., Pollick, F.E., Latinus, M., 2011. Cerebral Correlates and Statistical Criteria of Cross-Modal Face and Voice Integration. Seeing and Perceiving 24, 351–367. https://doi.org/10.1163/187847511X584452

Lovett-Barron, M., Kaifosh, P., Kheirbek, M.A., Danielson, N., Zaremba, J.D., Reardon, T.R., Turi, G.F., Hen, R., Zemelman, B.V., Losonczy, A., 2014. Dendritic Inhibition in the Hippocampus Supports Fear Learning. Science 343, 857–863. https://doi.org/10.1126/science.1247485

Lucas, E., Powell, E., Murphree, O., 1974. Hippocampal theta in nervous pointer dogs. Physiology & Behavior 12, 609–613. https://doi.org/10.1016/0031-9384(74)90210-8

Luntz-Leybman, V., Bickford, P.C., Freedman, R., 1992. Cholinergic gating of response to auditory stimuli in rat hippocampus. Brain Research 587, 130–136. https://doi.org/10.1016/0006-8993(92)91437-J

Luria, A.R., Karasseva, T.A., 1968. Disturbances of auditory-speech memory in focal lesions of the deep regions of the left temporal lobe. Neuropsychologia 6, 97-IN1. https://doi.org/10.1016/0028-3932(68)90051-1

Ma, J., Leung, L.S., 2011. GABAB receptor blockade in the hippocampus affects sensory and sensorimotor gating in Long-Evans rats. Psychopharmacology 217, 167–176. https://doi.org/10.1007/s00213-011-2274-8

Ma, J., Tai, S.K., Leung, L.S., 2009. Ketamine-induced deficit of auditory gating in the hippocampus of rats is alleviated by medial septal inactivation and antipsychotic drugs. Psychopharmacology 206, 457–467. https://doi.org/10.1007/s00213-009-1623-3

MacKay, D.G., Stewart, R., Burke, D.M., 1998. H.M. Revisited: Relations between Language Comprehension, Memory, and the Hippocampal System. Journal of Cognitive Neuroscience 10, 377–394. https://doi.org/10.1162/089892998562807

MacLean, Paul D, C., Flanigan, S., Flynn, J.P., Kim, C., Stevens, J.R., 1955. Hippocampal function: Tentative correlations of conditioning, EEG, drug, and radioautographic studies. Yale Journal of Biology and Medicine 28, 16.

Madroñal, N., Delgado-García, J.M., Fernández-Guizán, A., Chatterjee, J., Köhn, M., Mattucci, C., Jain, A., Tsetsenis, T., Illarionova, A., Grinevich, V., Gross, C.T., Gruart, A., 2016. Rapid erasure of hippocampal memory following inhibition of dentate gyrus granule cells. Nature Communications 7. https://doi.org/10.1038/ncomms10923

Maguire, E.A., Frith, C.D., 2004. The brain network associated with acquiring semantic knowledge. NeuroImage 22, 171–178. https://doi.org/10.1016/j.neuroimage.2003.12.036

Maguire, E.A., Mummery, C.J., 1999. Differential modulation of a common memory retrieval network revealed by positron emission tomography. Hippocampus 9, 54–61. https://doi.org/10.1002/(SICI)1098-1063(1999)9:1<54::AID-HIPO6>3.0.CO;2-O

Maller, J.J., Welton, T., Middione, M., Callaghan, F.M., Rosenfeld, J.V., Grieve, S.M., 2019. Revealing the Hippocampal Connectome through Super-Resolution 1150-Direction Diffusion MRI. Scientific Reports 9. https://doi.org/10.1038/s41598-018-37905-9

Manahan-Vaughan, D., von Haebler, D., Winter, C., Juckel, G., Heinemann, U., 2008. A single application of MK801 causes symptoms of acute psychosis, deficits in spatial memory, and impairment of synaptic plasticity in rats. Hippocampus 18, 125–134. https://doi.org/10.1002/hipo.20367

Manan, H.A., Franz, E.A., Yusoff, A.N., Sarah Mukari, S.Z.-M., 2012. Hippocampal-cerebellar involvement in enhancement of performance in word-based BRT with the presence of background noise: An initial fMRI study. Psychology & Neuroscience 5, 247–256. https://doi.org/10.3922/j.psns.2012.2.16

Manikandan, S., Padma, M.K., Srikumar, R., Jeya Parthasarathy, N., Muthuvel, A., Devi, R.S., 2006. Effects of chronic noise stress on spatial memory of rats in relation to neuronal dendritic alteration and free radical-imbalance in hippocampus and medial prefrontal cortex. Neuroscience Letters 399, 17–22. https://doi.org/10.1016/j.neulet.2006.01.037

Mao, X., Cai, D., Lou, W., 2022. Music alleviates pain perception in depression mouse models by promoting the release of glutamate in the hippocampus of mice to act on GRIK5. Nucleosides, Nucleotides & Nucleic Acids 1–11. https://doi.org/10.1080/15257770.2022.2051048

Maren, S., 1999. Neurotoxic or electrolytic lesions of the ventral subiculum produce deficits in the acquisition and expression of Pavlovian fear conditioning in rats. Behav Neurosci 113, 283–290. https://doi.org/10.1037//0735-7044.113.2.283

Maren, S., Holt, W.G., 2004. Hippocampus and Pavlovian Fear Conditioning in Rats: Muscimol Infusions Into the Ventral, but Not Dorsal, Hippocampus Impair the Acquisition of Conditional Freezing to an Auditory Conditional Stimulus. Behavioral Neuroscience 118, 97–110. https://doi.org/10.1037/0735-7044.118.1.97

Martin, S.M., Moberg, G.P., Horowitz, J.M., 1975. Glucocorticoids and the hippocampal theta rhythm in loosely restrained, unanesthetized rabbits. Brain Research 93, 535–542. https://doi.org/10.1016/0006-8993(75)90195-X

Martorell, A.J., Paulson, A.L., Suk, H.-J., Abdurrob, F., Drummond, G.T., Guan, W., Young, J.Z., Kim, D.N.-W., Kritskiy, O., Barker, S.J., Mangena, V., Prince, S.M., Brown, E.N., Chung, K., Boyden, E.S., Singer, A.C., Tsai, L.-H., 2019. Multi-sensory Gamma Stimulation Ameliorates Alzheimer’s-Associated Pathology and Improves Cognition. Cell 177, 256-271.e22. https://doi.org/10.1016/j.cell.2019.02.014

Mascagni, F., McDonald, A.J., Coleman, J.R., 1993. Corticoamygdaloid and corticocortical projections of the rat temporal cortex: APhaseolus vulgaris leucoagglutinin study. Neuroscience 57, 697–715. https://doi.org/10.1016/0306-4522(93)90016-9

Massioui, N., Dutrieux, G., Edeline, J.-M., 1991. Conditioned Hippocampal Cellular Response to a Behaviorally Silent Conditioned Stimulus. Behavioral Neuroscience 105, 313–325.

Mastroianni, P.P., 1979. Hippocampal lesions and the generalization of auditory stimuli. Neuropsychologia 17, 401–412. https://doi.org/10.1016/0028-3932(79)90086-1

Matt, L., Eckert, P., Panford-Walsh, R., Geisler, H.-S., Bausch, A.E., Manthey, M., Müller, N.I.C., Harasztosi, C., Rohbock, K., Ruth, P., Friauf, E., Ott, T., Zimmermann, U., Rüttiger, L., Schimmang, T., Knipper, M., Singer, W., 2018. Visualizing BDNF Transcript Usage During Sound-Induced Memory Linked Plasticity. Frontiers in Molecular Neuroscience 11. https://doi.org/10.3389/fnmol.2018.00260

Mayer, A., Hanlon, F., Franco, A., Teshiba, T., Thoma, R., Clark, V., Canive, J., 2009. The neural networks underlying auditory sensory gating. NeuroImage 44, 182–189. https://doi.org/10.1016/j.neuroimage.2008.08.025

Mayes, A.R., Holdstock, J.S., Isaac, C.L., Montaldi, D., Grigor, J., Gummer, A., Cariga, P., Downes, J.J., Tsivilis, D., Gaffan, D., Gong, Q., Norman, K.A., 2004. Associative recognition in a patient with selective hippocampal lesions and relatively normal item recognition. Hippocampus 14, 763–784. https://doi.org/10.1002/hipo.10211

Mays, L.E., Best, P.J., 1975. Hippocampal unit activity to tonal stimuli during arousal from sleep and in awake rats. Experimental Neurology 47, 268–279. https://doi.org/10.1016/0014-4886(75)90256-3

McCarthy, G., Wood, C., Williamson, P., Spencer, D., 1989. Task-dependent field potentials in human hippocampal formation. The Journal of Neuroscience 9, 4253–4268. https://doi.org/10.1523/JNEUROSCI.09-12-04253.1989

McDonald, R.J., Murphy, R.A., Guarraci, F.A., Gortler, J.R., White, N.M., Baker, A.G., 1997. Systematic comparison of the effects of hippocampal and fornix-fimbria lesions on acquisition of three configural discriminations. Hippocampus 7, 371–388. https://doi.org/10.1002/(SICI)1098-1063(1997)7:4<371::AID-HIPO3>3.0.CO;2-M

McEchron, M.D., Bouwmeester, H., Tseng, W., Weiss, C., Disterhoft, J.F., 1998. Hippocampectomy disrupts auditory trace fear conditioning and contextual fear conditioning in the rat. Hippocampus 8, 638–646. https://doi.org/10.1002/(SICI)1098-1063(1998)8:6<638::AID-HIPO6>3.0.CO;2-Q

McEchron, M.D., Disterhoft, J.F., 1997. Sequence of Single Neuron Changes in CA1 Hippocampus of Rabbits During Acquisition of Trace Eyeblink Conditioned Responses. Journal of Neurophysiology 78, 1030–1044. https://doi.org/10.1152/jn.1997.78.2.1030

McEchron, M.D., Tseng, W., Disterhoft, J.F., 2003. Single Neurons in CA1 Hippocampus Encode Trace Interval Duration during Trace Heart Rate (Fear) Conditioning in Rabbit. The Journal of Neuroscience 23, 1535–1547. https://doi.org/10.1523/JNEUROSCI.23-04-01535.2003

McEchron, M.D., Tseng, W., Disterhoft, J.F., 2000. Neurotoxic lesions of the dorsal hippocampus disrupt auditory-cued trace heart rate (fear) conditioning in rabbits. Hippocampus 10, 739–751. https://doi.org/10.1002/1098-1063(2000)10:6<739::AID-HIPO1011>3.0.CO;2-I

McEchron, M.D., Weible, A.P., Disterhoft, J.F., 2001. Aging and Learning-Specific Changes in Single-Neuron Activity in CA1 Hippocampus During Rabbit Trace Eyeblink Conditioning. Journal of Neurophysiology 86, 1839–1857. https://doi.org/10.1152/jn.2001.86.4.1839

McGlinchey-Berroth, R., Carrillo, M.C., 1997. Impaired Trace Eyeblink Conditioning in Bilateral, Medial-Temporal Lobe Amnesia. Behavioural Neuroscience 111, 873–882.

McHugh, S.B., Marques-Smith, A., Li, J., Rawlins, J.N.P., Lowry, J., Conway, M., Gilmour, G., Tricklebank, M., Bannerman, D.M., 2013. Hemodynamic responses in amygdala and hippocampus distinguish between aversive and neutral cues during Pavlovian fear conditioning in behaving rats. European Journal of Neuroscience 37, 498–507. https://doi.org/10.1111/ejn.12057

McHugh, T.J., Tonegawa, S., 2009. CA3 NMDA receptors are required for the rapid formation of a salient contextual representation. Hippocampus 19, 1153–1158. https://doi.org/10.1002/hipo.20684

McMillan, T.M., Powell, G.E., Janota, I., Polkey, C.E., 1987. Relationships between neuropathology and cognitive functioning in temporal lobectomy patients. Journal of Neurology, Neurosurgery & Psychiatry 50, 167–176. https://doi.org/10.1136/jnnp.50.2.167

McOmish, C.E., Burrows, E.L., Howard, M., Hannan, A.J., 2008. PLC-β1 knockout mice as a model of disrupted cortical development and plasticity: Behavioral endophenotypes and dysregulation of RGS4 gene expression. Hippocampus 18, 824–834. https://doi.org/10.1002/hipo.20443

Meador, K.J., Loring, D.W., King, D.W., Gallagher, B.B., Gould, M.J., Flanigin, H.F., Smith, J.R., 1987. Limbic evoked potentials predict site of epileptic focus. Neurology 37, 494–494. https://doi.org/10.1212/WNL.37.3.494

Meador, K.J., Loring, D.W., King, D.W., Gallagher, B.B., Gould, M.J., Smith, J.R., Flanigin, H.F., 1988. Cholinergic Modulation of Human Limbic Evoked Potentials. International Journal of Neuroscience 38, 407–414. https://doi.org/10.3109/00207458808990701

Meck, W.H., 1988. Hippocampal Function Is Required for Feedback Control of an Internal Clock’s Criterion. Behavioral Neuroscience 102, 54–60.

Meck, W.H., Church, R.M., Olton, D.S., 1984. Hippocampus, time, and memory. Behavioral Neuroscience 98, 3–22.

Meck, W.H., Church, R.M., Wenk, G.L., Olton, D.S., 1987. Nucleus basalis magnocellularis and medial septal area lesions differentially impair temporal memory. The Journal of Neuroscience 7, 3505–3511. https://doi.org/10.1523/JNEUROSCI.07-11-03505.1987

Melgire, M., Ragot, R., Samson, S., Penney, T.B., Meck, W.H., Pouthas, V., 2005. Auditory/visual duration bisection in patients with left or right medial-temporal lobe resection. Brain and Cognition 58, 119–124. https://doi.org/10.1016/j.bandc.2004.09.013

Meng, B., Zhu, S., Li, S., Zeng, Q., Mei, B., 2009. Global view of the mechanisms of improved learning and memory capability in mice with music-exposure by microarray. Brain Research Bulletin 80, 36–44. https://doi.org/10.1016/j.brainresbull.2009.05.020

Merhav, M., Karni, A., Gilboa, A., 2014. Neocortical catastrophic interference in healthy and amnesic adults: A paradoxical matter of time: Catastrophic Interference in Human Declarative Memory. Hippocampus 24, 1653–1662. https://doi.org/10.1002/hipo.22353

Meyer, P., Mecklinger, A., Grunwald, T., Fell, J., Elger, C.E., Friederici, A.D., 2005. Language processing within the human medial temporal lobe. Hippocampus 15, 451–459. https://doi.org/10.1002/hipo.20070

Meyer, V., Yates, A.J., 1955. Intellectual changes following temporal lobectomy for psychomotor epilepsy: Preliminary communication. Journal of Neurology, Neurosurgery & Psychiatry 18, 44–52. https://doi.org/10.1136/jnnp.18.1.44

Micco, D.J., Schwartz, M., 1971. Effects of hippocampal lesions upon the development of Pavlovian internal inhibition in rats. Journal of Comparative and Physiological Psychology 76, 371–377. https://doi.org/10.1037/h0031376

Michelmann, S., Price, A.R., Aubrey, B., Strauss, C.K., Doyle, W.K., Friedman, D., Dugan, P.C., Devinsky, O., Devore, S., Flinker, A., Hasson, U., Norman, K.A., 2021. Moment-by-moment tracking of naturalistic learning and its underlying hippocampo-cortical interactions. Nat Commun 12, 5394. https://doi.org/10.1038/s41467-021-25376-y

Mickley, G.A., Ferguson, J.L., 1989. Enhanced acoustic startle responding in rats with radiation-induced hippocampal granule cell hypoplasia. Experimental Brain Research 75. https://doi.org/10.1007/BF00248526

Miller, C., Bickford, P., Wiser, A., Rose, G., 1995. Long-term potentiation disrupts auditory gating in the rat hippocampus. J. Neurosci. 15, 5820–5830. https://doi.org/10.1523/JNEUROSCI.15-08-05820.1995

Miller, C.L., Bickford, P.C., Luntz-Leybman, V., Adler, L.E., Gerhardt, G.A., Freedman, R., 1992. Phencyclidine and auditory sensory gating in the hippocampus of the rat. Neuropharmacology 31, 1041–1048. https://doi.org/10.1016/0028-3908(92)90106-Y

Miller, C.L., Burmeister, M., Stevens, K.E., 1999. Hippocampal auditory gating in the hyperactive mocha mouse. Neuroscience Letters 276, 57–60. https://doi.org/10.1016/S0304-3940(99)00784-3

Miller, C.L., Freedman, R., 1995. The activity of hippocampal interneurons and pyramidal cells during the response of the hippocampus to repeated auditory stimuli. Neuroscience 69, 371–381. https://doi.org/10.1016/0306-4522(95)00249-I

Miller, C.L., Freedman, R., 1993. Medial septal neuron activity in relation to an auditory sensory gating paradigm. Neuroscience 55, 373–380. https://doi.org/10.1016/0306-4522(93)90506-B

Miller, S.W., Groves, P.M., 1977. Sensory evoked neuronal activity in the hippocampus before and after lesions of the medial septal nuclei. Physiology & Behavior 18, 141–146. https://doi.org/10.1016/0031-9384(77)90106-8

Milner, B., 1970. Memory and the medial temporal regions of the brain, in: Pribram, K., Broadbent, D. (Eds.), Biology of Memory. Academic Press, New York, NY, pp. 29–50.

Milner, B., 1958. Psychological defects produced by temporal lobe excision. Research Publications of the Association for Research in Nervous & Mental Disease 36, 244–257.

Milner, B., Teuber, H.L., 1968. Alteration of perception and memory in man: Reflections on methods, in: Weiskrantz, L. (Ed.), Analysis of Behavioral Change. Harper & Row, New York.

Miniaci, M.C., Lippiello, P., Monda, M., Scotto, P., 2016. Role of hippocampus in polymodal-cue guided tasks in rats. Brain Research 1646, 426–432. https://doi.org/10.1016/j.brainres.2016.06.030

Misane, I., Tovote, P., Meyer, M., Spiess, J., Ögren, S.O., Stiedl, O., 2005. Time-dependent involvement of the dorsal hippocampus in trace fear conditioning in mice. Hippocampus 15, 418–426. https://doi.org/10.1002/hipo.20067

Mitchell, R.L.C., Elliott, R., Barry, M., Cruttenden, A., Woodruff, P.W.R., 2003. The neural response to emotional prosody, as revealed by functional magnetic resonance imaging. Neuropsychologia 41, 1410–1421. https://doi.org/10.1016/S0028-3932(03)00017-4

Mitterschiffthaler, M.T., Fu, C.H.Y., Dalton, J.A., Andrew, C.M., Williams, S.C.R., 2007. A functional MRI study of happy and sad affective states induced by classical music. Human Brain Mapping 28, 1150–1162. https://doi.org/10.1002/hbm.20337

Modi, M.N., Dhawale, A.K., Bhalla, U.S., 2014. CA1 cell activity sequences emerge after reorganization of network correlation structure during associative learning. eLife 3. https://doi.org/10.7554/eLife.01982

Mohan, A., Davidson, C., De Ridder, D., Vanneste, S., 2018. Effective connectivity analysis of inter- and intramodular hubs in phantom sound perception – identifying the core distress network. Brain Imaging and Behavior. https://doi.org/10.1007/s11682-018-9989-7

Moita, M.A.P., Rosis, S., Zhou, Y., LeDoux, J.E., Blair, H.T., 2004. Putting Fear in Its Place: Remapping of Hippocampal Place Cells during Fear Conditioning. Journal of Neuroscience 24, 7015–7023. https://doi.org/10.1523/JNEUROSCI.5492-03.2004

Moita, M.A.P., Rosis, S., Zhou, Y., LeDoux, J.E., Blair, H.T., 2003. Hippocampal Place Cells Acquire Location-Specific Responses to the Conditioned Stimulus during Auditory Fear Conditioning. Neuron 37, 485–497. https://doi.org/10.1016/S0896-6273(03)00033-3

Mount, R.A., Sridhar, S., Hansen, K.R., Mohammed, A.I., Abdulkerim, M., Kessel, R., Nazer, B., Gritton, H.J., Han, X., 2021. Distinct neuronal populations contribute to trace conditioning and extinction learning in the hippocampal CA1. eLife 10, e56491. https://doi.org/10.7554/eLife.56491

Moxon, K.A., Gerhardt, G.A., Bickford, P.C., Austin, K., Rose, G.M., Woodward, D.J., Adler, L.E., 1999. Multiple single units and population responses during inhibitory gating of hippocampal auditory response in freely-moving rats. Brain Research 825, 75–85. https://doi.org/10.1016/S0006-8993(99)01187-7

Moyer, J.R., Deyo, R.A., Disterhoft, J.F., 1990. Hippocampectomy Disrupts Trace Eye-Blink Conditioning in Rabbits. Behavioral Neuroscience 104, 243–252.

Moyer, J.R.Jr., Thompson, L.T., Disterhoft, J.F., 1996. Trace Eyeblink Conditioning Increases CA1 Excitability in a Transient and Learning-Specific Manner. The Journal of Neuroscience 16, 5536–5546. https://doi.org/10.1523/JNEUROSCI.16-17-05536.1996

Mueller, K., Fritz, T., Mildner, T., Richter, M., Schulze, K., Lepsien, J., Schroeter, M.L., Möller, H.E., 2015. Investigating the dynamics of the brain response to music: A central role of the ventral striatum/nucleus accumbens. NeuroImage 116, 68–79. https://doi.org/10.1016/j.neuroimage.2015.05.006

Mueller, K., Mildner, T., Fritz, T., Lepsien, J., Schwarzbauer, C., Schroeter, M.L., Möller, H.E., 2011. Investigating brain response to music: A comparison of different fMRI acquisition schemes. NeuroImage 54, 337–343. https://doi.org/10.1016/j.neuroimage.2010.08.029

Mueller, S.G., Laxer, K.D., Scanlon, C., Garcia, P., McMullen, W.J., Loring, D.W., Meador, K.J., Weiner, M.W., 2012. Different structural correlates for verbal memory impairment in temporal lobe epilepsy with and without mesial temporal lobe sclerosis. Human Brain Mapping 33, 489–499. https://doi.org/10.1002/hbm.21226

Müller, N., Keil, J., Obleser, J., Schulz, H., Grunwald, T., Bernays, R.-L., Huppertz, H.-J., Weisz, N., 2013. You can’t stop the music: Reduced auditory alpha power and coupling between auditory and memory regions facilitate the illusory perception of music during noise. NeuroImage 79, 383–393. https://doi.org/10.1016/j.neuroimage.2013.05.001

Múnera, A., Gruart, A., Muñoz, M.D., Fernández-Mas, R., Delgado-García, J.M., 2001. Hippocampal Pyramidal Cell Activity Encodes Conditioned Stimulus Predictive Value During Classical Conditioning in Alert Cats. Journal of Neurophysiology 86, 2571–2582. https://doi.org/10.1152/jn.2001.86.5.2571

Muñoz, M., Insausti, R., 2005. Cortical efferents of the entorhinal cortex and the adjacent parahippocampal region in the monkey (Macaca fascicularis). European Journal of Neuroscience 22, 1368–1388. https://doi.org/10.1111/j.1460-9568.2005.04299.x

Musalek, M., Podreka, I., Walter, H., Suess, E., Passweg, V., Nutzinger, D., Strobl, R., Lesch, O.M., 1989. Regional brain function in hallucinations: A study of regional cerebral blood flow with 99m-Tc-HMPAO-SPECT in patients with auditory hallucinations, tactile hallucinations, and normal controls. Comprehensive Psychiatry 30, 99–108. https://doi.org/10.1016/0010-440X(89)90123-5

Musser, E.D., Kaiser-Laurent, H., Ablow, J.C., 2012. The neural correlates of maternal sensitivity: An fMRI study. Developmental Cognitive Neuroscience 2, 428–436. https://doi.org/10.1016/j.dcn.2012.04.003

Mutschler, I., Wieckhorst, B., Speck, O., Schulze-Bonhage, A., Hennig, J., Seifritz, E., Ball, T., 2010. Time Scales of Auditory Habituation in the Amygdala and Cerebral Cortex. Cerebral Cortex 20, 2531–2539. https://doi.org/10.1093/cercor/bhq001

Niki, H., 1967. Effects of Hippocampal Ablation on Learning in the Rat, in: Progress in Brain Research. Elsevier, pp. 305–317. https://doi.org/10.1016/S0079-6123(08)63107-6

Nishitani, N., Nagamine, akashi, Fujiwara, N., Yazawa, S., Shibasaki, H., 1998. Cortical-hippocampal auditory processing identified by magnetoencephalography. Journal of Cognitive Neuroscience 10, 231–247.

Nokia, M.S., Waselius, T., Mikkonen, J.E., Wikgren, J., Penttonen, M., 2015. Phase matters: responding to and learning about peripheral stimuli depends on hippocampal θ phase at stimulus onset. Learning & Memory 22, 307–317. https://doi.org/10.1101/lm.038166.115

Nokia, M.S., Wikgren, J., 2009. Hippocampal theta activity is selectively associated with contingency detection but not discrimination in rabbit discrimination-reversal eyeblink conditioning. Hippocampus NA-NA. https://doi.org/10.1002/hipo.20696

Oades, R.D., 1980. Discriminatory approach to auditory stimuli in Guinea fowl (Numida meleagris) after hyperstriatal/hippocampal brain damage. Behavioural Processes 5, 227–249. https://doi.org/10.1016/0376-6357(80)90005-4

O’Brien, C.E., Bowden, S.C., Bardenhagen, F.J., Cook, M.J., 2003. Neuropsychological correlates of hippocampal and rhinal cortex volumes in patients with mesial temporal sclerosis. Hippocampus 13, 892–904. https://doi.org/10.1002/hipo.10128

O’Connor, J.J., Rowan, M.J., Anwyl, R., 1992. Serotoninergic depression of auditory evoked responses recorded in the rat hippocampus: effect of repeated buspirone treatment. Brain Research 573, 190–196. https://doi.org/10.1016/0006-8993(92)90762-X

O’Connor, T.A., Starr, A., 1985. Intracranial potentials correlated with an event-related potential, P300, in the cat. Brain Research 339, 27–38. https://doi.org/10.1016/0006-8993(85)90618-3

Oehrn, C.R., Baumann, C., Fell, J., Lee, H., Kessler, H., Habel, U., Hanslmayr, S., Axmacher, N., 2015. Human Hippocampal Dynamics during Response Conflict. Current Biology 25, 2307–2313. https://doi.org/10.1016/j.cub.2015.07.032

Oh, J.-P., Han, J.-H., 2020. A critical role of hippocampus for formation of remote cued fear memory. Mol Brain 13, 112. https://doi.org/10.1186/s13041-020-00652-y

Okamoto, M., Katayama, T., Suzuki, Y., Hoshino, K.-Y., Yamada, H., Matsuoka, N., Jodo, E., 2012. Neonatal administration of phencyclidine decreases the number of putative inhibitory interneurons and increases neural excitability to auditory paired clicks in the hippocampal CA3 region of freely moving adult mice. Neuroscience 224, 268–281. https://doi.org/10.1016/j.neuroscience.2012.08.013

Olds, J., Disterhoft, J.F., Segal, M., Kornblith, C.L., Hirsh, R., 1972. Learning centers of rat brain mapped by measuring latencies of conditioned unit responses. Journal of Neurophysiology 35, 202–219. https://doi.org/10.1152/jn.1972.35.2.202

Olds, J., Hirano, T., 1969. Conditioned responses of hippocampal and other neurons. Electroencephalography and Clinical Neurophysiology 26, 159–166. https://doi.org/10.1016/0013-4694(69)90206-5

Oler, J.A., Penley, S.C., Sava, S., Markus, E.J., 2008. Does the dorsal hippocampus process navigational routes or behavioral context? A single-unit analysis. European Journal of Neuroscience 28, 802–812. https://doi.org/10.1111/j.1460-9568.2008.06375.x

Olton, D.S., Wenk, G.L., Church, R.M., Meck, W.H., 1988. Attention and the frontal cortex as examined by simultaneous temporal processing. Neuropsychologia 26, 307–318. https://doi.org/10.1016/0028-3932(88)90083-8

Omar, R., Henley, S.M.D., Bartlett, J.W., Hailstone, J.C., Gordon, E., Sauter, D.A., Frost, C., Scott, S.K., Warren, J.D., 2011. The structural neuroanatomy of music emotion recognition: Evidence from frontotemporal lobar degeneration. NeuroImage 56, 1814–1821. https://doi.org/10.1016/j.neuroimage.2011.03.002

Ong, J.L., Patanaik, A., Chee, N.I.Y.N., Lee, X.K., Poh, J.-H., Chee, M.W.L., 2018. Auditory stimulation of sleep slow oscillations modulates subsequent memory encoding through altered hippocampal function. Sleep 41. https://doi.org/10.1093/sleep/zsy031

Onoda, K., Takahashi, E., Sakata, S., 2003. Event-related potentials in the frontal cortex, hippocampus, and cerebellum during a temporal discrimination task in rats. Cognitive Brain Research 17, 380–387. https://doi.org/10.1016/S0926-6410(03)00139-3

Paller, K.A., McCarthy, G., Roessler, E., Allison, T., Wood, C.C., 1992. Potentials evoked in human and monkey medial temporal lobe during auditory and visual oddball paradigms. Electroencephalography and Clinical Neurophysiology/Evoked Potentials Section 84, 269–279. https://doi.org/10.1016/0168-5597(92)90008-Y

Paller, K.A., Ranganath, C., Gonsalves, B., LaBar, K.S., Parrish, T.B., Gitelman, D.R., Mesulam, M.-M., Reber, P.J., 2003. Neural Correlates of Person Recognition. Learning & Memory 10, 253–260. https://doi.org/10.1101/lm.57403

Pang, M.-H., Kim, N.-S., Kim, I.-H., Kim, H., Kim, H.-T., Choi, J.-S., 2010. Cholinergic transmission in the dorsal hippocampus modulates trace but not delay fear conditioning. Neurobiology of Learning and Memory 94, 206–213. https://doi.org/10.1016/j.nlm.2010.05.008

Papp, G., Kovac, S., Frese, A., Evers, S., 2014. The impact of temporal lobe epilepsy on musical ability. Seizure 23, 533–536. https://doi.org/10.1016/j.seizure.2014.03.018

Park, C., Falls, W., Finger, J.H., Longo-Guess, C.M., Ackerman, S.L., 2002. Deletion in Catna2, encoding αN-catenin, causes cerebellar and hippocampal lamination defects and impaired startle modulation. Nat Genet 31, 279–284. https://doi.org/10.1038/ng908

Park, H., Rugg, M.D., 2009. Prestimulus hippocampal activity predicts later recollection. Hippocampus NA-NA. https://doi.org/10.1002/hipo.20663

Parmeggiani, P.L., Lenzi, P., Azzaroni, A., D’Alessandro, R., 1982. Hippocampal influence on unit responses elicited in the cat’s auditory cortex by acoustic stimulation. Experimental Neurology 78, 259–274. https://doi.org/10.1016/0014-4886(82)90045-0

Parmeggiani, P.L., Rapisarda, C., 1969. Hippocampal output and sensory mechanisms. Brain Research 14, 387–400. https://doi.org/10.1016/0006-8993(69)90117-6

Patterson, M.M., Berger, T.W., Thompson, R.F., 1979. Neuronal plasticity recorded from cat hippocampus during classical conditioning. Brain Research 163, 339–343. https://doi.org/10.1016/0006-8993(79)90363-9

Paz, R., Gelbard-Sagiv, H., Mukamel, R., Harel, M., Malach, R., Fried, I., 2010. A neural substrate in the human hippocampus for linking successive events. Proceedings of the National Academy of Sciences 107, 6046–6051. https://doi.org/10.1073/pnas.0910834107

Pedemonte, M., Pérez-Perera, L., Peña, J.L., Velluti, R.A., 2001. Sleep and Wakefulness Auditory Processing: Cortical Units vs. Hippocampal Theta Rhythm. Sleep Research Online 4, 51–57.

Penfield, W., Milner, B., 1958. Memory Deficit Produced by Bilateral Lesions in the Hippocampal Zone 23.

Pereira, C.S., Teixeira, J., Figueiredo, P., Xavier, J., Castro, S.L., Brattico, E., 2011. Music and Emotions in the Brain: Familiarity Matters. PLoS ONE 6, e27241. https://doi.org/10.1371/journal.pone.0027241

Pereira, I., Agster, K.L., Burwell, R.D., 2016. Subcortical connections of the perirhinal, postrhinal, and entorhinal cortices of the rat. I. afferents. Hippocampus 26, 1189–1212. https://doi.org/10.1002/hipo.22603

Persson, J., Kalpouzos, G., Nilsson, L.-G., Ryberg, M., Nyberg, L., 2011. Preserved hippocampus activation in normal aging as revealed by fMRI. Hippocampus 21, 753–766. https://doi.org/10.1002/hipo.20794

Peters, J., Suchan, B., Köster, O., Daum, I., 2007. Domain-specific retrieval of source information in the medial temporal lobe: Domain-specific retrieval in the MTL. European Journal of Neuroscience 26, 1333–1343. https://doi.org/10.1111/j.1460-9568.2007.05752.x

Petersson, K.M., Gisselgård, J., Gretzer, M., Ingvar, M., 2006. Interaction between a verbal working memory network and the medial temporal lobe. NeuroImage 33, 1207–1217. https://doi.org/10.1016/j.neuroimage.2006.07.042

Petersson, K.M., Reis, A., Castro-Caldas, A., Ingvar, M., 1999. Effective Auditory–Verbal Encoding Activates the Left Prefrontal and the Medial Temporal Lobes: A Generalization to Illiterate Subjects. NeuroImage 10, 45–54. https://doi.org/10.1006/nimg.1999.0446

Phillips, M.L., Young, A.W., Scott, S.K., Calder, A.J., Andrew, C., Giampietro, V., Williams, S.C.R., Bullmore, E.T., Brammer, M., Gray, J.A., 1998. Neural responses to facial and vocal expressions of fear and disgust. Proceedings of the Royal Society of London. Series B: Biological Sciences 265, 1809–1817. https://doi.org/10.1098/rspb.1998.0506

Piai, V., Anderson, K.L., Lin, J.J., Dewar, C., Parvizi, J., Dronkers, N.F., Knight, R.T., 2016. Direct brain recordings reveal hippocampal rhythm underpinnings of language processing. Proc Natl Acad Sci USA 113, 11366–11371. https://doi.org/10.1073/pnas.1603312113

Pickenhain, L., Klingberg, F., 1967. Hippocampal Slow Wave Activity as a Correlate of Basic Behavioral Mechanisms in the Rat, in: Progress in Brain Research. Elsevier, pp. 218–227. https://doi.org/10.1016/S0079-6123(08)63101-5

Pisansky, M.T., Wickham, R.J., Su, J., Fretham, S., Yuan, L.-L., Sun, M., Gewirtz, J.C., Georgieff, M.K., 2013. Iron deficiency with or without anemia impairs prepulse inhibition of the startle reflex: Iron Deficiency Impairs Prepulse Inhibition. Hippocampus 23, 952–962. https://doi.org/10.1002/hipo.22151

Plailly, J., Tillmann, B., Royet, J.-P., 2007. The Feeling of Familiarity of Music and Odors: The Same Neural Signature? Cerebral Cortex 17, 2650–2658. https://doi.org/10.1093/cercor/bhl173

Pollmann, S., Lepsien, J., Hugdahl, K., Von Cramon, 2004. Auditory Target Detection in Dichotic Listening Involves the Orbitofrontal and Hippocampal Paralimbic Belts. Cerebral Cortex 14, 903–913. https://doi.org/10.1093/cercor/bhh049

Poppenk, J., Walia, G., McIntosh, A.R., Joanisse, M.F., Klein, D., Köhler, S., 2008. Why is the meaning of a sentence better remembered than its form? An fMRI study on the role of novelty-encoding processes. Hippocampus 18, 909–918. https://doi.org/10.1002/hipo.20453

Port, R.L., Beggs, A.L., Patterson, M.M., 1987. Hippocampal Substrate of Sensory Associations. Physiology & Behavior 39, 643–647.

Port, R.L., Patterson, M.M., 1984. Fimbrial Lesions and Sensory Preconditioning. Behavioral Neuroscience 98, 584–589.

Port, R.L., Romano, A.G., Steinmetz, J.E., Mikhail, A.A., Patterson, M.M., 1986. Retention and Acquisition of Classical Trace Conditioned Responses by Rabbits With Hippocampal Lesions. Behavioral Neuroscience 100, 745–752.

Poulin, B., Butcher, A., McWilliams, P., Bourgognon, J.-M., Pawlak, R., Kong, K.C., Bottrill, A., Mistry, S., Wess, J., Rosethorne, E.M., Charlton, S.J., Tobin, A.B., 2010. The M3-muscarinic receptor regulates learning and memory in a receptor phosphorylation/arrestin-dependent manner. Proceedings of the National Academy of Sciences 107, 9440–9445. https://doi.org/10.1073/pnas.0914801107

Prabhakar, J., Johnson, E.G., Nordahl, C.W., Ghetti, S., 2018. Memory-related hippocampal activation in the sleeping toddler. Proceedings of the National Academy of Sciences 115, 6500–6505. https://doi.org/10.1073/pnas.1805572115

Puce, A., Kalnins, R.M., Berkovic, S.F., Bladin, P.F., 1989. Limbic P3 potentials, seizure localization, and surgical pathology in temporal lobe epilepsy. Annals of Neurology 26, 377–385. https://doi.org/10.1002/ana.410260311

Quinn, J.J., Loya, F., Ma, Q.D., Fanselow, M.S., 2005. Dorsal hippocampus NMDA receptors differentially mediate trace and contextual fear conditioning. Hippocampus 15, 665–674. https://doi.org/10.1002/hipo.20088

Quinn, J.J., Oommen, S.S., Morrison, G.E., Fanselow, M.S., 2002. Post-training excitotoxic lesions of the dorsal hippocampus attenuate forward trace, backward trace, and delay fear conditioning in a temporally specific manner. Hippocampus 12, 495–504. https://doi.org/10.1002/hipo.10029

Quinn, J.J., Wied, H.M., Ma, Q.D., Tinsley, M.R., Fanselow, M.S., 2008. Dorsal hippocampus involvement in delay fear conditioning depends upon the strength of the tone‐footshock association. Hippocampus 18, 640–654. https://doi.org/10.1002/hipo.20424

Quiroga, R.Q., Kraskov, A., Koch, C., Fried, I., 2009. Explicit Encoding of Multimodal Percepts by Single Neurons in the Human Brain. Current Biology 19, 1308–1313. https://doi.org/10.1016/j.cub.2009.06.060

Rabelo, C., Weihing, J., Schochat, E., 2015. Temporal resolution in individuals with neurological disorders. Clinics 70, 606–611. https://doi.org/10.6061/clinics/2015(09)02

Radulovački, M., Adey, W.R., 1965. The hippocampus and the orienting reflex. Experimental Neurology 12, 68–83. https://doi.org/10.1016/0014-4886(65)90099-3

Rajasekaran, A., Shivashankar, N., Satish Chandra, P., Sinha, S., Saini, J., Subbakrishna, D.K., 2014. Dichotic perception in patients with and without medial temporal sclerosis. Speech, Language and Hearing 17, 153–159. https://doi.org/10.1179/2050572814Y.0000000034

Ramos-Escobar, N., Mercier, M., Trébuchon-Fonséca, A., Rodriguez-Fornells, A., François, C., Schön, D., 2022. Hippocampal and auditory contributions to speech segmentation. Cortex 150, 1–11. https://doi.org/10.1016/j.cortex.2022.01.017

Ransome, M.I., Turnley, A.M., 2008. Growth hormone signaling and hippocampal neurogenesis: Insights from genetic models. Hippocampus 18, 1034–1050. https://doi.org/10.1002/hipo.20463

Rausch, R., Crandall, P.H., 1982. Psychological Status Related to Surgical Control of Temporal Lobe Seizures. Epilepsia 23, 191–202. https://doi.org/10.1111/j.1528-1157.1982.tb05067.x

Raybuck, J.D., Lattal, K.M., 2014. Bridging the interval: Theory and neurobiology of trace conditioning. Behavioural Processes 101, 103–111. https://doi.org/10.1016/j.beproc.2013.08.016

Recasens, M., Gross, J., Uhlhaas, P.J., 2018. Low-Frequency Oscillatory Correlates of Auditory Predictive Processing in Cortical-Subcortical Networks: A MEG-Study. Scientific Reports 8. https://doi.org/10.1038/s41598-018-32385-3

Redding, F.K., 1967. Modification of sensory cortical evoked potentials by hippocampal stimulation. Electroencephalography and Clinical Neurophysiology 22, 74–83. https://doi.org/10.1016/0013-4694(67)90009-0

Reichel, J.M., Bedenk, B.T., Gassen, N.C., Hafner, K., Bura, S.A., Almeida-Correa, S., Genewsky, A., Dedic, N., Giesert, F., Agarwal, A., Nave, K.-A., Rein, T., Czisch, M., Deussing, J.M., Wotjak, C.T., 2016. Beware of your Cre-Ation: lacZ expression impairs neuronal integrity and hippocampus-dependent memory. Hippocampus 26, 1250–1264. https://doi.org/10.1002/hipo.22601

Rekart, J.L., Meiri, K., Routtenberg, A., 2005. Hippocampal-dependent memory is impaired in heterozygous GAP-43 knockout mice. Hippocampus 15, 1–7. https://doi.org/10.1002/hipo.20045

Ren, Y., Nguyen, V.T., Sonkusare, S., Lv, J., Pang, T., Guo, L., Eickhoff, S.B., Breakspear, M., Guo, C.C., 2018. Effective connectivity of the anterior hippocampus predicts recollection confidence during natural memory retrieval. Nat Commun 9, 4875. https://doi.org/10.1038/s41467-018-07325-4

Rey, C., Reinecke, H., Besser, R., 1984. Methyltin intoxication in six men; toxicologic and clinical aspects. Vet Hum Toxicol 26, 121–122.

Riaz, S., Schumacher, A., Sivagurunathan, S., Van Der Meer, M., Ito, R., 2017. Ventral, but not dorsal, hippocampus inactivation impairs reward memory expression and retrieval in contexts defined by proximal cues: RIAZ et al. Hippocampus 27, 822–836. https://doi.org/10.1002/hipo.22734

Richards, W., 1973. Time reproductions by H.M. Acta Psychologica 37, 279–282. https://doi.org/10.1016/0001-6918(73)90020-6

Richmond, M.A., Nichols, B.P., Deacon, R.M.J., Rawlins, J.N.P., 1997. Effects of Scopolamine and Hippocampal Lesions on Negative Patterning Discrimination Performance in Rats. Behavioral Neuroscience 111, 17–1227.

Rickert, E.J., Bennett, T.L., Lane, P., French, J., 1978. Hippocampectomy and the attenuation of blocking. Behavioral Biology 22, 147–160. https://doi.org/10.1016/S0091-6773(78)92170-3

Rickert, E.J., Lorden, J.F., Dawson, R., Smyly, E., 1981. Limbic Lesions and the Blocking Effect. Physiology & Behavior 26, 601–606.

Rickert, E.J., Lorden, J.F., Dawson, R., Smyly, E., Callahan, M.F., 1979. Stimulus processing and stimulus selection in rats with hippocampal lesions. Behavioral and Neural Biology 27, 454–465. https://doi.org/10.1016/S0163-1047(79)92040-5

Ricobaraza, A., Cuadrado-Tejedor, M., Marco, S., Pérez-Otaño, I., García-Osta, A., 2012. Phenylbutyrate rescues dendritic spine loss associated with memory deficits in a mouse model of Alzheimer disease. Hippocampus 22, 1040–1050. https://doi.org/10.1002/hipo.20883

Riddell, W.I., Rothblat, L.A., Wilson, W.A., 1969. Auditory and visual distraction in hippocampectomized rats. Journal of Comparative and Physiological Psychology 67, 216–219.

Robinson, S., Bucci, D.J., 2012. Fear conditioning is disrupted by damage to the postsubiculum. Hippocampus 22, 1481–1491. https://doi.org/10.1002/hipo.20987

Rocchi, F., Oya, H., Balezeau, F., Billig, A.J., Kocsis, Z., Jenison, R., Nourski, K.V., Kovach, C.K., Steinschneider, M., Kikuchi, Y., Rhone, A.E., Dlouhy, B.J., Kawasaki, H., Adolphs, R., Greenlee, J.D.W., Griffiths, T.D., Howard, M.A., Petkov, C.I., 2021. Common Fronto-temporal Effective Connectivity in Humans and Monkeys. Neuron 109, 1–17. https://doi.org/10.1101/2020.04.03.024042

Roesler, R., Kuyven, C.R., Kruel, A.V.S., Quevedo, J., Ferreira, M.B.C., 1998. Involvement of hippocampal NMDA receptors in retention of shuttle avoidance conditioning in rats. Brazilian Journal of Medical and Biological Research 31, 1601–1604. https://doi.org/10.1590/S0100-879X1998001200014

Rogozea, R., Ungher, J., 1968. Changes in orienting activity of cat induced by chronic hippocampal lesions. Experimental Neurology 21, 176–186. https://doi.org/10.1016/0014-4886(68)90136-2

Roman, R., Brázdil, M., Chládek, J., Rektor, I., Jurák, P., Světlák, M., Damborská, A., Shaw, D.J., Kukleta, M., 2013. Hippocampal negative event-related potential recorded in humans during a simple sensorimotor task occurs independently of motor execution: Hippocampal Event-Related Potential in Sensorimotor Task. Hippocampus 23, 1337–1344. https://doi.org/10.1002/hipo.22173

Romanski, L.M., LeDoux, J.E., 1993. Information Cascade from Primary Auditory Cortex to the Amygdala: Corticocortical and Corticoamygdaloid Projections of Temporal Cortex in the Rat. Cerebral Cortex 3, 515–532. https://doi.org/10.1093/cercor/3.6.515

Room, P., Groenewegen, H.J., 1986a. Connections of the parahippocampal cortex. I. Cortical afferents. The Journal of Comparative Neurology 251, 415–450. https://doi.org/10.1002/cne.902510402

Room, P., Groenewegen, H.J., 1986b. Connections of the parahippocampal cortex in the cat. II. Subcortical afferents. The Journal of Comparative Neurology 251, 451–473. https://doi.org/10.1002/cne.902510403

Rosburg, T., Trautner, P., Ludowig, E., Schaller, C., Kurthen, M., Elger, C.E., Boutros, N.N., 2007. Hippocampal event-related potentials to tone duration deviance in a passive oddball paradigm in humans. NeuroImage 37, 274–281. https://doi.org/10.1016/j.neuroimage.2007.05.002

Ross, R.T., Orr, W.B., Holland, P.C., Berger, T.W., 1984. Hippocampectomy Disrupts Acquisition and Retention of Learned Conditional Responding. Behavioral Neuroscience 98, 211–225.

Rotarska-Jagiela, A., van de Ven, V., Oertel-Knöchel, V., Uhlhaas, P.J., Vogeley, K., Linden, D.E.J., 2010. Resting-state functional network correlates of psychotic symptoms in schizophrenia. Schizophrenia Research 117, 21–30. https://doi.org/10.1016/j.schres.2010.01.001

Rothschild, G., Eban, E., Frank, L.M., 2017. A cortical–hippocampal–cortical loop of information processing during memory consolidation. Nature Neuroscience 20, 251–259. https://doi.org/10.1038/nn.4457

Rubin, R.D., Brown-Schmidt, S., Duff, M.C., Tranel, D., Cohen, N.J., 2011. How Do I Remember That I Know You Know That I Know? Psychological Science 22, 1574–1582. https://doi.org/10.1177/0956797611418245

Rudy, J.W., Sutherland, R.J., 1989. The hippocampal formation is necessary for rats to learn and remember configural discriminations. Behavioural Brain Research 34, 97–109. https://doi.org/10.1016/S0166-4328(89)80093-2

Rummell, B.P., Klee, J.L., Sigurdsson, T., 2016. Attenuation of Responses to Self-Generated Sounds in Auditory Cortical Neurons. The Journal of Neuroscience 36, 12010–12026. https://doi.org/10.1523/JNEUROSCI.1564-16.2016

Ruusuvirta, T., Astikainen, P., Wikgren, J., Nokia, M., 2010. Hippocampus responds to auditory change in rabbits. Neuroscience 170, 232–237. https://doi.org/10.1016/j.neuroscience.2010.06.062

Ruusuvirta, T., Korhonen, T., Arikoski, J., Kivirikko, K., 1996. ERPs to pitch changes: A result of reduced responses to standard tones in rabbits. NeuroReport 7, 413–416.

Ruusuvirta, T., Korhonen, T., Penttonen, M., Arikoski, J., 1995a. Hippocampal evoked potentials to pitch deviances in an auditory oddball situation in the rabbit: no human mismatch-like dependence on standard stimuli. Neuroscience Letters 185, 123–126. https://doi.org/10.1016/0304-3940(94)11240-J

Ruusuvirta, T., Korhonen, T., Penttonen, M., Arikoski, J., Kivirikko, K., 1995b. Hippocampal event-related potentials to pitch deviances in an auditory oddball situation in the cat: Experiment I. International Journal of Psychophysiology 20, 33–39. https://doi.org/10.1016/0167-8760(95)00024-M

Ruusuvirta, T., Korhonen, T., Penttonen, M., Arikoski, J., Kivirikko, K., 1995c. Behavioral and hippocampal evoked responses in an auditory oddball situation when an unconditioned stimulus is paired with deviant tones in the cat: Experiment II. International Journal of Psychophysiology 20, 41–47. https://doi.org/10.1016/0167-8760(95)00025-N

Ruusuvirta, T., Lipponen, A., Pellinen, E., Penttonen, M., Astikainen, P., 2013. Auditory Cortical and Hippocampal-System Mismatch Responses to Duration Deviants in Urethane-Anesthetized Rats. PLoS ONE 8, e54624. https://doi.org/10.1371/journal.pone.0054624

Ruusuvirta, T., Lipponen, A., Pellinen, E.-K., Penttonen, M., Astikainen, P., 2015. Auditory cortical and hippocampal local-field potentials to frequency deviant tones in urethane-anesthetized rats: An unexpected role of the sound frequencies themselves. International Journal of Psychophysiology 96, 134–140. https://doi.org/10.1016/j.ijpsycho.2015.04.007

Ryou, J.-W., Cho, S.-Y., Kim, H.-T., 2001. Lesions of the Entorhinal Cortex Impair Acquisition of Hippocampal-Dependent Trace Conditioning. Neurobiology of Learning and Memory 75, 121–127. https://doi.org/10.1006/nlme.2000.3966

Sacchetti, B., Lorenzini, C.A., Baldi, E., Tassoni, G., Bucherelli, C., 1999. Auditory Thalamus, Dorsal Hippocampus, Basolateral Amygdala, and Perirhinal Cortex Role in the Consolidation of Conditioned Freezing to Context and to Acoustic Conditioned Stimulus in the Rat. The Journal of Neuroscience 19, 9570–9578. https://doi.org/10.1523/JNEUROSCI.19-21-09570.1999

Sainsbury, R.S., Heynen, A., Montoya, C.P., 1987. Behavioral correlates of hippocampal type 2 theta in the rat. Physiology & Behavior 39, 513–519. https://doi.org/10.1016/0031-9384(87)90382-9

Sainsbury, R.S., Montoya, C.P., 1984. The relationship between type 2 theta and behavior. Physiology & Behavior 33, 621–626. https://doi.org/10.1016/0031-9384(84)90381-0

Sakimoto, Y., Hattori, M., Takeda, K., Okada, K., Sakata, S., 2013a. Hippocampal theta wave activity during configural and non-configural tasks in rats. Exp Brain Res 225, 177–185. https://doi.org/10.1007/s00221-012-3359-2

Sakimoto, Y., Okada, K., Hattori, M., Takeda, K., Sakata, S., 2013b. Neural activity in the hippocampus during conflict resolution. Behavioural Brain Research 237, 1–6. https://doi.org/10.1016/j.bbr.2012.09.013

Sakimoto, Y., Okada, K., Takeda, K., Sakata, S., 2013c. Transient Decline in Hippocampal Theta Activity during the Acquisition Process of the Negative Patterning Task. PLoS ONE 8, e70756. https://doi.org/10.1371/journal.pone.0070756

Sakimoto, Y., Sakata, S., 2015a. The transient decline in hippocampal theta power during response inhibition in a positive patterning task. NeuroReport 26, 833–837. https://doi.org/10.1097/WNR.0000000000000432

Sakimoto, Y., Sakata, S., 2015b. Change in hippocampal theta activity during behavioral inhibition for a stimulus having an overlapping element. Behavioural Brain Research 282, 111–116. https://doi.org/10.1016/j.bbr.2014.12.041

Sakimoto, Y., Sakata, S., 2015c. Behavioral inhibition during a conflict state elicits a transient decline in hippocampal theta power. Behavioural Brain Research 290, 70–76. https://doi.org/10.1016/j.bbr.2015.03.060

Sakimoto, Y., Sakata, S., 2014a. Hippocampal theta activity during behavioral inhibition for conflicting stimuli. Behavioural Brain Research 275, 183–190. https://doi.org/10.1016/j.bbr.2014.08.063

Sakimoto, Y., Sakata, S., 2014b. Change in hippocampal theta activity with transfer from simple discrimination tasks to a simultaneous feature-negative task. Front. Behav. Neurosci. 8. https://doi.org/10.3389/fnbeh.2014.00159

Sakimoto, Y., Sakata, S., 2013. The decline in rat hippocampal theta activity during response inhibition for the compound stimulus of negative patterning and simultaneous feature-negative tasks. Behavioural Brain Research 257, 111–117. https://doi.org/10.1016/j.bbr.2013.09.020

Sakimoto, Y., Takeda, K., Okada, K., Hattori, M., Sakata, S., 2013d. Transient decline in rats’ hippocampal theta power relates to inhibitory stimulus-reward association. Behavioural Brain Research 246, 132–138. https://doi.org/10.1016/j.bbr.2013.02.012

Sakurai, Y., 2002. Coding of auditory temporal and pitch information by hippocampal individual cells and cell assemblies in the rat. Neuroscience 115, 1153–1163. https://doi.org/10.1016/S0306-4522(02)00509-2

Sakurai, Y., 1996. Hippocampal and neocortical cell assemblies encode memory processes for different types of stimuli in the rat. The Journal of Neuroscience 16, 2809–2819. https://doi.org/10.1523/JNEUROSCI.16-08-02809.1996

Sakurai, Y., 1994. Involvement of auditory cortical and hippocampal neurons in auditory working memory and reference memory in the rat. The Journal of Neuroscience 14, 2606–2623. https://doi.org/10.1523/JNEUROSCI.14-05-02606.1994

Sakurai, Y., 1990. Hippocampal Cells Have Behavioral Correlates During the Performance of an Auditory Working Memory Task in the Rat 11.

Salafia, W.R., Chiaia, N.L., Ramirez, J.J., 1979. Retardation of rabbit nictitating membrane conditioning by subseizure electrical stimulation of hippocampus. Physiology & Behavior 22, 451–455. https://doi.org/10.1016/0031-9384(79)90008-8

Salafia, W.R., Romano, A.G., Tynan, T., Host, K.C., 1977. Disruption of rabbit (Oryctolagus cuniculus) nictitating membrane conditioning by posttrial electrical stimulation of hippocampus. Physiology & Behavior 18, 207–212. https://doi.org/10.1016/0031-9384(77)90123-8

Salimpoor, V.N., van den Bosch, I., Kovacevic, N., McIntosh, A.R., Dagher, A., Zatorre, R.J., 2013. Interactions Between the Nucleus Accumbens and Auditory Cortices Predict Music Reward Value. Science 340, 216–219. https://doi.org/10.1126/science.1231059

Säljö, A., Bao, F., Haglid, K.G., Hansson, H.-A., 2000. Blast Exposure Causes Redistribution of Phosphorylated Neurofilament Subunits in Neurons of the Adult Rat Brain. Journal of Neurotrauma 17, 719–726. https://doi.org/10.1089/089771500415454

Säljö, A., Bao, F., Jingshan, S., Hamberger, A., Hansson, H.-A., Haglid, K.G., 2002. Exposure to Short-Lasting Impulse Noise Causes Neuronal c-Jun Expression and Induction of Apoptosis in the Adult Rat Brain. Journal of Neurotrauma 19, 985–991. https://doi.org/10.1089/089771502320317131

Salvatierra, A.T., Berry, S.D., 1989. Scopolamine Disruption of Septo-Hippocampal Activity and Classical Conditioning. Behavioural Neuroscience 103, 715–721.

Samson, S., Ehrlé, N., Baulac, M., 2001. Cerebral Substrates for Musical Temporal Processes. Annals of the New York Academy of Sciences 930, 166–178.

Samson, S., Peretz, I., 2005. Effects of Prior Exposure on Music Liking and Recognition in Patients with Temporal Lobe Lesions. Annals of the New York Academy of Sciences 1060, 419–428. https://doi.org/10.1196/annals.1360.035

Samson, S., Zatorre, R.J., 1994. Contribution of the right temporal lobe to musical timbre discrimination. Neuropsychologia 32, 231–240. https://doi.org/10.1016/0028-3932(94)90008-6

Samson, S., Zatorre, R.J., 1992. Learning and retention of melodic and verbal information after unilateral temporal lobectomy. Neuropsychologia 30, 815–826. https://doi.org/10.1016/0028-3932(92)90085-Z

Samson, S., Zatorre, R.J., 1991. Recognition Memory for Text and Melody of Songs After Unilateral Temporal Lobe Lesion Evidence for Dual Encoding. Journal of Experimental Psychology: Learning, Memory, and Cognition 17, 793–804.

Sanwald, J.C., Porzio, N.R., Deane, G.E., Donovick, P.J., 1970. The effects of septal and dorsal hippocampal lesions on the cardiac component of the orienting response. Physiology & Behavior 5, 883–888. https://doi.org/10.1016/0031-9384(70)90176-9

Sanyal, T., Kumar, V., Nag, T.C., Jain, S., Sreenivas, V., Wadhwa, S., 2013a. Prenatal Loud Music and Noise: Differential Impact on Physiological Arousal, Hippocampal Synaptogenesis and Spatial Behavior in One Day-Old Chicks. PLoS ONE 8, e67347. https://doi.org/10.1371/journal.pone.0067347

Sanyal, T., Palanisamy, P., Nag, T.C., Roy, T.S., Wadhwa, S., 2013b. Effect of prenatal loud music and noise on total number of neurons and glia, neuronal nuclear area and volume of chick brainstem auditory nuclei, field L and hippocampus: A stereological investigation. International Journal of Developmental Neuroscience 31, 234–244. https://doi.org/10.1016/j.ijdevneu.2013.02.004

Saykin, A.J., Johnson, S.C., Flashman, L.A., McAllister, T.W., Sparling, M., Darcey, T.M., Moritz, C.H., Guerin, S.J., Weaver, J., Mamourian, A., 1999. Functional differentiation of medial temporal and frontal regions involved in processing novel and familiar words: an fMRI study. Brain 122, 1963–1971. https://doi.org/10.1093/brain/122.10.1963

Schapiro, A.C., Gregory, E., Landau, B., McCloskey, M., Turk-Browne, N.B., 2014. The Necessity of the Medial Temporal Lobe for Statistical Learning. Journal of Cognitive Neuroscience 26, 1736–1747. https://doi.org/10.1162/jocn_a_00578

Schecklmann, M., Landgrebe, M., Poeppl, T.B., Kreuzer, P., Männer, P., Marienhagen, J., Wack, D.S., Kleinjung, T., Hajak, G., Langguth, B., 2013. Neural correlates of tinnitus duration and Distress: A positron emission tomography study. Human Brain Mapping 34, 233–240. https://doi.org/10.1002/hbm.21426

Schimanski, L.A., Wahlsten, D., Nguyen, P.V., 2002. Selective Modification of Short-Term Hippocampal Synaptic Plasticity and Impaired Memory Extinction in Mice with a Congenitally Reduced Hippocampal Commissure. The Journal of Neuroscience 22, 8277–8286. https://doi.org/10.1523/JNEUROSCI.22-18-08277.2002

Schmaltz, L.W., Theios, J., 1972. Acquisition and extinction of a classically conditioned response in hippocampectomized rabbits (Oryctolaous Cuniculus). Journal of Comparative and Physiological Psychology 79, 328–333.

Schmithorst, V.J., 2005. Separate cortical networks involved in music perception: preliminary functional MRI evidence for modularity of music processing. NeuroImage 25, 444–451. https://doi.org/10.1016/j.neuroimage.2004.12.006

Schmithorst, V.J., Holland, S.K., Plante, E., 2006. Cognitive modules utilized for narrative comprehension in children: a functional magnetic resonance imaging study. NeuroImage 29, 254–266. https://doi.org/10.1016/j.neuroimage.2005.07.020

Schreurs, B.G., Mcintosh, A.R., Bahro, M., Herscovitch, P., Sunderland, T., Molchan, S.E., 1997. Lateralization and Behavioral Correlation of Changes in Regional Cerebral Blood Flow With Classical Conditioning of the Human Eyeblink Response. Journal of Neurophysiology 77, 2153–2163. https://doi.org/10.1152/jn.1997.77.4.2153

Schwartzbaum, J.S., Thompson, J.B., Kellicutt, M.H., 1964. Auditory frequency discrimination and generalization following lesions of the amygdaloid area in rats. Journal of Comparative and Physiological Psychology 57, 257–266. https://doi.org/10.1037/h0039921

Schwenzer, M., Mathiak, K., 2011. Numeric aspects in pitch identification: an fMRI study. BMC Neuroscience 12. https://doi.org/10.1186/1471-2202-12-26

Scoville, W.B., Milner, B., 1957. Loss of recent memory after bilateral hippocampal lesions. Journal of Neurology, Neurosurgery & Psychiatry 20, 11–21.

Segal, M., 1974. Convergence of sensory input on units in the hippocampal system of the rat. Journal of Comparative and Physiological Psychology 87, 91–99. https://doi.org/10.1037/h0036581

Segal, M., 1973. Flow of conditioned responses in limbic telencephalic system of the rat. Journal of Neurophysiology 36, 840–854. https://doi.org/10.1152/jn.1973.36.5.840

Segal, M., Disterhoft, J.F., Olds, J., 1972. Hippocampal Unit Activity during Classical Aversive and Appetitive Conditioning. Science 175, 792–794. https://doi.org/10.1126/science.175.4023.792

Segal, M., Olds, J., 1973. Activity of units in the hippocampal circuit of the rat during differential classical conditioning. Journal of Comparative and Physiological Psychology 82, 195–204. https://doi.org/10.1037/h0033912

Segal, M., Olds, J., 1972. Behavior of units in hippocampal circuit of the rat during learning. Journal of Neurophysiology 35, 680–690. https://doi.org/10.1152/jn.1972.35.5.680

Sekeres, M.J., Winocur, G., Moscovitch, M., Anderson, J.A.E., Pishdadian, S., Martin Wojtowicz, J., St-Laurent, M., McAndrews, M.P., Grady, C.L., 2018. Changes in patterns of neural activity underlie a time-dependent transformation of memory in rats and humans. Hippocampus 28, 745–764. https://doi.org/10.1002/hipo.23009

Sellami, A., Al Abed, A.S., Brayda-Bruno, L., Etchamendy, N., Valério, S., Oulé, M., Pantaléon, L., Lamothe, V., Potier, M., Bernard, K., Jabourian, M., Herry, C., Mons, N., Piazza, P.-V., Eichenbaum, H., Marighetto, A., 2017. Temporal binding function of dorsal CA1 is critical for declarative memory formation. Proceedings of the National Academy of Sciences 114, 10262–10267. https://doi.org/10.1073/pnas.1619657114

Seltzer, B., Pandya, D.N., 1976. Some cortical projections to the parahippocampal area in the rhesus monkey. Experimental Neurology 50, 146–160.

Senba, K., Iwahara, S., 1974. Effects of medial septal lesions on the hippocampal electrical activity and the orienting response to auditory stimulation in drinking rats. Brain Research 66, 309–320. https://doi.org/10.1016/0006-8993(74)90149-8

Seo, D.-O., Pang, M.-H., Shin, M.-S., Kim, H.-T., Choi, J.-S., 2008. Hippocampal NMDA receptors are necessary for auditory trace fear conditioning measured with conditioned hypoalgesia in rats. Behavioural Brain Research 192, 264–268. https://doi.org/10.1016/j.bbr.2008.04.011

Shan, K.Q., Lubenov, E.V., Papadopoulou, M., Siapas, A.G., 2016. Spatial tuning and brain state account for dorsal hippocampal CA1 activity in a non-spatial learning task. eLife 5, e14321. https://doi.org/10.7554/eLife.14321

Shen, J., Yao, P.-T., Ge, S., Xiong, Q., 2021. Dentate granule cells encode auditory decisions after reinforcement learning in rats. Sci Rep 11, 14360. https://doi.org/10.1038/s41598-021-93721-8

Shen, L., Nam, H.-S., Song, P., Moore, H., Anderson, S.A., 2006. FoxG1 haploinsufficiency results in impaired neurogenesis in the postnatal hippocampus and contextual memory deficits. Hippocampus 16, 875–890. https://doi.org/10.1002/hipo.20218

Shen, Y., Hu, H., Fan, C., Wang, Q., Zou, T., Ye, B., Xiang, M., 2021. Sensorineural hearing loss may lead to dementia-related pathological changes in hippocampal neurons. Neurobiology of Disease 105408. https://doi.org/10.1016/j.nbd.2021.105408

Shergill, S.S., Brammer, M.J., Williams, S.C.R., Murray, R.M., McGuire, P.K., 2000. Mapping Auditory Hallucinations in Schizophrenia Using Functional Magnetic Resonance Imaging. Archives of General Psychiatry 57, 1033. https://doi.org/10.1001/archpsyc.57.11.1033

Shergill, S.S., Cameron, L.A., Brammer, M.J., Williams, S.C., Murray, R.M., McGuire, P.K., 2001. Modality specific neural correlates of auditory and somatic hallucinations. J Neurol Neurosurg Psychiatry 71, 688–690. https://doi.org/10.1136/jnnp.71.5.688

Shin, J., 2011. The interrelationship between movement and cognition: Theta rhythm and the P300 event-related potential. Hippocampus 21, 744–752. https://doi.org/10.1002/hipo.20792

Shinba, T., 1999. Neuronal firing activity in the dorsal hippocampus during the auditory discrimination oddball task in awake rats: relation to event-related potential generation. Cognitive Brain Research 8, 241–250. https://doi.org/10.1016/S0926-6410(99)00026-9

Shinba, T., Andow, Y., Shinozaki, T., Ozawa, N., Yamamoto, K., 1996. Event-related potentials in the dorsal hippocampus of rats during an auditory discrimination paradigm. Electroencephalography and clinical Neurophysiology 6.

Shinkman, P.G., Kaufman, K.P., 1972a. Posttrial hippocampal stimulation and CER acquisition in the rat. Journal of Comparative and Physiological Psychology 80, 283–292. https://doi.org/10.1037/h0032980

Shinkman, P.G., Kaufman, K.P., 1972b. Time course of retroactive effects of hippocampal stimulation on learning. Experimental Neurology 34, 476–483. https://doi.org/10.1016/0014-4886(72)90043-X

Shinn, A.K., Baker, J.T., Cohen, B.M., Öngür, D., 2013. Functional connectivity of left Heschl’s gyrus in vulnerability to auditory hallucinations in schizophrenia. Schizophrenia Research 143, 260–268. https://doi.org/10.1016/j.schres.2012.11.037

Shoemaker, J.M., Saint Marie, R.L., Bongiovanni, M.J., Neary, A.C., Tochen, L.S., Swerdlow, N.R., 2005. Prefrontal D1 and ventral hippocampal N-methyl-d-aspartate regulation of startle gating in rats. Neuroscience 135, 385–394. https://doi.org/10.1016/j.neuroscience.2005.06.054

Shors, T.J., Miesegaes, G., Beylin, A., Zhao, M., Rydel, T., Gould, E., 2001. Neurogenesis in the adult is involved in the formation of trace memories. Nature 410, 372–376. https://doi.org/10.1038/35066584

Shulman, A., Strashun, A.M., Afriyie, M., Aronson, F., Abel, W., Goldstein, B., 1995. SPECT imaging of brain and tinnitus - neurotologic/neurologic implications. International Tinnitus Journal 1, 13–29.

Sierra-Mercado, D., Padilla-Coreano, N., Quirk, G.J., 2011. Dissociable Roles of Prelimbic and Infralimbic Cortices, Ventral Hippocampus and Basolateral Amygdala in the Expression and Extinction of Conditioned Fear. Neuropsychopharmacology 36, 529–538. https://doi.org/10.1038/npp.2010.184

Silbersweig, D.A., Stern, E., Frith, C., Cahill, C., Holmes, A., Grootoonk, S., Seaward, J., McKenna, P., Chua, S.E., Schnorr, L., Jones, T., Frackowiak, R.S.J., 1995. A functional neuroanatomy of hallucinations in schizophrenia. Nature 378.

Simonetti, P., Ono, C.R., Godoi Carneiro, C. de, Ali Khan, R., Shahsavarani, S., Husain, F.T., Oiticica, J., 2022. Evaluating the efficacy of hearing aids for tinnitus therapy – A Positron emission tomography study. Brain Research 1775, 147728. https://doi.org/10.1016/j.brainres.2021.147728

Sinclair, B.R., Seto, M.G., Bland, B.H., 1982. theta-Cells in CA1 and dentate layers of hippocampal formation: relations to slow-wave activity and motor behavior in the freely moving rabbit. Journal of Neurophysiology 48, 1214–1225. https://doi.org/10.1152/jn.1982.48.5.1214

Singer, W., Zuccotti, A., Jaumann, M., Lee, S.C., Panford-Walsh, R., Xiong, H., Zimmermann, U., Franz, C., Geisler, H.-S., Köpschall, I., Rohbock, K., Varakina, K., Verpoorten, S., Reinbothe, T., Schimmang, T., Rüttiger, L., Knipper, M., 2013. Noise-Induced Inner Hair Cell Ribbon Loss Disturbs Central Arc Mobilization: A Novel Molecular Paradigm for Understanding Tinnitus. Molecular Neurobiology 47, 261–279. https://doi.org/10.1007/s12035-012-8372-8

Skouras, S., Gray, M., Critchley, H., Koelsch, S., 2014. Superficial amygdala and hippocampal activity during affective music listening observed at 3 T but not 1.5 T fMRI. NeuroImage 101, 364–369. https://doi.org/10.1016/j.neuroimage.2014.07.007

Sliwa, J., Planté, A., Duhamel, J.-R., Wirth, S., 2014. Independent Neuronal Representation of Facial and Vocal Identity in the Monkey Hippocampus and Inferotemporal Cortex. Cerebral Cortex 26, 950–966. https://doi.org/10.1093/cercor/bhu257

Small, S.A., Nava, A.S., Perera, G.M., DeLaPaz, R., Mayeux, R., Stern, Y., 2001. Circuit mechanisms underlying memory encoding and retrieval in the long axis of the hippocampal formation. Nature Neuroscience 4, 442–449. https://doi.org/10.1038/86115

Smith, C.N., Urgolites, Z.J., Hopkins, R.O., Squire, L.R., 2014. Comparison of explicit and incidental learning strategies in memory-impaired patients. Proceedings of the National Academy of Sciences 111, 475–479. https://doi.org/10.1073/pnas.1322263111

Smith, D.M., Wakeman, D., Patel, J., Gabriel, M., 2004. Fornix Lesions Impair Context-Related Cingulothalamic Neuronal Patterns and Concurrent Discrimination Learning in Rabbits (Oryctolagus cuniculus). Behavioral Neuroscience 118, 1225–1239. https://doi.org/10.1037/0735-7044.118.6.1225

Smith, M.E., Halgren, E., Sokolik, M., Baudena, P., Musolino, A., Liegeois-Chauvel, C., Chauvel, P., 1990. The intracranial topography of the P3 event-related potential elicited during auditory oddball. Electroencephalography and Clinical Neurophysiology 76, 235–248. https://doi.org/10.1016/0013-4694(90)90018-F

Smith, M.E., Stapleton, J.M., Halgren, E., 1986. Human medial temporal lobe potentials evoked in memory and language tasks. Electroencephalography and Clinical Neurophysiology 63, 145–159.

Solomon, P.R., 1977. Role of the hippocampus in blocking and conditioned inhibition of the rabbit’s nictitating membrane response. Journal of Comparative and Physiological Psychology 91, 407–417. https://doi.org/10.1037/h0077330

Solomon, P.R., Moore, J.W., 1975. Latent Inhibition and Stimulus Generalization of the Classically Conditioned Nictitating Membrane Response in Rabbits (Oryctolagus cuniculus) Following Dorsal Hippocampal Ablation. Journal of Comparative and Physiological Psychology 89, 1192–1203.

Solomon, P.R., Schaaf, E.R.V., College, W., Thompson, R.F., Weisz, D.J., 1986. Hippocampus and Trace Conditioning of the Rabbit’s Classically Conditioned Nictitating Membrane Response. Behavioral Neuroscience 100, 729–744.

Sommer, I.E., Clos, M., Meijering, A.L., Diederen, K.M.J., Eickhoff, S.B., 2012. Resting State Functional Connectivity in Patients with Chronic Hallucinations. PLoS ONE 7, e43516. https://doi.org/10.1371/journal.pone.0043516

Squire, L.R., Schmolck, H., Stark, S.M., 2001. Impaired Auditory Recognition Memory in Amnesic Patients with Medial Temporal Lobe Lesions. Learning & Memory 8, 252–256. https://doi.org/10.1101/lm.42001

Squires, N.K., Halgren, E., Wilson, C., Crandall, P., 1983. Human Endogenous Limbic Potentials: Cross-Modality and Depth/Surface Comparisons in Epileptic Subjects, in: Advances in Psychology. Elsevier, pp. 217–232. https://doi.org/10.1016/S0166-4115(08)62041-5

Staib, J.M., Della Valle, R., Knox, D.K., 2018. Disruption of medial septum and diagonal bands of Broca cholinergic projections to the ventral hippocampus disrupt auditory fear memory. Neurobiology of Learning and Memory 152, 71–79. https://doi.org/10.1016/j.nlm.2018.05.009

Stefanacci, L., Buffalo, E.A., Schmolck, H., Squire, L.R., 2000. Profound Amnesia After Damage to the Medial Temporal Lobe: A Neuroanatomical and Neuropsychological Profile of Patient E. P. The Journal of Neuroscience 20, 7024–7036. https://doi.org/10.1523/JNEUROSCI.20-18-07024.2000

Stepien, L.S., Cordeau, P., Rasmussen, T., 1960. The effect of temporal lobe and hippocampal lesions on auditory and visual recent memory in monkeys. Brain 83, 470–489. https://doi.org/10.1093/brain/83.3.470

Stepien, L.S., Sierpinski, S., 1960. The effect of focal lesions of the brain upon auditory and visual recent memory in man. J Neurol Neurosurg Psychiat 23, 334–340.

Stevens, K.E., Freedman, R., Collins, A.C., Hall, M., Leonard, S., Marks, M.J., Rose, G.M., 1996. Genetic Correlation of Inhibitory Gating of Hippocampal Auditory Evoked Response and cx-Bungarotoxin-Binding Nicotinic Cholinergic Receptors in Inbred Mouse Strains 15, 11.

Stevens, K.E., Wear, K.D., 1997. Normalizing Effects of Nicotine and a Novel Nicotinic Agonist on Hippocampal Auditory Gating in Two Animal Models. Pharmacology Biochemistry and Behavior 57, 869–874. https://doi.org/10.1016/S0091-3057(96)00466-2

Stolar, N., Sparenborg, S., Donchin, E., Gabriel, M., 1989. Conditional Stimulus Probability and Activity of Hippocampal, Cingulate Cortical, and Limbic Thalamic Neurons During Avoidance Conditioning in Rabbits. Behavioral Neuroscience 103, 919–934.

Straube, B., Green, A., Weis, S., Chatterjee, A., Kircher, T., 2009. Memory Effects of Speech and Gesture Binding: Cortical and Hippocampal Activation in Relation to Subsequent Memory Performance. Journal of Cognitive Neuroscience 21, 821–836. https://doi.org/10.1162/jocn.2009.21053

Sun, M., Gewirtz, J.C., Bofenkamp, L., Wickham, R.J., Ge, H., O’Connor, M.B., 2010. Canonical TGF- Signaling Is Required for the Balance of Excitatory/Inhibitory Transmission within the Hippocampus and Prepulse Inhibition of Acoustic Startle. Journal of Neuroscience 30, 6025–6035. https://doi.org/10.1523/JNEUROSCI.0789-10.2010

Sundaramahalingam, M., Ramasundaram, S., Rathinasamy, S.D., Natarajan, R.P., Somasundaram, T., 2013. Role of Acorus calamus and α-asarone on Hippocampal Dependent Memory in Noise Stress Exposed Rats. Pakistan Journal of Biological Sciences 16, 770–778. https://doi.org/10.3923/pjbs.2013.770.778

Suzuki, K., Takei, N., Toyoda, T., Iwata, Y., Hoshino, R., Minabe, Y., Mori, N., 2003. Auditory hallucinations and cognitive impairment in a patient with a lesion restricted to the hippocampus. Schizophrenia Research 64, 87–89. https://doi.org/10.1016/S0920-9964(02)00386-9

Suzuki, W.L., Amaral, D.G., 1994. Perirhinal and parahippocampal cortices of the macaque monkey: Cortical afferents. The Journal of Comparative Neurology 350, 497–533. https://doi.org/10.1002/cne.903500402

Svoboda, E., Levine, B., 2009. The Effects of Rehearsal on the Functional Neuroanatomy of Episodic Autobiographical and Semantic Remembering: A Functional Magnetic Resonance Imaging Study. Journal of Neuroscience 29, 3073–3082. https://doi.org/10.1523/JNEUROSCI.3452-08.2009

Swanson, L., Köhler, C., 1986. Anatomical evidence for direct projections from the entorhinal area to the entire cortical mantle in the rat. The Journal of Neuroscience 6, 3010–3023. https://doi.org/10.1523/JNEUROSCI.06-10-03010.1986

Swerdlow, N.R., Light, G.A., Breier, M.R., Shoemaker, J.M., Saint Marie, R.L., Neary, A.C., Geyer, M.A., Stevens, K.E., Powell, S.B., 2012. Sensory and Sensorimotor Gating Deficits after Neonatal Ventral Hippocampal Lesions in Rats. Developmental Neuroscience 34, 240–249. https://doi.org/10.1159/000336841

Swerdlow, N.R., Powell, S.B., Breier, M.R., Hines, S.R., Light, G.A., 2013. Coupling of gene expression in medial prefrontal cortex and nucleus accumbens after neonatal ventral hippocampal lesions accompanies deficits in sensorimotor gating and auditory processing in rats. Neuropharmacology 75, 38–46. https://doi.org/10.1016/j.neuropharm.2013.06.003

Tae, W.-S., Yakunina, N., Lee, W.H., Ryu, Y.-J., Ham, H., Pyun, S.-B., Nam, E.-C., 2018. Changes in the regional shape and volume of subcortical nuclei in patients with tinnitus comorbid with mild hearing loss. Neuroradiology 60, 1203–1211. https://doi.org/10.1007/s00234-018-2093-2

Takahashi, S., Sakurai, Y., 2009. Sub-millisecond firing synchrony of closely neighboring pyramidal neurons in hippocampal CA1 of rats during delayed non-matching to sample task. Frontiers in Neural Circuits 3. https://doi.org/10.3389/neuro.04.009.2009

Takakura, H., Umeno, K., Tabuchi, E., Hori, E., Miyamoto, K., Aso, S., Watanabe, Y., Ono, T., Nishijo, H., 2003. Differential activation in the medial temporal lobe during a sound-sequence discrimination task across age in human subjects. Neuroscience 119, 517–532. https://doi.org/10.1016/S0306-4522(03)00193-3

Takamiya, S., Shiotani, K., Ohnuki, T., Osako, Y., Tanisumi, Y., Yuki, S., Manabe, H., Hirokawa, J., Sakurai, Y., 2021. Hippocampal CA1 Neurons Represent Positive Feedback During the Learning Process of an Associative Memory Task. Front. Syst. Neurosci. 15, 718619. https://doi.org/10.3389/fnsys.2021.718619

Takebayashi, H., Takei, N., Suzuki, S., Mori, N., 2002. Unilateral auditory hallucinations in schizophrenia after damage to the right hippocampus. Schizophrenia Research 58, 329–331. https://doi.org/10.1016/S0920-9964(01)00399-1

Takehara, K., Kawahara, S., Kirino, Y., 2003. Time-Dependent Reorganization of the Brain Components Underlying Memory Retention in Trace Eyeblink Conditioning. The Journal of Neuroscience 23, 9897–9905. https://doi.org/10.1523/JNEUROSCI.23-30-09897.2003

Takehara-Nishiuchi, K., Maal-Bared, G., Morrissey, M.D., 2012. Increased Entorhinal–Prefrontal Theta Synchronization Parallels Decreased Entorhinal–Hippocampal Theta Synchronization during Learning and Consolidation of Associative Memory. Front. Behav. Neurosci. 5. https://doi.org/10.3389/fnbeh.2011.00090

Talk, A., Grasby, K., Rawson, T., Ebejer, J., 2016. Preconditioning of Spatial and Auditory Cues: Roles of the Hippocampus, Frontal Cortex, and Cue-Directed Attention. Brain Sciences 6, 63. https://doi.org/10.3390/brainsci6040063

Talk, A.C., Gandhi, C.C., Matzel, L.D., 2002. Hippocampal function during behaviorally silent associative learning: Dissociation of memory storage and expression. Hippocampus 12, 648–656. https://doi.org/10.1002/hipo.10098

Talnov, A.N., Quiroga, R.Q., Meier, M., Matsumoto, G., Branka?k, J., 2003. Entorhinal inputs to dentate gyrus are activated mainly by conditioned events with long time intervals. Hippocampus 13, 755–765. https://doi.org/10.1002/hipo.10126

Tam, S.K.E., Bonardi, C., 2012. Dorsal hippocampal lesions disrupt Pavlovian delay conditioning and conditioned-response timing. Behavioural Brain Research 230, 259–267. https://doi.org/10.1016/j.bbr.2012.02.016

Tam, S.K.E., Jennings, D.J., Bonardi, C., 2015. Effects of dorsal hippocampal damage on conditioning and conditioned-response timing: A pooled analysis: Hippocampus and Response Timing. Hippocampus 25, 444–459. https://doi.org/10.1002/hipo.22381

Tamura, R., Ono, T., Fukuda, M., Nakamura, K., 1992. Spatial responsiveness of monkey hippocampal neurons to various visual and auditory stimuli. Hippocampus 2, 307–322. https://doi.org/10.1002/hipo.450020309

Tamura, R., Ono, T., Fukuda, M., Nakamura, K., 1990. Recognition of egocentric and allocentric visual and auditory space by neurons in the hippocampus of monkeys. Neuroscience Letters 109, 293–298. https://doi.org/10.1016/0304-3940(90)90010-7

Tang, J., Wagner, S., Schachner, M., Dityatev, A., Wotjak, C.T., 2003. Potentiation of amygdaloid and hippocampal auditory-evoked potentials in a discriminatory fear-conditioning task in mice as a function of tone pattern and context. European Journal of Neuroscience 18, 639–650. https://doi.org/10.1046/j.1460-9568.2003.02758.x

Tao, S., Liu, L., Shi, L., Li, X., Shen, P., Xun, Q., Guo, X., Yu, Z., Wang, J., 2015. Spatial learning and memory deficits in young adult mice exposed to a brief intense noise at postnatal age. Journal of Otology 10, 21–28. https://doi.org/10.1016/j.joto.2015.07.001

Teki, S., Griffiths, T.D., 2016. Brain Bases of Working Memory for Time Intervals in Rhythmic Sequences. Frontiers in Neuroscience 10. https://doi.org/10.3389/fnins.2016.00239

Terada, S., Sakurai, Y., Nakahara, H., Fujisawa, S., 2017. Temporal and Rate Coding for Discrete Event Sequences in the Hippocampus. Neuron 94, 1248-1262.e4. https://doi.org/10.1016/j.neuron.2017.05.024

Tesche, C.D., Karhu, J., 1999. Interactive Processing of Sensory Input and Motor Output in the Human Hippocampus. Journal of Cognitive Neuroscience 11, 424–436. https://doi.org/10.1162/089892999563517

Tesche, C.D., Karhu, J., Tissari, S.O., 1996. Non-invasive detection of neuronal population activity in human hippocampus. Cognitive Brain Research 4, 39–47. https://doi.org/10.1016/0926-6410(95)00044-5

Tezer, F.I., Ilhan, B., Erbil, N., Saygi, S., Akalan, N., Ungan, P., 2012. Lateralisation of sound in temporal-lobe epilepsy: Comparison between pre- and postoperative performances and ERPs. Clinical Neurophysiology 123, 2362–2369. https://doi.org/10.1016/j.clinph.2012.06.015

Thakral, P.P., Wang, T.H., Rugg, M.D., 2015. Cortical reinstatement and the confidence and accuracy of source memory. NeuroImage 109, 118–129. https://doi.org/10.1016/j.neuroimage.2015.01.003

Thomas, J.D., Tran, T.D., 2012. Choline supplementation mitigates trace, but not delay, eyeblink conditioning deficits in rats exposed to alcohol during development. Hippocampus 22, 619–630. https://doi.org/10.1002/hipo.20925

Thompson, L.T., Moyer, J.R., Disterhoft, J.F., 1996. Transient changes in excitability of rabbit CA3 neurons with a time course appropriate to support memory consolidation. Journal of Neurophysiology 76, 1836–1849. https://doi.org/10.1152/jn.1996.76.3.1836

Tobia, M.J., Iacovella, V., Hasson, U., 2012. Multiple sensitivity profiles to diversity and transition structure in non-stationary input. NeuroImage 60, 991–1005. https://doi.org/10.1016/j.neuroimage.2012.01.041

Tranel, D., Brady, D.R., Van Hoesen, G.W., Damasio, A.R., 1988. Parahippocampal projections to posterior auditory association cortex (area Tpt) in Old-World monkeys. Experimental Brain Research 70, 406–416. https://doi.org/10.1007/BF00248365

Trost, W., Ethofer, T., Zentner, M., Vuilleumier, P., 2012. Mapping Aesthetic Musical Emotions in the Brain. Cerebral Cortex 22, 2769–2783. https://doi.org/10.1093/cercor/bhr353

Tseng, W., Guan, R., Disterhoft, J.F., Weiss, C., 2004. Trace eyeblink conditioning is hippocampally dependent in mice. Hippocampus 14, 58–65. https://doi.org/10.1002/hipo.10157

Tulving, E., Habib, R., Nyberg, L., Lepage, M., McIntosh, A.R., 1999. Positron emission tomography correlations in and beyond medial temporal lobes. Hippocampus 9, 71–82. https://doi.org/10.1002/(SICI)1098-1063(1999)9:1<71::AID-HIPO8>3.0.CO;2-F

Turner, B.H., Mishkin, M., Knapp, M., 1980. Organization of the amygdalopetal projections from modality-specific cortical association areas in the monkey. The Journal of Comparative Neurology 191, 515–543. https://doi.org/10.1002/cne.901910402

Twining, R.C., Lepak, K., Kirry, A.J., Gilmartin, M.R., 2020. Ventral Hippocampal Input to the Prelimbic Cortex Dissociates the Context from the Cue Association in Trace Fear Memory. J. Neurosci. 40, 3217–3230. https://doi.org/10.1523/JNEUROSCI.1453-19.2020

Ueyama, T., Donishi, T., Ukai, S., Ikeda, Y., Hotomi, M., Yamanaka, N., Shinosaki, K., Terada, M., Kaneoke, Y., 2013. Brain Regions Responsible for Tinnitus Distress and Loudness: A Resting-State fMRI Study. PLoS ONE 8, e67778. https://doi.org/10.1371/journal.pone.0067778

Ungiadze, A.A., 1967. Electrical activity of the hippocampus during peripheral stimulation. Neuroscience Translations 1, 136–142. https://doi.org/10.1007/BF01124392

Uran, S.L., Aon-Bertolino, M.L., Caceres, L.G., Capani, F., Guelman, L.R., 2012. Rat hippocampal alterations could underlie behavioral abnormalities induced by exposure to moderate noise levels. Brain Research 1471, 1–12. https://doi.org/10.1016/j.brainres.2012.06.022

Uran, S.L., Caceres, L.G., Guelman, L.R., 2010. Effects of loud noise on hippocampal and cerebellar-related behaviors. Brain Research 1361, 102–114. https://doi.org/10.1016/j.brainres.2010.09.022

Uran, S.L., Gómez-Casati, M.E., Guelman, L.R., 2014. Long-term recovery from hippocampal-related behavioral and biochemical abnormalities induced by noise exposure during brain development. Evaluation of auditory pathway integrity. International Journal of Developmental Neuroscience 37, 41–51. https://doi.org/10.1016/j.ijdevneu.2014.06.002

Urgolites, Z.J., Wixted, J.T., Goldinger, S.D., Papesh, M.H., Treiman, D.M., Squire, L.R., Steinmetz, P.N., 2020. Spiking activity in the human hippocampus prior to encoding predicts subsequent memory. Proc Natl Acad Sci USA 117, 13767–13770. https://doi.org/10.1073/pnas.2001338117

van de Ven, V., Waldorp, L., Christoffels, I., 2020. Hippocampus plays a role in speech feedback processing. NeuroImage 223, 117319. https://doi.org/10.1016/j.neuroimage.2020.117319

Van Hoesen, G.W., Pandya, D.N., Butters, N., 1972. Cortical Afferents to the Entorhinal Cortex of the Rhesus Monkey. Science 175, 1471–1473. https://doi.org/10.1126/science.175.4029.1471

Vaudano, E., Legg, C.R., Glickstein, M., 1991. Afferent and Efferent Connections of Temporal Association Cortex in the Rat: A Horseradish Peroxidase Study. European Journal of Neuroscience 3, 317–330. https://doi.org/10.1111/j.1460-9568.1991.tb00818.x

Velasco, M., Velasco, F., Almanza, X., Coats, A.C., 1982. Subcortical correlates of the auditory brain stem potentials in man: Bipolar EEG and multiple unit activity and electrical stimulation. Electroencephalography and Clinical Neurophysiology 53, 133–142. https://doi.org/10.1016/0013-4694(82)90019-0

Velasco, M., Velasco, F., Almanza, X., Coats, A.C., 1981. Subcortical correlates of the auditory brain stem potentials in man: Referential EEG responses. International Journal of Neuroscience 15, 241–248. https://doi.org/10.3109/00207458108985861

Velasco, M., Velasco, F., Velasco, A.L., Almanza, X., Olvera, A., 1986. Subcortical correlates of the P300 potential complex in man to auditory stimuli. Electroencephalography and Clinical Neurophysiology 64, 199–210. https://doi.org/10.1016/0013-4694(86)90166-5

Vinnik, E., Antopolskiy, S., Itskov, P.M., Diamond, M.E., 2012. Auditory stimuli elicit hippocampal neuronal responses during sleep. Frontiers in Systems Neuroscience 6. https://doi.org/10.3389/fnsys.2012.00049

Vinogradova, O.S., 2001. Hippocampus as comparator: Role of the two input and two output systems of the hippocampus in selection and registration of information. Hippocampus 11, 578–598. https://doi.org/10.1002/hipo.1073

Vinogradova, O.S., 1970. Registration of information and the limbic system, in: Horn, G., Hinde, R.A. (Eds.), Short-Term Changes in Neural Activity and Behaviour. Cambridge University Press, London, UK.

Vinogradova, O.S., Brazhnik, E.S., Kitchigina, V.F., Stafekhina, V.S., 1993. Acetylcholine, theta-rhythm and activity of hippocampal neurons in the rabbit—IV. Sensory stimulation. Neuroscience 53, 993–1007. https://doi.org/10.1016/0306-4522(93)90484-W

Vinogradova, O.S., Semyonova, T.P., Konovalov, V.Ph., 1970. Trace phenomena in single neurons of hippocampus and mammiliary bodies, in: Biology of Memory. Elsevier, pp. 191–221. https://doi.org/10.1016/B978-0-12-564350-4.50021-X

Wan, F.-J., Caine, S.B., Swerdlow, N.R., 1996. The ventral subiculum modulation of prepulse inhibition is not mediated via dopamine D2 or nucleus accumbens non-NMDA glutamate receptor activity. European Journal of Pharmacology 314, 9–18. https://doi.org/10.1016/S0014-2999(96)00535-3

Wang, J., Bast, T., Wang, Y.-C., Zhang, W.-N., 2015. Hippocampus and two-way active avoidance conditioning: Contrasting effects of cytotoxic lesion and temporary inactivation: HIPPOCAMPUS AND TWO-WAY ACTIVE AVOIDANCE CONDITIONING. Hippocampus 25, 1517–1531. https://doi.org/10.1002/hipo.22471

Wang, N., Gan, X., Liu, Y., Xiao, Z., 2017. Balanced Noise-Evoked Excitation and Inhibition in Awake Mice CA3. Frontiers in Physiology 8. https://doi.org/10.3389/fphys.2017.00931

Wang, R.-Y., Phang, R.-Z., Hsu, P.-H., Wang, W.-H., Huang, H.-T., Liu, I.Y., 2013. In vivo knockdown of hippocampal miR-132 expression impairs memory acquisition of trace fear conditioning. Hippocampus 23, 625–633. https://doi.org/10.1002/hipo.22123

Wang, S., Yu, Y., Feng, Y., Zou, F., Zhang, X., Huang, J., Zhang, Y., Zheng, X., Huang, X.-F., Zhu, Y., Liu, Y., 2016. Protective effect of the orientin on noise-induced cognitive impairments in mice. Behavioural Brain Research 296, 290–300. https://doi.org/10.1016/j.bbr.2015.09.024

Warren, D.E., Duff, M.C., 2014. Not so fast: Hippocampal amnesia slows word learning despite successful fast mapping: Amnesia Slows Word Learning Despite Fast Mapping. Hippocampus 24, 920–933. https://doi.org/10.1002/hipo.22279

Warren, D.E., Tranel, D., Duff, M.C., 2016. Impaired acquisition of new words after left temporal lobectomy despite normal fast-mapping behavior. Neuropsychologia 80, 165–175. https://doi.org/10.1016/j.neuropsychologia.2015.11.016

Watanabe, T., Yagishita, S., Kikyo, H., 2008. Memory of music: Roles of right hippocampus and left inferior frontal gyrus. NeuroImage 39, 483–491. https://doi.org/10.1016/j.neuroimage.2007.08.024

Weible, A.P., O’Reilly, J.-A., Weiss, C., Disterhoft, J.F., 2006. Comparisons of dorsal and ventral hippocampus cornu ammonis region 1 pyramidal neuron activity during trace eye-blink conditioning in the rabbit. Neuroscience 141, 1123–1137. https://doi.org/10.1016/j.neuroscience.2006.04.065

Weiss, C., Kronforst-Collins, M.A., Disterhoft, J.F., 1996. Activity of hippocampal pyramidal neurons during trace eyeblink conditioning. Hippocampus 6, 192–209. https://doi.org/10.1002/(SICI)1098-1063(1996)6:2<192::AID-HIPO9>3.0.CO;2-R

Weisz, D.J., Clark, G.A., Thompson, R.F., 1984. Increased responsivity of dentate granule cells during nictitating membrane response conditioning in rabbit. Behavioural Brain Research 12, 145–154. https://doi.org/10.1016/0166-4328(84)90037-8

Weitz, A.J., Fang, Z., Lee, H.J., Fisher, R.S., Smith, W.C., Choy, M., Liu, J., Lin, P., Rosenberg, M., Lee, J.H., 2015. Optogenetic fMRI reveals distinct, frequency-dependent networks recruited by dorsal and intermediate hippocampus stimulations. NeuroImage 107, 229–241. https://doi.org/10.1016/j.neuroimage.2014.10.039

West, M.O., Christian, E., Robinson, J.H., Deadwyler, S.A., 1982. Evoked potentials in the dentate gyrus reflect the retention of past sensory events. Neuroscience Letters 28, 319–324.

West, M.O., Christian, E., Robinson, J.H., Deadwyler, S.A., 1981. Dentate granule cell discharge during conditioning: Relation to movement and theta rhythm. Experimental Brain Research 44. https://doi.org/10.1007/BF00236566

Whishaw, I.Q., 1976. The effects of alcohol and atropine on EEG and behavior in the rabbit. Psychopharmacology 48, 83–90. https://doi.org/10.1007/BF00423311

Whishaw, I.Q., 1972. Hippocampal electroencephalographic activity in the Mongolian gerbil during natural behaviours and wheel running and in the rat during wheel running and conditioned immobility. Can J Psychol 26, 219–239. https://doi.org/10.1037/h0082431

Whishaw, I.Q., Dyck, R., 1984. Comparative Potency of Tactile, Auditory, and Visual Stimulus Repetition in Eliciting Activated Forebrain EEG in the Rabbit. Behavioral Neuroscience 98, 333–344.

Wickelgren, W.A., 1968. Sparing of short-term memory in an amnesic patient: Implications for strength theory of memory 10.

Wilkins, R.W., Hodges, D.A., Laurienti, P.J., Steen, M., Burdette, J.H., 2014. Network Science and the Effects of Music Preference on Functional Brain Connectivity: From Beethoven to Eminem. Scientific Reports 4. https://doi.org/10.1038/srep06130

Wilmot, J.H., Puhger, K., Wiltgen, B.J., 2019. Acute Disruption of the Dorsal Hippocampus Impairs the Encoding and Retrieval of Trace Fear Memories. Frontiers in Behavioral Neuroscience 13. https://doi.org/10.3389/fnbeh.2019.00116

Wilson, C.L., Babb, T.L., Halgren, E., Wang, M.L., Crandall, P.H., 1984. Habituation of human limbic neuronal response to sensory stimulation. Experimental Neurology 84, 74–97. https://doi.org/10.1016/0014-4886(84)90007-4

Winocur, G., Rawlins, J.N.P., Gray, J.A., 1987. The Hippocampus and Conditioning to Contextual Cues. Behavioral Neuroscience 101, 617–625.

Wirtshafter, H.S., Wilson, M.A., 2019. Locomotor and Hippocampal Processing Converge in the Lateral Septum. Current Biology 29, 3177-3192.e3. https://doi.org/10.1016/j.cub.2019.07.089

Witt, J.-A., Coras, R., Schramm, J., Becker, A.J., Elger, C.E., Blümcke, I., Helmstaedter, C., 2014. The overall pathological status of the left hippocampus determines preoperative verbal memory performance in left mesial temporal lobe epilepsy: Hippocampal Neuronal Cell Densities and Memory Functions. Hippocampus 24, 446–454. https://doi.org/10.1002/hipo.22238

Wu, G., Yao, J., Hu, B., Zhang, H., Li, Y., Li, X., Li, Q., Sui, J., 2013. Reevaluating the Role of the Hippocampus in Delay Eyeblink Conditioning. PLoS ONE 8, e71249. https://doi.org/10.1371/journal.pone.0071249

Xiao, C., Liu, Y., Xu, J., Gan, X., Xiao, Z., 2018. Septal and Hippocampal Neurons Contribute to Auditory Relay and Fear Conditioning. Frontiers in Cellular Neuroscience 12. https://doi.org/10.3389/fncel.2018.00102

Xing, Y., Chen, W., Wang, Y., Jing, W., Gao, S., Guo, D., Xia, Y., Yao, D., 2016a. Music exposure improves spatial cognition by enhancing the BDNF level of dorsal hippocampal subregions in the developing rats. Brain Research Bulletin 121, 131–137. https://doi.org/10.1016/j.brainresbull.2016.01.009

Xing, Y., Xia, Y., Kendrick, K., Liu, X., Wang, M., Wu, D., Yang, H., Jing, W., Guo, D., Yao, D., 2016b. Mozart, Mozart Rhythm and Retrograde Mozart Effects: Evidences from Behaviours and Neurobiology Bases. Scientific Reports 6. https://doi.org/10.1038/srep18744

Yakunina, N., Tae, W.S., Lee, K.U., Kim, S.S., Nam, E.-C., 2013. Spatiotemporal Segregation of Neural Response to Auditory Stimulation: An fMRI Study Using Independent Component Analysis and Frequency-Domain Analysis. PLoS ONE 8, e66424. https://doi.org/10.1371/journal.pone.0066424

Yang, J., Hu, L., Song, T., Liu, Y., Wu, Q., Zhao, L., Liu, L., Zhao, X., Zhang, D., Huang, C., 2014a. Proteomic Changes in Female Rat Hippocampus Following Exposure to a Terrified Sound Stress. Journal of Molecular Neuroscience 53, 158–165. https://doi.org/10.1007/s12031-014-0242-6

Yang, J., Hu, L., Wu, Q., Liu, L., Zhao, L., Zhao, X., Song, T., Huang, C., 2014b. A terrified-sound stress induced proteomic changes in adult male rat hippocampus. Physiology & Behavior 128, 32–38. https://doi.org/10.1016/j.physbeh.2014.01.038

Yoon, T., Otto, T., 2007. Differential contributions of dorsal vs. ventral hippocampus to auditory trace fear conditioning. Neurobiology of Learning and Memory 87, 464–475. https://doi.org/10.1016/j.nlm.2006.12.006

Yoshii, N., Shimokochi, M., Miyamoto, K., Ito, M., 1966. Studies on the Neural Basis of Behavior by Continuous Frequency Analysis of EEG, in: Progress in Brain Research. Elsevier, pp. 217–250. https://doi.org/10.1016/S0079-6123(08)62979-9

Yoshiura, T., Zhong, J., Shibata, D.K., Kwok, W.E., Shrier, D.A., Numaguchi, Y., 1999. Functional MRI study of auditory and visual oddball tasks: NeuroReport 10, 1683–1688. https://doi.org/10.1097/00001756-199906030-00011

Yu, C., Moss, C.F., 2022. Natural acoustic stimuli evoke selective responses in the hippocampus of passive listening bats. Hippocampus hipo.23407. https://doi.org/10.1002/hipo.23407

Yu, Y.-F., Zhai, F., Dai, C.-F., Hu, J.-J., 2011. The relationship between age-related hearing loss and synaptic changes in the hippocampus of C57BL/6J mice. Experimental Gerontology 46, 716–722. https://doi.org/10.1016/j.exger.2011.04.007

Zatorre, R.J., 1985. Discrimination and recognition of tonal melodies after unilateral cerebral excisions. Neuropsychologia 23.

Zatorre, R.J., Samson, S., 1991. Role of the right temporal neocortex in retention of pitch in auditory short-term memory. Brain 114, 2403–2417. https://doi.org/10.1093/brain/114.6.2403

Zevin, J.D., McCandliss, B.D., 2005. Dishabituation of the BOLD response to speech sounds. Behavioral and Brain Functions 12.

Zhang, G.-W., Sun, W.-J., Zingg, B., Shen, L., He, J., Xiong, Y., Tao, H.W., Zhang, L.I., 2018. A Non-canonical Reticular-Limbic Central Auditory Pathway via Medial Septum Contributes to Fear Conditioning. Neuron 97, 406-417.e4. https://doi.org/10.1016/j.neuron.2017.12.010

Zhang, L., Wu, C., Martel, D.T., West, M., Sutton, M.A., Shore, S.E., 2021. Noise Exposure Alters Glutamatergic and GABAergic Synaptic Connectivity in the Hippocampus and Its Relevance to Tinnitus. Neural Plasticity 2021, 1–16. https://doi.org/10.1155/2021/8833087

Zhang, L., Wu, C., Martel, D.T., West, M., Sutton, M.A., Shore, S.E., 2018. Remodeling of cholinergic input to the hippocampus after noise exposure and tinnitus induction in Guinea pigs. Hippocampus hipo.23058. https://doi.org/10.1002/hipo.23058

Zhang, Y., Zhu, M., Sun, Y., Tang, B., Zhang, G., An, P., Cheng, Y., Shan, Y., Merzenich, M.M., Zhou, X., 2021. Environmental noise degrades hippocampus-related learning and memory. Proc Natl Acad Sci USA 118, e2017841117. https://doi.org/10.1073/pnas.2017841117

Zhao, H., Wang, L., Chen, Liang, Zhang, J., Sun, W., Salvi, R.J., Huang, Y.-N., Wang, M., Chen, Lin, 2018. Temporary conductive hearing loss in early life impairs spatial memory of rats in adulthood. Brain and Behavior 8, e01004. https://doi.org/10.1002/brb3.1004

Zheng, J., Suo, L., Zhou, Y., Jia, L., Li, J., Kuang, Y., Cui, D., Zhang, X., Wu, Q., 2022. Pyk2 suppresses contextual fear memory in an autophosphorylation-independent manner. Journal of Molecular Cell Biology 13, 808–821. https://doi.org/10.1093/jmcb/mjab057

Zhong, Y.-M., Yukie, M., Rockland, K.S., 2005. Direct projections from CA1 to the superior temporal sulcus in the monkey, revealed by single axon analysis. Brain Research 1035, 211–214. https://doi.org/10.1016/j.brainres.2004.12.010
